# Supplementary material for: SEGUID v2: Extending SEGUID checksums for circular, linear, single- and double-stranded biological sequences
Source: PLoS One. 2026 Aug 7;21(8):e0346164. doi: 10.1371/journal.pone.0346164 (PMC13450835; doi:10.1371/journal.pone.0346164)
Supplement: Supplementary file 1 [file pone.0346164.s001.pdf]

Supplementary Notes

Example Sequences used in SEGUID v2 Manuscript

Introduction

This document gives details of the nine longer biological sequences mentioned in Section 1, Section 2.1.3, and Section 2.1.4, and Table 1 in the manuscript. We use Bash to download the sequence files, and R to produce SEGUID v2 checksums, etc.

Download

The attached data was downloaded, or created, using:

```
mkdir -p data/{manual,nuccore,nucleotide,protein}

# AAF20475.1 and AAF20481.1 (Section 1)
curl --silent -L --output "data/protein/AAF20475.1.fasta" \
"https://www.ncbi.nlm.nih.gov/sviewer/viewer.fcgi?\
id=AAF20475.1&db=protein&report=fasta&retmode=text"

curl --silent -L --output "data/protein/AAF20481.1.fasta" \
"https://www.ncbi.nlm.nih.gov/sviewer/viewer.fcgi?\
id=AAF20481.1&db=protein&report=fasta&retmode=text"

# Q9X2V7 variants (Section 2.1.3)
echo "GGAGHVPEYFVGIGTPISFYG" > data/manual/Q9X2V7_p38-58.asis
echo "VGIGTPISFYGGGAGHVPEYF" > data/manual/Q9X2V7_p38-58-alt.asis

# NC_003287.2, V00604.2 and MW729338 (Section 2.1.3)
curl --silent -L --output "data/nuccore/NC_003287.2.fasta" \
"https://www.ncbi.nlm.nih.gov/sviewer/viewer.fcgi?\
id=NC_003287&db=nuccore&report=fasta&retmode=text"
```

```
curl --silent -L --output "data/nuccore/V00604.2.fasta" \
"https://www.ncbi.nlm.nih.gov/sviewer/viewer.fcgi?\
id=V00604.2&db=nuccore&report=fasta&retmode=text"

curl --silent -L --output "data/nuccore/MW729338.fasta" \
"https://www.ncbi.nlm.nih.gov/sviewer/viewer.fcgi?\
id=MW729338&db=nuccore&report=fasta&retmode=text"

# M77789.2 and L09137.2 (Section 2.1.4)
curl --silent -L --output "data/nucleotide/M77789.2.fasta" \
"https://www.ncbi.nlm.nih.gov/sviewer/viewer.fcgi?\
id=M77789.2&db=nucleotide&report=fasta&retmode=text"

curl --silent -L --output "data/nucleotide/L09137.2.fasta" \
"https://www.ncbi.nlm.nih.gov/sviewer/viewer.fcgi?\
id=L09137.2&db=nucleotide&report=fasta&retmode=text"
```

The downloaded files are:

```
tree data/
```

```
data/
  manual
     AAD28494.1_p38-58-alt.asis
     AAD28494.1_p38-58.asis
  nuccore
     MW729338.1.fasta
     NC_003287.2.fasta
     V00604.2.fasta
  nucleotide
     L09137.2.fasta
     M77789.2.fasta
  protein
      AAF20475.1.fasta
      AAF20481.1.fasta

5 directories, 9 files
```

SEGUID v2 using R

We start by defining a few utility functions in R:

```r
print.seq <- function(seq) {
  n <- nchar(seq)
  message(sprintf("Sequence: [n=%d bps] %s...%s",
                  n, substr(seq, 1, 6), substr(seq, n-5, n)))
}

reverse_complementary <- function(seq) {
  seq <- strsplit(seq, split = "", fixed = TRUE)[[1]]
  seq <- rev(seq)
  seq <- chartr("ACGT", "TGCA", seq)
  seq <- paste(seq, collapse = "")
  seq <- structure(seq, class = "seq")
  seq
}

read_seq <- function(id, db, type = c("fasta", "asis")) {
  type <- match.arg(type)
  file <- file.path("data", db, paste(id, type, sep = "."))
  seq <- readLines(file)
  if (type == "fasta") {
    seq <- grep("^>", seq, value = TRUE, invert = TRUE)
    seq <- paste(seq, collapse = "")
  }
  seq <- structure(seq, class = "seq")
  seq
}
```

Section 1 ("Introduction")

"CRC-64 clash" (AAF20475.1 and AAF20481.1)

```r
seq <- read_seq("AAF20475.1", db = "protein", type = "fasta")
seq
```

```
Sequence: [n=110 bps] QSALTQ...TKLTVL
```

```r
seguid::lsseguid(seq, alphabet = "{protein}")
```

```
[1] "lsseguid=X5XEaayob1nZLOc7eVT9qyczarY"
```

```r
seq <- read_seq("AAF20481.1", db = "protein", type = "fasta")
seq
```

```
Sequence: [n=110 bps] QSALTQ...TKLTVL
```

```r
seguid::lsseguid(seq, alphabet = "{protein}")
```

```
[1] "lsseguid=BpBeDdcNUYNsdk46JoJdw7Pd3BI"
```

Section 2.1.3 ("Circular single-stranded sequences")

M13 bacteriophage (NC_003287.2 and V00604.2)

```r
seq <- read_seq("NC_003287.2", db = "nuccore", type = "fasta")
seq
```

```
Sequence: [n=6407 bps] AACGCT...GATGTT
```

```r
seguid::csseguid(seq, alphabet = "{protein}")
```

```
[1] "csseguid=aAjgnsF9cPI6cu8IQ81sYnstVzU"
```

```r
seq <- read_seq("V00604.2", db = "nuccore", type = "fasta")
seq
```

```
Sequence: [n=6407 bps] AACGCT...GATGTT
```

```r
seguid::csseguid(seq, alphabet = "{protein}")
```

```
[1] "csseguid=aAjgnsF9cPI6cu8IQ81sYnstVzU"
```

circRNA from Culex pipiens (MW729338)

```
seq <- read_seq("MW729338.1", db = "nuccore", type = "fasta")
seq
```

```
Sequence: [n=840 bps] GTATCC...AAATTG
```

```
seguid::csseguid(seq, alphabet = "{protein}")
```

```
[1] "csseguid=HJMksLWFt-alpfsZ-LWYravFvJA"
```

Microcin J25 (two AAD28494.1 variants)

```
seq <- read_seq("AAD28494.1_p38-58", db = "manual", type = "asis")
seq
```

```
Sequence: [n=21 bps] GGAGHV...PISFYG
```

```
seguid::csseguid(seq, alphabet = "{protein}")
```

```
[1] "csseguid=9AzSHhbB-vv-SVa6j99yW4-uDoU"
```

```
seq <- read_seq("AAD28494.1_p38-58-alt", db = "manual", type = "asis")
seq
```

```
Sequence: [n=21 bps] VGIGTP...HVPEYF
```

```
seguid::csseguid(seq, alphabet = "{protein}")
```

```
[1] "csseguid=9AzSHhbB-vv-SVa6j99yW4-uDoU"
```

Section 2.1.4: ("Circular double-stranded sequences")

pUC19 (M77789.2 and L09137.2)

```r
watson <- read_seq("M77789.2", db = "nucleotide", type = "fasta")
watson
```

```
Sequence: [n=2686 bps] GCGCCC...GGAAGA
```

```r
crick <- reverse_complementary(watson)
crick
```

```
Sequence: [n=2686 bps] TCTTCC...GGGCGC
```

```r
seguid::cdseguid(watson, crick, alphabet = "{DNA}")
```

```
[1] "cdseguid=mCC0B3UMZfgLyh3Pl574MVjm30U"
```

```r
watson <- read_seq("L09137.2", db = "nucleotide", type = "fasta")
watson
```

```
Sequence: [n=2686 bps] TCGCGC...TTCGTC
```

```r
crick <- reverse_complementary(watson)
crick
```

```
Sequence: [n=2686 bps] GACGAA...GCGCGA
```

```r
seguid::cdseguid(watson, crick, alphabet = "{DNA}")
```

```
[1] "cdseguid=mCC0B3UMZfgLyh3Pl574MVjm30U"
```

Section 2.3: ("Encoding (Base64url)")

Table 1

```r
seq <- "ACAGATT"

sha1_base16 <- digest::digest(seq, algo = "sha1", serialize = FALSE)
sha1_base16
```

```
[1] "9adaef6edbb0afafcca01c6fb66e01129bfe8ca4"
```

```r
sha1_base64 <- base64enc::base64encode(
  digest::digest(seq, algo = "sha1", serialize = FALSE, raw = TRUE)
)
sha1_base64
```

```
[1] "mtrvbtuwr6/MoBxvtm4BEpv+jKQ="
```

```r
sha1_base64url <- chartr("/+", "_-", sha1_base64)
sha1_base64url
```

```
[1] "mtrvbtuwr6_MoBxvtm4BEpv-jKQ="
```

```r
seguid <- seguid::seguid(seq, alphabet = "{DNA}")
seguid
```

```
[1] "seguid=mtrvbtuwr6/MoBxvtm4BEpv+jKQ"
```

```r
seguid2 <- seguid::lsseguid(seq, alphabet = "{DNA}")
seguid2
```

```
[1] "lsseguid=mtrvbtuwr6_MoBxvtm4BEpv-jKQ"
```

Appendix

Session information

```r
sessionInfo()
```

```
R version 4.5.1 (2025-06-13)
Platform: x86_64-pc-linux-gnu
Running under: Ubuntu 24.04.3 LTS

Matrix products: default
BLAS:   /home/hb/shared/software/CBI/_ubuntu24_04/R-4.5.1-gcc13/lib/R/lib/libRblas.so
```

```
LAPACK: /usr/lib/x86_64-linux-gnu/lapack/liblapack.so.3.12.0  LAPACK version 3.12.0

locale:
 [1] LC_CTYPE=en_US.UTF-8       LC_NUMERIC=C
 [3] LC_TIME=en_US.UTF-8        LC_COLLATE=en_US.UTF-8
 [5] LC_MONETARY=en_US.UTF-8    LC_MESSAGES=en_US.UTF-8
 [7] LC_PAPER=en_US.UTF-8       LC_NAME=C
 [9] LC_ADDRESS=C               LC_TELEPHONE=C
[11] LC_MEASUREMENT=en_US.UTF-8 LC_IDENTIFICATION=C

time zone: America/Los_Angeles
tzcode source: system (glibc)

attached base packages:
[1] stats     graphics  grDevices utils     datasets  methods   base

loaded via a namespace (and not attached):
 [1] compiler_4.5.1    fastmap_1.2.0     cli_3.6.5         htmltools_0.5.8.1
 [5] tools_4.5.1       base64enc_0.1-3   seguid_0.1.0      yaml_2.3.10
 [9] rappdirs_0.3.3    rmarkdown_2.29    progressr_0.15.1  knitr_1.50
[13] jsonlite_2.0.0    xfun_0.53         digest_0.6.37     rlang_1.1.6
[17] evaluate_1.0.5
```
