# Supplementary material for: SEGUID v2: Extending SEGUID checksums for circular, linear, single- and double-stranded biological sequences
Source: PLoS One. 2026 Aug 7;21(8):e0346164. doi: 10.1371/journal.pone.0346164 (PMC13450835; doi:10.1371/journal.pone.0346164)
Supplement: Supplementary file 2 [file pone.0346164.s003.pdf]

PONE-D-24-51050

SEGUID v2: Extending SEGUID checksums for circular, linear, single- and double-stranded biological sequences

Journal Requirements

When submitting your revision, we need you to address these additional requirements.

1. Please ensure that your manuscript meets PLOS ONE's style requirements, including those for file naming.

The PLOS ONE style templates can be found at https://journals.plos.org/plosone/s/file?id=wjVg/PLOSOne_formatting_sample_main_body.pdf and https://journals.plos.org/plosone/s/file?id=ba62/PLOSOne_formatting_sample_title_authors_affiliations.pdf

Authors' response: We have converted our LaTeX document to use the PLOS ONE LaTeX template provided at https://journals.plos.org/plosone/s/latex. The style and layout of our PDF matches that of the example PDF provided by PLOS ONE.

Please note, our original PLOS ONE submission was based on our submission to PLOS Computational Biology, which then was transferred to PLOS ONE. Given that we now have migrated the manuscript to the PLOS ONE LaTeX template, it is not feasible for us to upload track changes. We're sorry about that, but we have clarified below what our changes are.

2. Please note that PLOS ONE has specific guidelines on code sharing for submissions in which author-generated code underpins the findings in the manuscript. In these cases, all author-generated code must be made available without restrictions upon publication of the work.

Please review our guidelines at https://journals.plos.org/plosone/s/materials-and-software-sharing#loc-sharing-code and ensure that your code is shared in a way that follows best practice and facilitates reproducibility and reuse.

Authors' response: Our source code is available under the GitHub organization https://github.com/seguid. We have made official releases of the relevant SEGUID implementations and obtained corresponding DOI:s from Zenodo:

- seguid-calculator, https://doi.org/10.5281/zenodo.17010386
- seguid-javascript, https://doi.org/10.5281/zenodo.17010380
- seguid-python, https://doi.org/10.5281/zenodo.17010394
- seguid-r, https://doi.org/10.5281/zenodo.17010375
- seguid-tcl, https://doi.org/10.5281/zenodo.17010377

All relevant data (sequence data examples) are within the manuscript and its Supporting Information files (ZIP file).

3. We note that the grant information you provided in the 'Funding Information' and 'Financial Disclosure' sections do not match.

Authors' response: We do not understand what you are referring to here, but if you mean that the 'Funding Information' entered in the PLOS ONE submission form did not match the information we previously had in the 'Acknowledgement' section, then the latter has been removed per your request (see below). If there is any other place were we should enter or specify a 'Financial Disclosure', please let us know - we do not see such a field in the PLOS ONE online submission.

4. Please note that funding information should not appear in the Acknowledgments section or other areas of your manuscript.

We will only publish funding information present in the Funding Statement section of the online submission form. Please remove any funding-related text from the manuscript.

Authors' response: We have removed the funding information from the Acknowledgments section in the manuscript. The first version stemmed from our original submission to PLOS Computational Biology, which then transferred the manuscript to PLOS ONE.

5. Thank you for uploading your study's underlying data set. Unfortunately, the repository you have noted in your Data Availability statement does not qualify as an acceptable data repository according to PLOS's standards.

At this time, please upload the minimal data set necessary to replicate your study's findings to a stable, public repository (such as figshare or Dryad) and provide us with the relevant URLs, DOIs, or accession numbers that may be used to access these data. For a list of recommended repositories and additional information on PLOS standards for data deposition, please see https://journals.plos.org/plosone/s/recommended-repositories.

Authors' response: All data used in the manuscript is available in the Supplementary Data ZIP file uploaded together with the manuscript. In the revision, we now explicitly specify "All relevant data are within the manuscript and its Supporting Information files." We have removed the reference to https://www.seguid.org originally used as a repository.

6. We note that Figures 8 and 9 in your submission contain copyrighted images.

All PLOS content is published under the Creative Commons Attribution License (CC BY 4.0), which means that the manuscript, images, and Supporting Information files will be freely available online, and any third party is permitted to access, download, copy, distribute, and use these materials in any way, even commercially, with proper attribution. For more information, see our copyright guidelines: http://journals.plos.org/plosone/s/licenses-and-copyright.

Authors' response: We understood this as there was a presence of commercial logotypes in these two figures, which has now been removed.

The old version of Figure 8, a screenshot of a presentation by co-author György Babnigg, contained the Argonne National Laboratory logotype, the Midwest Center for Structural Genomics (MCSG) logotype and the Protein Structure Initiative (PSI) logotype. These have been removed in the new Figure 8.

Figure 9 contain the "A plasmid Editor" (ApE) icon and the pydna icon. The latter was made up from parts for which we did not have ownership. The copyright holder for the new pydna icon is Björn Johansson, co-author to this manuscript. We have uploaded the requested forms.

Please upload the completed Content Permission Form or other proof of granted permissions as an ""Other"" file with your submission.

Authors' response: The remaining artifacts are copyrighted to co-authors of this manuscript. Given the above adjustments, we deemed this suggested statement unnecessary. If we need to sign Content Permission Forms for co-authors own work, please let us know.

8. We are unable to open your Supporting Information file [Pereira_etal_2024-SEGUIDv2-SupplData.zip]. Please kindly revise as necessary and re-upload.

Authors' response: We are not sure what is going on, but we suspect it is something on your end. We have verified that the ZIP file is indeed valid and complete. We did this by downloading the file from the PLOS portal via 'Author login' -> 'Submissions Needing Revision' -> 'Action Links' -> 'File Inventory' -> 'Download'. The downloaded file 'Pereira_etal_2024-SEGUIDv2-SupplData.zip' (Oct 30 2024 6:34PM) is 9,638 bytes large (md5sum ec75f42f41d3cede43cf471f210af8f4). Unzipping the file using `unzip Pereira_etal_2024-SEGUIDv2-SupplData.zip` extracts a directory `data/` with the expected nice sequence files.

Through this exercise, we noticed that one of the files uses a UniProt identifier whereas the others uses GenBank identifiers. For consistency, we renamed this file. Because of this, we have uploaded a slightly modified ZIP file, which we have also verified can be unzipped. The new file (9,658 bytes; md5sum afb9eca6f40ef7c2c8a6073e48d52835) contains:

```
$ unzip -v Pereira_etal_2024-SEGUIDv2-SupplData.zip
Archive:  Pereira_etal_2024-SEGUIDv2-SupplData.zip
 Length   Method    Size  Cmpr    Date    Time   CRC-32   Name
--------  ------  ------- ---- ---------- ----- --------  ----
       0  Stored        0   0% 2025-08-30 13:25 00000000  data/
       0  Stored        0   0% 2025-08-30 13:25 00000000  data/protein/
     199  Defl:N      173  13% 2025-08-30 13:25 3debe0ce  data/protein/AAF20475.1.fasta
     199  Defl:N      173  13% 2025-08-30 13:25 86373871  data/protein/AAF20481.1.fasta
       0  Stored        0   0% 2025-08-30 14:04 00000000  data/manual/
      22  Stored       22   0% 2025-08-30 13:25 512fa260  data/manual/AAD28494.1_p38-58-a
      22  Stored       22   0% 2025-08-30 13:25 fb35103b  data/manual/AAD28494.1_p38-58.a
       0  Stored        0   0% 2025-08-30 13:25 00000000  data/nucleotide/
    2776  Defl:N     1020  63% 2025-08-30 13:25 66cb8af7  data/nucleotide/M77789.2.fasta
    2777  Defl:N     1015  63% 2025-08-30 13:25 b476ded0  data/nucleotide/L09137.2.fasta
       0  Stored        0   0% 2025-08-30 14:05 00000000  data/nuccore/
    6527  Defl:N     2157  67% 2025-08-30 13:25 f647a886  data/nuccore/V00604.2.fasta
     914  Defl:N      397  57% 2025-08-30 13:25 5b81b0e9  data/nuccore/MW729338.1.fasta
    6555  Defl:N     2195  67% 2025-08-30 13:25 56d247cb  data/nuccore/NC_003287.2.fasta
--------          -------  ---                            -------
   19991             7174  64%                            14 files
```

Response to Reviewers

Reviewer 1

1. Clarify the Abstract: The abstract could be more structured. Consider clearly summarizing the background, objectives, methods, results, and conclusions to make it more reader-friendly.

Authors' response: The abstract is currently divided into Background, Objectives, Conclusions and Availability. As each part is rather short, we feel that adding more information here might muddle the abstract rather than clarify.

2. Enhance the Introduction: Add more context to the introduction by explaining the importance of SEGUID v2 in synthetic biology and how it addresses current challenges.

Authors' response: We added the following to the end of the introduction to address this point: "We believe that a stable checksum that describes any biological sequence, regardless of topology or strandedness, would streamline the exchange of information and biological materials within the synthetic biology community."

3. Define Technical Terms: Simplify or define technical terms like "checksums" and "topological differences" for readers who may not have a technical background.

Authors' response: We have changed the text in the Objectives section of the abstract:

"The original SEGUID, while effective for protein sequences and single-stranded DNA (ssDNA), is not suitable for circular and double-stranded DNA (dsDNA) due to topological differences."

to

"The original SEGUID, while effective for protein sequences and single-stranded DNA (ssDNA), is not suitable for circular DNA since there is no natural starting position nor for double-stranded DNA (dsDNA) since two separate sequences are present."

This change replaces the somewhat opaque phrase "topological differences". As for the term "checksum", we think that this is the simplest term available and it a well established term. We feel that synonyms like "hash function" would be harder to understand for readers from non-technical background.

4. Expand the Methodology: Provide more detail in the methodology section, especially about the algorithms used and how they ensure unique representations.

Authors' response: The new SEGUID checksums provide unique representation for all biological sequences described through two conventions:

1. The use of smallest string rotation as a starting point.
2. Using a semicolon to separate the 5′ -> 3′ Watson sequence and the 5′ -> 3′ Crick sequences prior to calculating checksums for double stranded sequences.

We feel that these points come across in the current manuscript.

5. Include Visual Aids: Diagrams or flowcharts illustrating the SEGUID v2 process would make the paper easier to understand and more engaging.

Authors' response: The manuscript currently contains ten figures serving this purpose. We do not believe adding more would enhance the reader's understanding. A re-review of the figures, did not reveal any superfluous figures that would improve the manuscript, if left out.

6. Discuss Limitations: Include a section that openly discusses SEGUID v2's limitations and suggests directions for future research.

Authors' response: We discuss advantages and drawbacks of the SHA-1 (Secure Hash Algorithm 1) algorithm and how this remains the best choice balancing length, resistance to collisions and availability across different computer systems. The 160-bit output provides a compact yet reliable identifier for data identification, and its widespread implementation has ensured compatibility across diverse systems. One notable application of SHA-1 is in the Git version control system, where it is used to uniquely identify file content.

7. Strengthen the Results Section: Add more quantitative data to back up the claims about the algorithm's effectiveness.

Authors' response: The first part of the Results section focuses heavily on the correctness of the algorithm and not so much on the effectiveness (speed, computational efficiency). We extensively discuss edge cases that may be of less importance currently, but this might change in the future. We believe that, at this point, correctness and completeness are more important, since changing the algorithm later could be costly. We think this is the correct priority as computationally efficient algorithms may be devised later as the need arises.

8. Add Comparative Analysis: Compare SEGUID v2 with other checksum algorithms to highlight its unique advantages.

Authors' response: As noted in Response 6, we believe this point is addressed. The novel nature of the checksum makes comparisons, other than the ones we made motivating the choice of the SHA-1 algorithm, of limited value.

9. Clarify Data Availability: Be specific about the types of data available and how readers can access them.

Authors' response: We direct the reader to the GitHub repository and the website associated with this project. We maintain the easy to remember seguid.org domain where we offer implementations for six popular programming languages. We also offer a very simple web interface as well as a SEGUID functionality in popular ApE DNA editor (Figure 7).

10. Improve Language Consistency: Ensure terms and terminology are used consistently, especially when discussing different types of sequences.

Authors' response: A careful reading of the manuscript did not reveal to us cases of inconsistent terminology.

11. Refine the Conclusion: Summarize the key findings and their implications for the field in a concise and impactful way.

Authors' response: Under section 6 Discussions, we conclude the main advantages compared to other methods. The details of these conclusions are listed in section 5 Applications.

12. Add References: Ensure all statements and claims are backed up with proper references, especially in the introduction and discussion sections.

Authors' response: We have 33 references that we feel adequately support the claims, using the oldest available references where several relevant ones were available.

13. Improve Formatting: Review the document to ensure consistent formatting across headings, subheadings, and reference styles.

Authors' response: The document is produced using LaTeX which ensures consistent formatting.

14. Clarify Acronyms: Define all acronyms when they first appear to make the content accessible to all readers.

Authors' response: The lists below show the acronyms used in the manuscript. The ones marked as revised below were changed in the revised manuscript to include a definition when first used:

- **SEGUID** - Defined on first use (no change)
- **SHA-1** - Defined as "Secure Hash Algorithm 1"; moved to first mention. (revised)
- **CRC** / **CRC-64** - Defined on first use (no change)
- **ssDNA** - Defined on first use (no change)
- **dsDNA** - Defined on first use (no change)
- **circRNA** - Defined on first use (no change)
- **GUI** - Definition moved to first mention (revised)
- **iGEM** - Defined on first use (revised)
- **CLI** - Defined on first use (no change)
- **API** - Defined on first use (no change)
- **URL** - Defined on first use (no change)
- **npm** - Defined on first use (revised)
- **PyPi** - Defined on first use (revised)
- **CRAN** - Defined on first use (revised)

15. Strengthen the Discussion: Connect the findings to existing literature in the discussion section to better contextualize the results.

Authors' response: The discussion is short on purpose. We feel that an overly long discussion section risks reiterating points already covered in the results section. As already stated, the SEGUID v2 does not really have competing solutions as no other prior checksum algorithm was able to handle a circular topology or distinguish between double and single stranded sequences in a non-ambiguous way.

16. Provide Practical Applications: Highlight how SEGUID v2 can be applied in real-world scenarios to make its significance clearer.

Authors' response: We use SEGUID as a tool in several ways. Many of the same sequence files are often shared among members in one of the research groups involved in this work and thus exist as copies on several computers. The checksums provide security against accidental errors and also an obvious way to communicate sequence updates. Here, the integration of the checksum in the DNA editor software helps. The second application is in the kind of teaching setting described in Figure 10 where correct results are calculated for correct answers to student exercise problems. The publication of this manuscript will hopefully help to widen the adoption of the checksum to other areas.

Reviewer 2

The authors are advised to carefully revise and resubmit the manuscript accordingly. The authors also also advised to provide the proper details of SHA-1 used in the paper. All the figures must be redraw again with a good quality.

Authors' response: We provide a reference for the widely used SHA-1: FIPS PUB 180-4 (2015). Secure hash standard (SHS). Federal Information Processing Standards Publication (FIPS PUB 180-4). This document describes the SHA-1 function in detail.

Figures 1-6, 9-10 are all *vector* graphics and are compiled into the PDF as such. In other words, they are provided in the highest resolution possible; it is possible to zoom in on the figures to any depth. If the critique is about accessibility such as low contrasts, please let us know, and we will revisit these figures.

Figures 7-8 are *bitmap* graphics. Figure 7a was a PNG of resolution 1245x609 pixels and Figure 7b a PNG of resolution 512x240 pixels (no change). We have updated Figure 7b to reflect the latest version of the ApE interface and such that it has resolution 902x479 pixels. The journal guidelines specifies that figures with multiple panels be meged into one figure. The Figures 7a and b were merged into a PNG of resolution 2400x600 pixels.

Figure 8 is a PNG of resolution 1949x997 pixels (no change).

If this is insufficient, please clarify which figures are not of acceptable quality and why.

Reviewer 3

"For example, the two proteins with clashing CRC-64 checksums (Figure 1) have distinct SEGUID checksums." I do not find this statement surprising or a good example of why SEGUID is superior.

Authors' response: We chose the CRC-64 checksum to illustrate the risk of checksum collisions. Since UniProt used this originally(*), and UniParc provides such checksums, CRC-64 serves as a real-world example of this problem. We also use CRC-64 as a segue for introducing the stronger SHA algorithms. We try to communicate that, although all checksum algorithms are subject to clashes, which we stated in our manuscript, the SHA-1 algorithm produces checksums for which the risk of a clash occurring unprovoked is so infinitesimally small as to be of no practical consequence. This is obviously not the case for the CRC-64 checksum.

(*) While revisiting this passage, we realized that our claim about UniProt was misleading. Although UniProt and UniParc still provides CRC-64 checksums, they are officially using MD5 checksums.

The authors have done a great job at providing multiple implementations in different programming languages, but I suspect a method like this will not be widely adopted by the community until it is incorporated into large databases such as UniProt. The authors should perhaps include this in the discussion.

Authors' response: We added "Since the checksum is already implemented in the popular DNA editor ApE, we believe this will encourage wider adoption by both researchers and web-facing applications." at the end to the end of the discussion to address this point.
