# Supplementary material for: SEGUID v2: Extending SEGUID checksums for circular, linear, single- and double-stranded biological sequences
Source: PLoS One. 2026 Aug 7;21(8):e0346164. doi: 10.1371/journal.pone.0346164 (PMC13450835; doi:10.1371/journal.pone.0346164)
Supplement: Supplementary file 3 [file pone.0346164.s004.pdf]

PLOS ONE

SEGUID v2: Extending SEGUID checksums for circular, linear, single- and double-stranded biological sequences

--Manuscript Draft--

**Manuscript Number:**	PONE-D-24-51050R1
**Article Type:**	Research Article
**Full Title:**	SEGUID v2: Extending SEGUID checksums for circular, linear, single- and double-stranded biological sequences
**Short Title:**	SEGUID v2: Unique checksums for circular, linear, single- and double-stranded sequences
**Corresponding Author:**	Henrik Bengtsson<br>University of California San Francisco<br>San Francisco, CA UNITED STATES OF AMERICA
**Keywords:**	checksum; Hash; DNA; RNA; protein; SHA-1; Base64url; SEGUID
**Abstract:**	Background: Synthetic biology involves combining different DNA fragments, each containing functional biological parts, to address specific problems. Fundamental gene-function research often requires cloning and propagating DNA fragments, such as those from the iGEM Parts Registry or Addgene, typically distributed as circular plasmids. Addgene's repository alone offers around 150,000 plasmids.<br>To ensure data integrity, cryptographic checksums can be calculated for the sequences. Each sequence has a unique checksum, making checksums useful for validation and quick lookups of associated annotations. For example, the SEGUID checksum, uniquely identifies protein sequences with a 27-character string.<br>Objectives: The original SEGUID, while effective for protein sequences and single-stranded DNA (ssDNA), is not suitable for circular DNA since there is no natural starting position nor for double-stranded DNA (dsDNA) since two separate sequences are present. Challenges include how to uniquely represent linear dsDNA, circular ssDNA, and circular dsDNA. To meet these needs, we propose SEGUID v2, which extends the original SEGUID to handle additional types of sequences.<br>Conclusions: SEGUID v2 produces orientation and rotation invariant checksums for single-stranded, double-stranded, possibly staggered, linear, and circular DNA and RNA sequences. Customizable alphabets allow for other types of sequences. In contrast to the original SEGUID, which uses Base64, SEGUID v2 uses Base64url to encode the SHA-1 hash. This ensures SEGUID v2 checksums can be used as-is in filenames, regardless of platform, and in URLs, with minimal friction.<br>Availability: SEGUID v2 is readily available for major programming languages, distributed under the MIT license. JavaScript package 'seguid' is available on npm, Python package 'seguid' on PyPi, R package 'seguid' on CRAN, and a Tcl script on GitHub. These tools, along with documentation, examples, and an online 'SEGUID Calculator', can be found at https://www.seguid.org.
**Order of Authors:**	Humberto Pereira
	Paulo César Silva
	M. Wayne Davis
	Louis Abraham
	György Babnigg
	Henrik Bengtsson
	Björn Johansson
**Response to Reviewers:**	Please see 'Respond_to_Reviewers.pdf`. Thank you.
**Additional Information:**	
**Question**	**Response**
**Financial Disclosure**	Yes

Powered by Editorial Manager® and ProduXion Manager® from Aries Systems Corporation

Enter a financial disclosure statement that describes the sources of funding for the work included in this submission. Review the submission guidelines for detailed requirements. View published research articles from *PLOS ONE* for specific examples.<br><br>This statement is required for submission and **will appear in the published article** if the submission is accepted. Please make sure it is accurate.<br><br>**Funded studies**<br>Enter a statement with the following details:<br>• Initials of the authors who received each award<br>• Grant numbers awarded to each author<br>• The full name of each funder<br>• URL of each funder website<br>• Did the sponsors or funders play any role in the study design, data collection and analysis, decision to publish, or preparation of the manuscript?<br><br>Did you receive funding for this work?	
Please add funding details.<br>as follow-up to "**Financial Disclosure**<br><br>Enter a financial disclosure statement that describes the sources of funding for the work included in this submission. Review the submission guidelines for detailed requirements. View published research articles from *PLOS ONE* for specific examples.<br><br>This statement is required for submission and **will appear in the published article** if the submission is accepted. Please make sure it is accurate.	BJ was funded by national funds via Fundação para a Ciência e Tecnologia Portugal (FCT; https://www.fct.pt/) I.P. through project FatVal PTDC/EAM-AMB/032506/2017, and by the European Regional Development Fund (ERDF; https://ec.europa.eu/regional_policy/funding/erdf_en) through the COMPETE2020 – Programa Operacional Competitividade e Internacionalizacão (POCI). The Center of Molecular and Environmental Biology Engineering (CBMA) was supported by the strategic program UIDB/04050/2020, funded by national funds via FCT I.P.<br><br>HB was funded through the UCSF Computational Biology Core (CBI) that is supported by a National Cancer Institute (NCI; https://www.cancer.gov/) Cancer Center Support Grant 5P30CA082103.<br><br>MWD was supported by NIH/NIGMS (https://www.nigms.nih.gov/) grant 1R01GM146005 (``Genome Engineering in the nematode C. elegans'').<br><br>GB was supported through Argonne National Laboratory managed by UChicago Argonne, LLC for DOE (https://www.energy.gov/) undercontract number

**Funded studies**<br>Enter a statement with the following details:<br>• Initials of the authors who received each award<br>• Grant numbers awarded to each author<br>• The full name of each funder<br>• URL of each funder website<br>• Did the sponsors or funders play any role in the study design, data collection and analysis, decision to publish, or preparation of the manuscript?<br><br>Did you receive funding for this work?"	DE-AC02-06CH11357. This program is supported by the U.S. Department of Energy, Office of Science, through the Biomolecular Characterization and Sciences Program, Office of Biological and Environmental Research, under FWP 39156.<br><br>None of the sponsors or funders played a role in the study design, data collection and analysis, decision to publish, or preparation of the manuscript.
Please select the country of your main research funder (please select carefully as in some cases this is used in fee calculation).<br>as follow-up to "**Financial Disclosure**<br><br>Enter a financial disclosure statement that describes the sources of funding for the work included in this submission. Review the submission guidelines for detailed requirements. View published research articles from *PLOS ONE* for specific examples.<br><br>This statement is required for submission and **will appear in the published article** if the submission is accepted. Please make sure it is accurate.<br><br>**Funded studies**<br>Enter a statement with the following details:<br>• Initials of the authors who received each award<br>• Grant numbers awarded to each author<br>• The full name of each funder<br>• URL of each funder website<br>• Did the sponsors or funders play any role in the study design, data collection and analysis, decision to publish, or preparation of the manuscript?<br><br>Did you receive funding for this work?"	UNITED STATES - US
**Competing Interests**	The authors have declared that no competing interests exist.

Use the instructions below to enter a competing interest statement for this submission. On behalf of all authors, disclose any competing interests that could be perceived to bias this work—acknowledging all financial support and any other relevant financial or non-financial competing interests.<br><br>This statement is required for submission and **will appear in the published article** if the submission is accepted. Please make sure it is accurate and that any funding sources listed in your Funding Information later in the submission form are also declared in your Financial Disclosure statement.<br><br>View published research articles from *PLOS ONE* for specific examples.<br><br>**NO authors have competing interests**<br><br>Enter: *The authors have declared that no competing interests exist.*<br><br>**Authors with competing interests**<br><br>Enter competing interest details beginning with this statement:<br><br>*I have read the journal's policy and the authors of this manuscript have the following competing interests: [insert competing interests here]*<br><br>* typeset	
**Ethics Statement**<br><br>Enter an ethics statement for this submission. This statement is required if the study involved:<br><br>• Human participants<br>• Human specimens or tissue<br>• Vertebrate animals or cephalopods<br>• Vertebrate embryos or tissues	N/A

• Field research<br><br>Write "N/A" if the submission does not require an ethics statement.<br><br>General guidance is provided below. Consult the submission guidelines for detailed instructions. **Make sure that all information entered here is included in the Methods section of the manuscript.**	

**Format for specific study types**<br><br>**Human Subject Research (involving human participants and/or tissue)**<br>• Give the name of the institutional review board or ethics committee that approved the study<br>• Include the approval number and/or a statement indicating approval of this research<br>• Indicate the form of consent obtained (written/oral) or the reason that consent was not obtained (e.g. the data were analyzed anonymously)<br><br>**Animal Research (involving vertebrate animals, embryos or tissues)**<br>• Provide the name of the Institutional Animal Care and Use Committee (IACUC) or other relevant ethics board that reviewed the study protocol, and indicate whether they approved this research or granted a formal waiver of ethical approval<br>• Include an approval number if one was obtained<br>• If the study involved *non-human primates*, add *additional details* about animal welfare and steps taken to ameliorate suffering<br>• If anesthesia, euthanasia, or any kind of animal sacrifice is part of the study, include briefly which substances and/or methods were applied<br><br>**Field Research**<br><br>Include the following details if this study involves the collection of plant, animal, or other materials from a natural setting:<br>• Field permit number<br>• Name of the institution or relevant body that granted permission	
**Data Availability**<br><br>Authors are required to make all data underlying the findings described fully available, without restriction, and from the time of publication. PLOS allows rare exceptions to address legal and ethical concerns. See the PLOS Data Policy and FAQ for detailed information.	Yes - all data are fully available without restriction

A Data Availability Statement describing where the data can be found is required at submission. Your answers to this question constitute the Data Availability Statement and **will be published in the article**, if accepted.<br><br>**Important:** Stating 'data available on request from the author' is not sufficient. If your data are only available upon request, select 'No' for the first question and explain your exceptional situation in the text box.<br><br>Do the authors confirm that all data underlying the findings described in their manuscript are fully available without restriction?	
**Describe where the data may be found in full sentences. If you are copying our sample text, replace any instances of XXX with the appropriate details.**<br><br>• If the data are **held or will be held in a public repository**, include URLs, accession numbers or DOIs. If this information will only be available after acceptance, indicate this by ticking the box below. For example: *All XXX files are available from the XXX database (accession number(s) XXX, XXX.*).<br>• If the data are all contained **within the manuscript and/or Supporting Information files**, enter the following: *All relevant data are within the manuscript and its Supporting Information files.*<br>• If neither of these applies but you are able to provide **details of access elsewhere**, with or without limitations, please do so. For example:<br><br>*Data cannot be shared publicly because of [XXX]. Data are available from the XXX Institutional Data Access / Ethics Committee (contact via XXX) for researchers who meet the criteria for access to confidential data.*<br><br>*The data underlying the results presented in the study are available from (include the name of the third party*	All relevant data are within the manuscript and its Supporting Information files.

and contact information or URL).<br>• This text is appropriate if the data are owned by a third party and authors do not have permission to share the data.<br><br>* typeset	
Additional data availability information:	

Powered by Editorial Manager® and ProduXion Manager® from Aries Systems Corporation

Revised Manuscript

Click here to access/download;Manuscript;Pereira_etal_2024-SEGUIDv2.pdf

SEGUID v2: Extending SEGUID checksums for circular, linear, single- and double-stranded biological sequences

Humberto Pereira[1], Paulo César Silva[1], M. Wayne Davis[2], Louis Abraham[3], György Babnigg[4], Henrik Bengtsson[5,6◕*], Björn Johansson[1◕]

1 Center of Molecular and Environmental Biology Engineering, University of Minho, Campus de Gualtar, Braga, 4710-057, Portugal
2 Howard Hughes Medical Institute and School of Biological Sciences, University of Utah, Salt Lake City, UT, USA
3 PRISM, Université Paris 1, Panthéon–Sorbonne, Paris, France
4 Biosciences Division, Argonne National Laboratory, Argonne, IL, USA
5 Department of Epidemiology and Biostatistics, University of California, San Francisco, CA, USA
6 Helen Diller Family Comprehensive Cancer Center, University of California, San Francisco, CA, USA

◕These authors contributed equally to this work.
* corresponding author henrik.bengtsson@ucsf.edu

Abstract

Background: Synthetic biology involves combining different DNA fragments, each containing functional biological parts, to address specific problems. Fundamental gene-function research often requires cloning and propagating DNA fragments, such as those from the iGEM Parts Registry or Addgene, typically distributed as circular plasmids. Addgene's repository alone offers around 150,000 plasmids.

To ensure data integrity, cryptographic checksums can be calculated for the sequences. Each sequence has a unique checksum, making checksums useful for validation and quick lookups of associated annotations. For example, the SEGUID checksum, uniquely identifies protein sequences with a 27-character string.

Objectives: The original SEGUID, while effective for protein sequences and single-stranded DNA (ssDNA), is not suitable for circular DNA since there is no natural starting position nor for double-stranded DNA (dsDNA) since two separate sequences are present. Challenges include how to uniquely represent linear dsDNA, circular ssDNA, and circular dsDNA. To meet these needs, we propose SEGUID v2, which extends the original SEGUID to handle additional types of sequences.

Conclusions: SEGUID v2 produces orientation and rotation invariant checksums for single-stranded, double-stranded, possibly staggered, linear, and circular DNA and RNA sequences. Customizable alphabets allow for other types of sequences. In contrast to the original SEGUID, which uses Base64, SEGUID v2 uses Base64url to encode the SHA-1 hash. This ensures SEGUID v2 checksums can be used as-is in filenames, regardless of platform, and in URLs, with minimal friction.

Availability: SEGUID v2 is readily available for major programming languages, distributed under the MIT license. JavaScript package **seguid** is available on npm, Python package **seguid** on PyPi, R package **seguid** on CRAN, and a Tcl script on GitHub. These tools, along with documentation, examples, and an online **SEGUID Calculator**, can be found at `https://www.seguid.org`.

Introduction

The post-genomic era has provided access to more biological sequence data than ever before. These sequence data provide an important resource for both fundamental research and synthetic biology, which aim to understand and harness biology to solve urgent societal problems, such as global warming or food security. Genetic parts contained on DNA fragments are distributed to and from culture collections and repositories, as well as within the research community to study genes and build new synthetic systems. These parts are almost always distributed as plasmids, where the DNA of interest is inserted into a circular double-stranded (ds) DNA plasmid providing replication and selection in a microorganism optimal for producing high-quality DNA, usually *E. coli*.

Each plasmid should have an associated sequence file that is distributed along with the physical clone. Although repositories such as Addgene [1, 2] and the iGEM (International Genetically Engineered Machine) Parts collection provide standards, the transmission of biological material and associated information is still very much an ad-hoc procedure.

Data integrity is of great importance since it directly affects the reproducibility of science that relied on the original genetic parts. Checksums are widely used for verifying that information is preserved through space and time, and has also been applied to safe-guard biological sequences. A checksum is a short string of characters that is calculated from, and typically stored with, arbitrarily sized data. By transmitting the checksum together with the data, a receiver can verify correctness of the data.

Fig 1. Two protein sequences with colliding CRC-64 checksums. The two protein sequences with GenBank accession number *AAF20481.1*$_{\text{GB}}$ and *AAF20475.1*$_{\text{GB}}$ have both 110 amino acids and differ at two positions (91 and 99). Despite this, they share the same UniProt **CRC-64** checksum. The **SHA-1**-based SEGUID v2 checksums differ.

Several existing checksum algorithms are relevant in bioinformatics. One of the first used was the cyclic redundancy check (**CRC**) [3], which is a computationally inexpensive, error-detecting algorithm originally designed for network and storage devices. The UniProt database originally used a 64-bit **CRC** (**CRC-64**) checksum[1]. This is the reason why the UniProt Archive (UniParc) still provides **CRC-64**[2]. A disadvantage of the **CRC-64** is that collisions have been observed for biological data. For example, the two protein sequences with GenBank accession numbers *AAF20475.1*$_{\text{GB}}$[3] and *AAF20481.1*$_{\text{GB}}$[4], have the same **CRC-64** hexadecimal checksum, despite differing in two of the 110 amino acids (Fig 1).

While collisions are unavoidable for all functions producing finite-length checksums, longer checksums are less likely to generate collisions. However, long checksums are less readable and also more expensive to generate, store, and compare. The SEquence Globally Unique IDentifier (SEGUID) checksum [4] was introduced in order to provide a stable, unifying key for the same sequence in different databases facilitating linking protein sequences across databases. SEGUID is based on the **SHA-1** (Secure Hash Algorithm 1) algorithm [5], which produces a 160-bit hash, and is therefore less sensitive to collisions compared with the 64-bit **CRC-64** hash. With **SHA-1**,

[1] https://www.uniprot.org/help/checksum
[2] https://www.uniprot.org/help/uniparc
[3] UniParc accession number *UPI0000117F35*$_{\text{UP}}$
[4] UniParc accession number *UPI0000117F39*$_{\text{UP}}$

sequence collisions are very unlikely. For example, the two proteins with clashing **CRC-64** checksums (Fig 1) have distinct SEGUID checksums.

More importantly, no current checksum algorithm is directly useful for DNA, since the topology of DNA is most often different from that of proteins. Double-stranded DNA (dsDNA) consists of two complementary, anti-parallel strands. Usually, only one is stored for DNA, as the second strand is the complement of the stored sequence. However, both strands are equally valid representations of the DNA molecule, but give different checksum values, except for the special case where the sequence is a palindrome and the strands therefore identical.

DNA may be circular, e.g. circular single-stranded DNA (ssDNA) or circular dsDNA. For these cases, there are multiple representations for the same circular sequence, and there is no well-defined canonical representation for which the checksum should be calculated.

In this article, we propose SEGUID v2, which is an extension to the original SEGUID, that produces checksums for linear, circular, single- and double-stranded sequences of different types, including DNA, RNA, and amino-acid sequences in order to integrate synthetic biology resources and identify redundancies in existing databases.

We believe that a stable checksum that describes any biological sequence, regardless of topology or strandedness, would streamline the exchange of information and biological materials within the synthetic biology community.

Methods

The problem of defining a checksum for sequences of different kinds, comprise three challenges: (i) identify a unique representation among one or more equivalent representations, and convert it to a linear sequence representation, (ii) choose a hash function to distill the unique representation to a large-enough bit sequence, and (iii) compactly encode the bit sequence to a checksum using a text-friendly encoding alphabet.

Below, we explain how we define each of these steps for the proposed SEGUID v2 checksum, and what objectives we target when there are multiple options available.

Unique representation of sequence data

Linear single-stranded sequences

A *linear single-stranded sequence* is a single, directional sequence with one or more symbols, where *directional* means it has a unique start and end ("left to right"). The sequence may be amino acids, DNA, RNA, or any sequence of symbols from a well-defined, case-sensitive alphabet. Examples of DNA sequences are `C`, `AT`, and `GATTACA`. Examples of RNA sequences are `C`, `AU`, and `AGA`. Examples of amino-acid sequences ("proteins") are `A`, `QSA`, and `QSALTQPASV`.

A linear single-stranded sequence is always unique, which makes it natural to use the sequence as-is to calculate the checksum. This case was already covered by the original SEGUID algorithm.

Linear double-stranded sequences

A *linear double-stranded sequences* comprise two sequences that share a region where the two sequences are complementary to each other. For double-stranded DNA, the 5′→3′ "upper" strand is often referred to as the "Watson" strand and the complementary "lower" as the "Crick" strand. We use the Watson and Crick strands

separated by the symbol ⟷, where both Watson and Crick are $5'\to3'$ sequences, to represent a double-stranded sequence. Using this notation, the double-stranded sequence `GATTACA⟷TGTAATC` reflects the graphical representation in Fig 2.

Two types of such sequences must be considered - (i) blunt-ended, and (ii) staggered, linear double-stranded sequences - which are discussed next.

Fig 2. The SEGUID v2 steps for identifying the canonical representation of a blunt-ended linear, double-stranded DNA sequence. The input sequence `GATTACA⟷TGTAATC` (left and top middle) has a dual, complementary representation `TGTAATC⟷GATTACA` (bottom middle). The lexicographically smallest of these two is `GATTACA⟷TGTAATC` (right). It is from this linear representation that the SEGUID v2 checksum is calculated. Legend: Following IUPAC-IUB Commission on Biochemical Nomenclature (1970) [6], we choose to rotate the symbols on the complementary strand in the figure such that the left-to-right, $3'\to5'$ representation holds when rotating the figure upside-down.

Blunt-ended, linear double-stranded sequences: In its basic form, the two sequences are of the same length and fully overlapping, i.e. the shared region spans their full length. We refer to this type of sequences as *blunt-ended* (short blunt), or non-staggered, double-stranded sequences. For example, `GATTACA⟷TGTAATC` is a blunt, double-stranded sequence with complementary strands `GATTACA` and `TGTAATC`. Importantly, `GATTACA⟷TGTAATC` and `TGTAATC⟷GATTACA` are dual representations of the same linear double-stranded DNA sequence (Fig 2). In other words, *without further constraints*, there is no unique representation of it. To address this *uniqueness problem*, we define the unique representation of the two possibilities as the one that is lexicographically smaller[5] than the other. This is possible for all well-defined alphabets. For example, consider the DNA alphabet `A`, `C`, `G`, `T`, ordered such that `A` < `C` < `G` < `T`. This allows us to compare any two sequences lexicographically, e.g. `AT` < `TA`. For example, in the above example, we have that `GATTACA⟷TGTAATC` < `TGTAATC⟷GATTACA`, and any checksums will be calculated based on the first of these two representations (Fig 2).

Fig 3. The SEGUID v2 steps for identifying the canonical representation of a staggered, linear, double-stranded DNA sequence. The input sequence `-ATTACA⟷--TAATC` (left and top middle) has a dual, complementary representation `--TAATC⟷-ATTACA` (bottom middle). Because the minus symbol (`-`) is lexicographically smaller than the other symbols, the smallest of these two linear representations is `--TTACA⟷-GTAATC` (right). It is from this linear representation that the SEGUID v2 checksum is calculated.

Staggered, linear double-stranded sequences: Another form of double-stranded sequences is where the two sequences do not fully overlap. We refer to this type of sequences as *staggered* double-stranded sequences, e.g. `-ATTACA⟷TGTAATC`, `--TTACA⟷TGTAATC`, and `-ATTACA⟷-GTAATC`. Here we use the minus symbol (`-`) to indicate that there is a symbol on the opposite strand but not on the current strand. As for blunt double-stranded sequences, staggered ones have two possible representation for the same sequence, e.g. `-GTAATC⟷--TTACA` and `--TTACA⟷-GTAATC` refer to the same sequence. As before, we define the unique representation to be the lexicographically smallest of the two, where we define the minus symbol (`-`) to be smaller than any other symbol, e.g. with the DNA alphabet we have that `-` < `A` < `C` < `G` < `T`. With this, we can identify `--TTACA⟷-GTAATC` as the minimal lexicographic

[5]Strictly speaking, "smaller or equal" is sufficient to get a unique representation.

representation, because --TTACA↩-GTAATC < -GTAATC↩--TTACA (Fig 3). It is from this canonical representation that the hash is calculated.

Circular single-stranded sequences

A *circular single-stranded sequence* is a single, directional sequence with one or more symbols without a beginning or end. The sequence may comprise of symbols from any well-defined alphabet. Any linear single-stranded sequence can be made a circular single-stranded sequences, by combining the two ends, and vice versa. Examples of circular DNA sequences are C↻, AT↻, TA↻, and GATTACA↻. Here we use the symbol ↻ to indicate that the two ends are combined. A naturally occurring circular, single-stranded DNA sequences is the 6,407-nucleotide M13 bacteriophage with sequence AACGCTACTA...GGATGTT↻ (*NC_003287.2*$_{\text{GB}}$ and *V00604.2*$_{\text{GB}}$). Circular RNA (circRNA) are naturally occurring circular RNA formed from mRNA through a process called back-splicing [7], where a downstream splice donor site is joined to an upstream splice acceptor site. The *MW729338.1*$_{\text{GB}}$ represents an 840-nucleotide circRNA from *Culex pipiens* (northern house mosquito). Examples of circular proteins are A↻, QSA↻, and QSALTQPASV↻. A naturally occurring circular single-stranded amino-acid sequence is the 21 amino-acid long *E. coli* peptide Microcin J25 (*AAD28494.1*$_{\text{GB}}$ at positions 38–58) with sequence GGAGHVPEYFVGIGTPISFYG↻. This circular peptide is also referred to as VGIGTPISFYGGGAGHVPEYF↻, e.g. Blond et al. (1999) [8] and Trabi and Craik (2002) [9].

Fig 4. The SEGUID v2 steps for identifying the canonical representation of a circular, single-stranded DNA sequence. The input sequence GATTACA↻ (left) is one of several equivalent, non-normalized representations of the same circular sequence. Among all seven possible rotations, there is a minimal rotation, which for this sequence is ACAGATT↻ (middle). The linear representation of the minimal rotation is ACAGATT (right). It is from this linear representation that the SEGUID v2 checksum is calculated.

Because there is no beginning and end of a circular sequence, there is also no unique representation of a circular sequence *without further constraints*. both AT↻ and TA↻ represents the same circular sequence. Similarly, GATTACA↻, ATTACAG↻, TTACAGA↻, TACAGAT↻, ACAGATT↻, CAGATTA↻, and AGATTAC↻ are all the same circular sequence. In general, there are n different representations of the same circular single-stranded sequence of length n.

The non-uniqueness of these representations complicates how to store a circular sequence in databases, how to communicate it electronically, and how to present it in text. Similarly, if we naively would calculate the original SEGUID checksum for the above representations, we would get different checksums despite them representing the same circular sequence.

To address the above *uniqueness problem*, we need to identify a single unique representation among all possible representations part of the same *equivalent class*. Analogously to how we addressed the uniqueness problem for double-stranded sequences, a natural choice is to define the unique representation to be *the sequence with a rotation that has the smallest lexicographical value*. For example, consider the above circular sequence GATTACA↻ with seven equivalent representations. We can order the seven possible rotations as ACAGATT < AGATTAC < ATTACAG < CAGATTA < GATTACA < TACAGAT < TTACAGA. The *minimal lexicographical rotation* of GATTACA↻ is therefore ACAGATT (Fig 4). The minimal rotation can be found in linear time [10].

Circular double-stranded sequences

A *circular double-stranded sequences* is similar to a *circular single-stranded sequences*, but with a second complementary strand. Examples of circular double-stranded DNA sequences are `C`↩`G`⟳, `AT`↩`AT`⟳, and `TATGCCAA`↩`TTGGCATA`⟳. A real-world example is the 2,686-basepair circular double-stranded DNA pUC19 cloning vector `TCGC...CGTC`↩`GACG...GCGA`⟳ (*M77789.2*$_{\text{GB}}$ and *L09137.2*$_{\text{GB}}$).

Fig 5. The SEGUID v2 steps for identifying the canonical representation of a circular, double-stranded DNA sequence. The input sequence `TATGCCAA`↩`TTGGCATA`⟳ (left, and top left) is one of several equivalent, non-normalized representations of the same circular sequence. This double-stranded sequence has the dual complementary representation `TTGGCATA`↩`TATGCCAA`⟳ (bottom left). The minimal rotations for these dual representations are `AATATGCC`↩`GGCATATT`⟳ (top middle) and `ATATTGGC`↩`GCCAATAT`⟳ (bottom middle). The smallest of the two linear minimal-rotated representations (top right and bottom right), is `AATATGCC`↩`GGCATATT` (right). It is from this linear representation that the SEGUID v2 checksum is calculated.

Similarly to the single-stranded cases, there are multiple representations for circular double-stranded sequences. For example, the eight rotational variants `TATGCCAA`↩`TTGGCATA`⟳, `ATGCCAAT`↩`ATTGGCAT`⟳, `TGCCAATA`↩`TATTGGCA`⟳, . . ., `ATATGCCA`↩`TGGCATAT`⟳ represent the same eight-basepair circular dsDNA, but so does the eight complementary counterparts `TTGGCATA`↩`TATGCCAA`⟳, `ATTGGCAT`↩`ATGCCAAT`⟳, `TATTGGCA`↩`TGCCAATA`⟳, . . ., `TGGCATAT`↩`ATATGCCA`⟳. In general, there are $2 \cdot n$ different representations of the same circular double-stranded sequence of length n. We define the unique representation as *the smallest of the minimal lexicographical rotations of the two strands*. For instance, `AATATGCC`↩`GGCATATT`⟳ is the minimal for one of original representation, and `ATATTGGC`↩`GCCAATAT`⟳ for the complementary duality. Between these two, `AATATGCC`↩`GGCATATT`⟳ is the lexicographically smallest, because `AATATGCC`↩`GGCATATT`⟳ < `ATATTGGC`↩`GCCAATAT`⟳. Thus, the canonical representation of the circular double-stranded sequence `TATGCCAA`↩`TTGGCATA`⟳ is `AATATGCC`↩`GGCATATT`⟳ (Fig 5). Note, it is sufficient to compare only `AATATGCC` < `ATATTGGC` to identify `AATATGCC`↩`GGCATATT`⟳ to be the lexicographically minimal representation. This is true also for non-bijective complementary alphabets.

Hash function (SHA-1)

SEGUID v2 supports linear and circular, single- and double-stranded sequences. As shown above, circular sequences are transformed to unique, linear representations based on minimal rotations and lexicographic ordering. Thus, regardless of input sequence, the hash is always calculated on a linear representation. To apply the hash function to these representations, we need to coerce them to single strings[6]. There are two types of linear representations we have to consider; the single-stranded one and the double-stranded one. A linear *single*-stranded sequence can be coerced to a string as-is, e.g. the sequence `GATTACA` becomes the string `"GATTACA"`. For a linear *double*-stranded sequence, we chose to coerce it to a string by concatenating the 5′→3′ Watson sequence and the 5′→3′ Crick sequences with a semicolon (`;`). For example, the blunt, for linear dsDNA sequence `GATTACA`↩`TGTAATC`, the hashed string is

[6]Technically, hash functions operates on raw bytes. To calculate a hash for a string, we ensure to use ASCII strings such that each characters uniquely maps to an integer byte.

`"GATTACA;TGTAATC"`. Similarly, for the staggered, linear dsDNA sequence `--TTACA↩-GTAATC` the hashed string is `"--TTACA;-GTAATC"`.

The original SEGUID method uses **SHA-1** to generate a 160-bit hash from the input sequence. As argued in Babnigg and Giometti (2006) [4], **SHA-1** is less sensitive to collisions compared to **CRC-64** and sequence checksum collisions are very unlikely when using **SHA-1**. Another, popular hash function is the Message Digest Algorithm 5 (**MD5**) [11], which produces a 128-bit hash. The UniProt database uses the **MD5** checksum. Although we are not aware of any collisions for biological data, it has been shown [12] that **MD5** collisions can be created artificially. Although we have not yet observed a **SHA-1** collision, one could argue that we should "upgrade" to a **SHA-2** algorithm to further lower the risk for collisions. On the other hand, that comes with a cost of producing longer checksums. For example, the popular and widely available **SHA-256** algorithm produces a 256-bit long hash, which is 60% longer than a **SHA-1** hash, which in turn would lengthen the final checksum the same amount. There are downsides to having long checksums. For instance, they may be too long to be used for metadata headers in some sequence file formats, they risk being line-wrapped in communications such as email threads, and they add to the storage size for very-large lookup tables. Moreover, some file systems have a limit on how long a canonical pathname may be, e.g. on Microsoft Windows the default pathname-length limit is 256 characters. A very long checksums in a directory or file name, in an already deeply nested directory structure, may exceed this limit. An alternative to **SHA-256** is **SHA-224**, which results in "only" a 40% longer checksum and might therefore be a hash-algorithm candidate. Unfortunately, implementations of **SHA-224** are less common across programming languages than **SHA-1**, **SHA-256**, and **SHA-512**. Yet another alternative is **SHA-512/224**, which produces a 224-bit truncated hash based on the **SHA-512** algorithm [5]. The SHA specification support **SHA-512/t** for other values of **t**, e.g. **SHA-512/160** would produce a hash of the same length as **SHA-1**. Readily available implementations of these truncated alternatives are even less common than **SHA-224**. An alternative approach to **SHA-512/t** was proposed by Hart and Prlić (2020) [13], where they discard the last 320 bits of the 512-bit **SHA-512** hash to produce a 192-bit hash. We contemplated this approach, but we decided against it as we do not know if it is statistically sound or valid for our needs.

Lastly, Stevens et al. (2017) [14] and Leurent and Peyrin (2020) [15] demonstrated that it is feasible to artificially construct **SHA-1** clashes on modern computers. Because of this, **SHA-1** is no longer considered safe from a cryptography point of view. For instance, the U.S. National Institute of Standards and Technology (NIST) "*recommends that federal agencies transition away from SHA-1 for all applications as soon as possible. Federal agencies should use SHA-2 or SHA-3 as an alternative to SHA-1*" [16]. The **SHA-2** and **SHA-3** family of hash functions produce longer hashes, making it harder to construct collisions. That said, sequence checksums are used for efficient storage, communication, and lookup of sequences - not to secure them cryptographically. Arguing that the checksum of a full sequence can guarantee that the full sequence has not be compromised is problematic and risky. To achieve that, cryptographical protocols based on, for instance, public-and-private keys should be used, which is a problem beyond sequence checksums. We find no reason to incorporate such features in SEGUID v2, because there already exist well-established, readily available protocols for encrypted communication and storage.

For these reasons, we choose to use **SHA-1** also for SEGUID v2.

Encoding (Base64url)

A hash, by itself, is sufficient to uniquely identify a sequence. However, hashes, such as **SHA-1**, are very large integers, larger than can easily be represented in common

programming languages, which makes them complicated to coerce as-is to and from textual representations. Encoding is a technique to turn an internal binary representation to a string, preferably in a compact way. Different encoding algorithms have different "compression" ratios (Table 1).

Description	Value	Length
**SHA-1** (binary)	$10011010110110\ldots0010100100_2$	160 bits
**SHA-1**+Base16	`9adaef6edbb0afafcca01c6fb66e01129bfe8ca4`	40 symbols
**SHA-1**+Base64	`mtrvbtuwr6/MoBxvtm4BEpv+jKQ=`	28 symbols
**SHA-1**+Base64url	`mtrvbtuwr6_MoBxvtm4BEpv-jKQ=`	28 symbols
SEGUID v1	`mtrvbtuwr6/MoBxvtm4BEpv+jKQ`	27 symbols
SEGUID v2	`mtrvbtuwr6_MoBxvtm4BEpv-jKQ`	27 symbols

Table 1. Examples of different encodings of a SHA-1 hash. The **SHA-1** hash was generated based on the sequence string `ACAGATT`. The **Base64** specification requires that the output comprise a multiple of four symbols, and if not, the output should be padded with equal signs (`=`) at the end. Because of this, and a **Base64** encoding of a **SHA-1** hash will always have a single, non-informative padding symbol at the end. This padding symbol is excluded in the SEGUID checksums.

For instance, the **SHA-1** hash, which is a 160-bit integer, or equivalently a 20-byte integer, could be encoded to a string of length 160 comprising symbols zero (`0`) and one (`1`). Assuming an 8-bit ASCII string, this encoding is 800% larger than the internal 20 bytes. That would be an inefficient way to represent the hash. A more compact, and commonly used encoding is the hexadecimal representation, which comprise an 16-character alphabet (`0-9` and `a-f`). In this representation, a **SHA-1** hash is of length 40 characters; one for every four ($\log_2 16$) bits, or two for each byte. To achieve a more compact checksum, a larger set of symbols is needed. The **Base64** encoding [17] uses a 64-character alphabet (`A-Z`, `a-z`, `0-9`, `+`, and `/`). With this, each character can encode six ($\log_2 64$) bits, i.e. a 160 bits can be represented by $\lceil 160/6 \rceil = 27$ characters.[7]

The original SEGUID method uses **Base64**, modulo the mandatory, non-informative padding symbol, to encode the **SHA-1** hash, resulting in a 27-character checksum. This was chosen to get a compact representation of the hash, while still being text-friendly. One problem with a **Base64**-based checksum is that it cannot be used as part of a filename, because of the forward slash (`/`), which is used to represent directories on most file systems. For similar reason, it cannot be used as-is as part of a Uniform Resource Locator (URL), where symbols `/` and `+` have special meaning. In order to use a SEGUID checksum in a URL, these symbols need to be re-encoded. To address these needs, a filename- and URL-safe variant of **Base64** is the **Base64url** encoding [17]. It substitutes forward slashes (`/`) and plus symbols (`+`) with underscores (`_`) and minus symbols (`-`). The **Base64url** algorithm is linear ($O(n)$) in the number of bits, which is always 160 bits when using **SHA-1**.

In contrast to the original SEGUID, for SEGUID v2, we choose to use the **Base64url** encoding. For example, the linear single-stranded DNA sequence `ACAGATT` yields the SEGUID v1 checksum `mtrvbtuwr6/MoBxvtm4BEpv+jKQ`, while the SEGUID v2 checksum is `mtrvbtuwr6_MoBxvtm4BEpv-jKQ` (Table 1).

SEGUID v2 checksums have a prefix

In order to preserve what type of information is encoded in the checksum, SEGUID v2 checksums are by default prefixed with either `lsseguid`, `csseguid`, `ldseguid`, or

[7] Technically, **Base64** encoding requires a multiple of four symbols. To meet this requirement, the 27-character string representing a **SHA-1** hash should be padded with a special symbol (`=`) at the very end.

`cdseguid`, followed by an equal sign (`=`). For example, `lsseguid=IQiZThf2zKn_I1KtqStlEdsHYDQ` indicates a SEGUID v2 checksum for a linear single-strand sequence.

The prefix helps to distinguish SEGUID v2 checksums from other types of 27-character checksum strings. It also conveys type of sequence the checksum represents. We have found that the latter helps users to detects mistakes sooner in case they selected the incorrect sequence type for SEGUID v2.

Another advantage of the prefix is that it eliminates the risk of getting checksums with all digits, which some software tools might parse as numeric values. For example, Microsoft Excel and LibreOffice Calc will coerce such all-digit strings to numerics, but with loss of precision. If such a numeric value is coerced back to a string, it will comprise of the first 15-16 digits followed by all zeros. We observe the same problem with higher-level programming functions (e.g. in R and Python) that reads data from file risk reading all-digit columns as loss-of-precision numeric values or overflowed integer values.

SEGUID Short ID

For practical purposes, SEGUID v2 defines a *short form* of its checksum. Specifically, a *SEGUID Short ID* comprise the first six (6) characters of the checksum without the prefix. For example, the Short ID of the SEGUID v2 checksum `lsseguid=IQiZThf2zKn_I1KtqStlEdsHYDQ` is `IQiZTh`.

While we designed the SEGUID v2 checksum to be text-friendly, we designed the Short ID to be human-friendly. The *SEGUID Short ID* provides a compact way to reference a specific sequence. The short length makes it easy to communicate in both written and spoken form, and they allow for quick manual comparisons. It is also feasible to memorize and recognize, especially in projects that have a few sequences that are commonly used.

The risk for collisions among Short ID is still relatively small, especially for any given project working with a limited set of sequences. Theoretically, a six-character Short ID can encode $2^{36} = 1,073,741,824$ different sequences[8].

Supported sequence types and alphabets

The SEGUID v2 algorithm is designed to support DNA, RNA, and proteins, but also sequences with a custom alphabet (Table 2). Formally, the input sequence must not include symbols outside of the declared *case-sensitive* alphabet. By requiring case sensitivity, we allow for a richer set of custom alphabets, but we also avoid the risk of introducing non-deterministic behavior from, for instance, automatically coercing lower-case letters to upper-case ones. In addition, we limit alphabets to digits, and upper-, and lower-case letters. This is to assure a well-defined lexicographic ordering of symbols (per the 7-bit ASCII character set). Importantly, there are *reserved symbols* that must never be part of the alphabet. Specifically, the dash symbol (`-`), the semicolon (`;`), and the newline (`\n`) are reserved symbols.

Results

SEGUID v2 is designed to be robust against mistakes in order to prevent incorrect checksums from propagating in the scientific process. For instance, part of the requirements is that the implementation should validate that the correctness of the input

[8] The Short ID holds 6 symbols $\cdot \log_2 64$ bits/symbol $= 36$ bits (22.5%) of the original 160 bits of the **SHA-1** hash.

Alphabet	#Symbols	Complementary	Definition
DNA	4	Bijective	Nucleotides A↔T, C↔G.
Extended DNA	15	Bijective	DNA + K↔M, R↔Y, S↔S, W↔W, B↔V, D↔H, N↔N.
RNA	4	Bijective	Nucleotides A↔U, C↔G.
Extended RNA	15	Bijective	RNA + K↔M, R↔Y, S↔S, W↔W, B↔V, D↔H, N↔N.
Protein	20	No	Amino acids A, C, D, E, F, G, H, I, K, L, M, N, P, Q, R, S, T, V, W, Y, plus U, O.
Extended protein	26	No	Protein + B, J, Z, X.
Custom	1 − 60	Optional	Digits (0-9), upper-case letters (A-Z), lower-case letters (a-z).

Table 2. Alphabets supported by SEGUID v2. SEGUID v2 has built-in support for DNA, RNA, and proteins. These alphabets comprise upper-case letters only. IUPAC is a standard to represent DNA, RNA, and amino-acid sequences with an "extended" set of symbols [6, 18]. Custom alphabets, complementary or not, are also supported. The concept of complementarity does not apply to proteins. Proteins are effectively single-stranded sequences, meaning only the single-stranded SEGUID v2 methods apply to them. Amino-acid symbols J, O, and U are not part of the IUPAC standard.

sequence, e.g. all symbols are part of the alphabet and double-stranded sequences are correctly complementary. At the same time as being strict, SEGUID v2 is designed to be flexible such that it can support existing and future sequences not already anticipated. For example, in Viner et al. (2024) [19], the authors define an expanded epigenetic DNA alphabet (Table 3) that encodes also known modifications to cytosine (C). Using this alphabet, methylation motifs can be represented as, for instance, TTGmGCAA, AT2hAAAT, and TGCm1m1. Their complementary counterparts are TTGC1CAA, ATTT2hAT, and m1m1GCA. They also define an "extended" version of this alphabet, with additional complementary symbol pairs. Both of these alphabets can be used with SEGUID v2 by defining them as custom alphabets.

Fig 6. The SEGUID v2 algorithm on double-stranded sequence of DNA with uracil. The SEGUID v2 algorithm supports non-bijective complementary alphabets such as DNA with uracil (U). The input sequence UATUACA←→TGTAAUT (left and top middle) comprise basepairs A↔T and A↔U. It has a dual, complementary representation TGTAAUT←→UATUACA (bottom middle), which is also the lexicographically smallest (right).

Another example arise from the fact that uracil (U) may occur naturally in DNA, e.g. phage DNA from dUTPase, uracil-DNA glycosylase (*dut ung*) mutant hosts [20], as an apoptosis inducer during *Holometabola* development [21], and as a mean for somatic hypermutation resulting from U:G mismatch repair during vertebrate antibody development [22]. See Fig 6 for an illustration.

This also occurs in the USER^{TM} Uracil excision cloning protocol [23]. In these cases, adenine (A) may bind to either thymine (T) or uracil (U), which can be described by a *non-bijective* complementary alphabet (Table 3).

Alphabet	#Symbols	Complementary	Definition
Expanded epigenetic alphabet	12	Bijective	DNA + m↔1, h↔2, f↔3, c↔4.
Ambiguous expanded epigenetic alphabet	20	Bijective	Expanded epigenetic alphabet + w↔6, x↔7, y↔8, z↔9.
DNA with Uracil	5	Non-bijective	A↔T, A↔U, C↔G.

Table 3. Custom alphabets with SEGUID v2. (i) The expanded epigenetic alphabet, defined in Table 1 of Viner et al. (2024) [19], includes known modifications to cytosine (C) and symbols for each guanine (G) complementary to a modified nucleobase. (ii) The "ambiguous" version of this alphabet, defined in Table 2 of Viner et al. (2024) [19], addresses the case when the knowledge on some of nucleotides is incomplete. (iii) Uracil (U) may occur naturally in DNA, which in case adenine (A) is complementary to either thymine (T) or uracil (U). This can be represented by a non-bijective complementary alphabet.

Implementation and availability

Minimal, core software implementations

The SEGUID v2 algorithm, together with original SEGUID algorithm, is implemented and distributed as libre, open-source software under the MIT license. We provide small, self-contained implementations in JavaScript [24], Python [25], R [26], and Tcl [27] that have a minimal set of dependencies. For JavaScript, Python and R, we provide packages named **seguid**, which are available on npm (node package manager), PyPi (Python Package Index), and CRAN (Comprehensive R Archive Network), respectively. These packages implement a standardized application protocol interface (API), e.g. `csseguid("GATTACA")`. They also provide a command-line interface (CLI), e.g.

```
$ npx seguid --type=lsseguid <<< 'GATTACA'
lsseguid=tp2jzeCM2e3W4yxtrrx09CMKa_8

$ python -m seguid --type=cdseguid <<< 'TATGCCAA;TTGGCATA'
cdseguid=dUxN7YQyVInv3oDcvz8ByupL44A

$ Rscript -e seguid::seguid --type=csseguid <<< 'GATTACA'
csseguid=mtrvbtuwr6_MoBxvtm4BEpv-jKQ

$ tclsh seguid --type=ldseguid <<< 'TATGCCAA;TTGGCATA'
ldseguid=p88RYs41n0NTej4htM1fJAWI1ME
```

Documentation, examples, and online WebAssembly-based [28] demos are available on `https://www.seguid.org`. Support for additional programming languages are planned, specifically C++ and Rust, and will be made available via the SEGUID website.

Validation and testing

It is essential that any implementation of the SEGUID v2 checksum algorithm is correct and comply with the SEGUID v2 specifications as described here. For instance, we do not want to incorrectly produced checksums to be used in production, appear in publications, or entering shared databases. We provide a rich set of unit and redundancy tests (see SEGUID website) to lower the risk for this to ever occur. These tests assert correctness, and identical behavior and results regardless of which programming language was used for the implementation. All new implementations must adhere to these tests in order to be considered SEGUID v2 compliant. These tests run on all releases and on a nightly basis. This test framework is designed to make it as simple as possible for a third-party contributor to run these tests. We use continuous-integration testing to validate all known implementations.

Higher-level implementations

The main purpose of the above **seguid** packages is to provide a minimal, language-agnostic API of the SEGUID v2 algorithm that can be imported in higher-level implementations. The scope of these packages will be kept at a minimum to avoid feature creep of both of the API and the SEGUID v2 algorithm. This way, the packages also serve as reference implementations and help lower the threshold for implementing the API in other languages.

For these reasons, we consider tasks like coercing a DNA sequence in lower-case letters to upper-case letters, trimming strings, and converting a sequence to its complementary counterpart to be out of scope. Such features have to be implemented by other tools and packages, e.g. Python packages **pydna** [29] and **Biopython** [30, 31]. For example, **pydna** implements the SEGUID v2 methods for the `Dseqrecord` class, which holds double-stranded DNA. It also provides a rich set of utility functions for operating on DNA and RNA sequences. **Biopython** already implements the original SEGUID method and we anticipate it will also provide SEGUID v2 functionalities in a future release.

Graphical user interfaces (GUIs)

Some users are not comfortable with the provided command-line interfaces, or inexperienced with the supported programming languages. In order to make SEGUID v2 accessible to these users it is important the method is also available through a Graphical User Interface (GUI). A readily available GUI (Graphical User Interface) will boost the adoption of SEGUID v2 to a broader audience as it also caters to users of varying expertise levels. Below are two GUIs that can be used for calculating SEGUID checksums.

Fig 7. GUI implementations of SEGUID checksums. Two screenshots of GUI tools for calculating SEGUID v2 checksums. The **SEGUID Calculator** tool (left) calculates the different types of SEGUID v2 checksums based on the Watson sequence as it is being entered. This a JavaScript-based implementation that fully runs within the web browser. The A plasmid Editor (**ApE**) tool (right) is specifically designed for double-stranded DNA. Because of this, it is sufficient to enter the Watson sequence, from which the Crick sequence is automatically inferred.

The **SEGUID Calculator** (Fig 7) is a browser-based GUI for the SEGUID v2 algorithm available via the SEGUID website. It is implemented in JavaScript and allows for computations such that entered data never leaves the web browser or the

local computer. This type of instant interactive feedback enhances understanding of how the checksum works.

The popular, freely available **ApE** (A plasmid Editor) [32] allows calculating and stamping dsDNA sequences with SEGUID v2 checksums (Fig 7). **ApE** is built on our Tcl implementation and available from the Jorgensen Lab website[9].

Applications

SEGUID checksums can be used to map sequence metadata both within and across biological databases. For example, by indexing different types of identifiers based on SEGUID checksums, the SEGUID checksum can serve as the common denominator across heterogeneous databases. For example, this technique was used at the Argonne National Laboratory to build a real-time, interactive search engine for protein annotation data collected from multiple resources. For motivations similar to those of the Short ID, the search engine was designed to list matching candidates as soon as the user had typed three letters (Fig 8).

Analogously to how SEGUID checksums can be used for quick lookup of sequence data via database tools, they can be used for navigating and looking up sequence data hosted directly on the file system. For example, sequence-annotation data files of different kinds can be grouped in folders named as the corresponding SEGUID v2 checksums, or the individual files can comprise the checksum.

Fig 8. Type-ahead database search based on partial SEGUID checksums. Illustration of a how partial SEGUID checksums can be used to autocomplete searches as you type. In this example, the user enters `PRO` and the Ajax-based web interface instantaneously returns all SEGUID checksums starting with those characters. Sequence data is pulled from multiple databases concurrently for entries matching the selected SEGUID checksum.

Fig 9. SEGUID checksums added to GenBank sequence files. The files were stamped with SEGUID checksum using the **ApE** tool (upper panel) or the **pydna** Python package (lower panel). In both cases, the checksum is added in the `COMMENT` field of the GenBank record. In addition, **pydna** appends an ISO-8601 timestamp after the checksum.

The **ApE** tool and the **pydna** package can stamp and save sequence files in GenBank format[10] where the checksum is saved in the `COMMENT` section of the GenBank file (Fig 9). An existing checksum can be verified by each tool.

Fig 10. Cloning exercise. Students, in undergraduate courses at the Department of Biology of the University of Minho, are given a cloning simulation problem (top) that will result in two vectors. They are given the Short IDs of the correct vectors for them to self-correct before filing the results.

A common use of the SEGUID checksum is in undergraduate teaching of practical molecular biology in the Department of Biology at the University of Minho. An example problem is depicted in Fig 10. The students are given a task, typically to simulate a sub-cloning problem on their computers. This kind of problem has a unique

[9] `https://jorgensen.biology.utah.edu/wayned/ape`
[10] `https://www.ncbi.nlm.nih.gov/genbank/samplerecord/`

correct answer, making it possible to calculate checksums for the correct solution beforehand. The problem of assessing the students is simplified by the students access to a local or web-based SEGUID-capable software, e.g. **SEGUID Calculator**. Giving access to a partial checksum for the correct answer can help the students to rapidly determine whether their procedures are incorrect. We have found this approach practical to teach in-silico cloning to over 60 students at a time. Overall, it lowers the number of mistakes filed and the efforts on reviewing homework assignments.

Discussions

The SEGUID v2 method produces checksums for various types of biological sequences, including, but not limited to, DNA, RNA, and proteins. The method is invariant to orientation of double-stranded sequences, and invariant to rotation for circular sequences. In contrast to other methods (e.g. Bouras et al. (2024) [33]), SEGUID v2 is also invariant to genome and does not require the input to comprise known gene sequences.

The SEGUID v2 checksum is capable of providing the scientific community with stable keys for the many thousands of plasmids created in the process of designing new recombinant organisms or synthetic biological constructs. Important plasmid sequences can be stored in a distributed fashion, without the need for expensive centralized infrastructure. The integrity of sequences distributed among members of the scientific community can easily be verified. Using SEGUID v2 makes it trivial to confirm that members of a research groups are all using the same sequence for a particular DNA fragment containing a genetic part. The SEGUID v2 checksum would also enable a global reference service for circular DNA, such as the registry provided by EMBL-EBI at identifiers.org. Since the checksum is already implemented in the popular DNA editor ApE, we believe this will encourage wider adoption by both researchers and web-facing applications.

Acknowledgments

We would like to express our gratitude to the undergraduate students of Applied Biology and Biological Engineering at the University of Minho who have helped us to develop this work over the years.

References

1. Kamens J. The Addgene repository: an international nonprofit plasmid and data resource. Nucleic Acids Research. 2014 11;43(D1):D1152-7. Available from: `https://doi.org/10.1093/nar/gku893`. doi:10.1093/nar/gku893.

2. Kamens J. Addgene: Making Materials Sharing "Science As Usual". PLOS Biology. 2014 11;12(11):1-5. Available from: `https://doi.org/10.1371/journal.pbio.1001991`. doi:10.1371/journal.pbio.1001991.

3. H PW, P FB, A TS, T VW. In: Cyclic redundancy and other checksums. 2nd ed. Cambridge University Press; 1993. p. 896-902.

4. Babnigg G, Giometti CS. A database of unique protein sequence identifiers for proteome studies. Proteomics. 2006 Aug;6(16):4514-22. Available from:

https://onlinelibrary.wiley.com/doi/10.1002/pmic.200600032. doi:10.1002/pmic.200600032.

5. FIPS PUB 180-4. Secure Hash Standard (SHS). Information Technology Laboratory, National Institute of Standards and Technology, Gaithersburg, MD 20899-8900: National Institute of Standards and Technology (NIST, U.S.); 2015. Federal Information Processing Standards Publication (FIPS PUB 180-4). doi:10.6028/nist.fips.180-4.

6. IUPAC-IUB Commission on Biochemical Nomenclature. Abbreviations and symbols for nucleic acids, polynucleotides, and their constituents. Biochemistry. 1970;9(20):4022-7. Available from: https://doi.org/10.1021/bi00822a023. arXiv:https://doi.org/10.1021/bi00822a023. doi:10.1021/bi00822a023.

7. Jeck WR, Sorrentino JA, Wang K, Slevin MK, Burd CE, Liu J, et al. Circular RNAs are abundant, conserved, and associated with ALU repeats. RNA. 2013 Feb;19(2):141-57.

8. Blond A, Péduzzi J, Goulard C, Chiuchiolo MJ, Barthélémy M, Prigent Y, et al. The cyclic structure of microcin J25, a 21-residue peptide antibiotic from Escherichia coli. European Journal of Biochemistry. 1999;259(3):747-56. Available from: https://febs.onlinelibrary.wiley.com/doi/abs/10.1046/j.1432-1327.1999.00085.x. arXiv:https://febs.onlinelibrary.wiley.com/doi/pdf/10.1046/j.1432-1327.1999.00085.x. doi:https://doi.org/10.1046/j.1432-1327.1999.00085.x.

9. Trabi M, Craik DJ. Circular proteins–no end in sight. Trends Biochem Sci. 2002 Mar;27(3):132-8.

10. Pierre Duval J. Factorizing words over an ordered alphabet. Journal of algorithms & computational technology. 1983 Dec;4(4):363-81. Available from: https://www.sciencedirect.com/science/article/pii/0196677483900172. doi:10.1016/0196-6774(83)90017-2.

11. Rivest RL. The MD5 Message-Digest Algorithm. RFC Editor; 1992. RFC 1321. Available from: https://www.rfc-editor.org/info/rfc1321. doi:10.17487/RFC1321.

12. Wang X, Yu H. How to Break MD5 and Other Hash Functions. In: Cramer R, editor. Advances in Cryptology – EUROCRYPT 2005. Berlin, Heidelberg: Springer Berlin Heidelberg; 2005. p. 19-35.

13. Hart RK, Prlić A. SeqRepo: A system for managing local collections of biological sequences. PLOS ONE. 2020;15(12):e0239883. doi:10.1371/journal.pone.0239883.

14. Stevens M, Bursztein E, Karpman P, Albertini A, Markov Y. The First Collision for Full SHA-1. In: Katz J, Shacham H, editors. Advances in Cryptology – CRYPTO 2017. Cham: Springer International Publishing; 2017. p. 570-96.

15. Leurent G, Peyrin T. SHA-1 is a Shambles: First Chosen-Prefix Collision on SHA-1 and Application to the PGP Web of Trust. In: 29th USENIX Security Symposium (USENIX Security 20). USENIX Association; 2020. p. 1839-56. Available from: https://www.usenix.org/conference/usenixsecurity20/presentation/leurent.

16. Computer Security Resource Center (CSRC). NIST Policy on Hash Functions; 2022. The U.S. National Institute of Standards and Technology. Available from: https://csrc.nist.gov/projects/hash-functions/nist-policy-on-hash-functions.

17. Josefsson S. The Base16, Base32, and Base64 Data Encodings; 2006. RFC 4648. Available from: https://www.rfc-editor.org/rfc/pdfrfc/rfc4648.txt.pdf. doi:10.17487/RFC4648.

18. IUPAC-IUB Commission on Biochemical Nomenclature. Nomenclature and Symbolism for Amino Acids and Peptides. European Journal of Biochemistry. 1984;138(1):9-37. Available from: https://febs.onlinelibrary.wiley.com/doi/abs/10.1111/j.1432-1033.1984.tb07877.x. arXiv:https://febs.onlinelibrary.wiley.com/doi/pdf/10.1111/j.1432-1033.1984.tb07877.x. doi:https://doi.org/10.1111/j.1432-1033.1984.tb07877.x.

19. Viner C, Ishak CA, Johnson J, Walker NJ, Shi H, Sjöberg-Herrera MK, et al. Modeling methyl-sensitive transcription factor motifs with an expanded epigenetic alphabet. Genome Biology. 2024 jan;25(1):11. Available from: https://doi.org/10.1186/s13059-023-03070-0. doi:10.1186/s13059-023-03070-0.

20. Wang Z, Mosbaugh DW. Uracil-DNA glycosylase inhibitor of bacteriophage PBS2: cloning and effects of expression of the inhibitor gene in Escherichia coli. Journal of Bacteriology. 1988;170(3):1082-91. Available from: https://journals.asm.org/doi/abs/10.1128/jb.170.3.1082-1091.1988. arXiv:https://journals.asm.org/doi/pdf/10.1128/jb.170.3.1082-1091.1988. doi:10.1128/jb.170.3.1082-1091.1988.

21. Muha V, Horváth A, Békési A, Pukáncsik M, Hodoscsek B, Merényi G, et al. Uracil-containing DNA in Drosophila: stability, stage-specific accumulation, and developmental involvement. PLoS genetics. 2012 Jun;8(6):e1002738. Available from: http://dx.doi.org/10.1371/journal.pgen.1002738. doi:10.1371/journal.pgen.1002738.

22. Muramatsu M, Kinoshita K, Fagarasan S, Yamada S, Shinkai Y, Honjo T. Class switch recombination and hypermutation require activation-induced cytidine deaminase (AID), a potential RNA editing enzyme. Cell. 2000 Sep;102(5):553-63. Available from: http://dx.doi.org/10.1016/s0092-8674(00)00078-7. doi:10.1016/s0092-8674(00)00078-7.

23. Geu-Flores F, Nour-Eldin HH, Nielsen MT, Halkier BA. USER fusion: a rapid and efficient method for simultaneous fusion and cloning of multiple PCR products. Nucleic acids research. 2007 Mar;35(7):e55. Available from: http://dx.doi.org/10.1093/nar/gkm106. doi:10.1093/nar/gkm106.

24. ECMA International. ECMAScript 2024 Language Specification; 2024. https://www.ecma-international.org/publications-and-standards/standards/ecma-262/.

25. van Rossum G, de Boer J. Interactively testing remote servers using the Python programming language. CWI Quarterly. 1991 Dec;4(4):283-304.

26. R Core Team. R: A Language and Environment for Statistical Computing. Vienna, Austria; 2024. Available from: https://www.R-project.org/.

27. Ousterhout JK, Jones K. Tcl and the Tk Toolkit. Addison-Wesley Professional Computing Series. Pearson Education; 2009.

28. Haas A, Rossberg A, Schuff DL, Titzer BL, Holman M, Gohman D, et al. Bringing the web up to speed with WebAssembly. ACM SIGPLAN Notices. 2017;52(6):185-200. doi:10.1145/3140587.3062363.

29. Pereira F, Azevedo F, Carvalho Â, Ribeiro GF, Budde MW, Johansson B. Pydna: a simulation and documentation tool for DNA assembly strategies using python. BMC bioinformatics. 2015 May;16(142):142. Available from: `http://dx.doi.org/10.1186/s12859-015-0544-x`. doi:10.1186/s12859-015-0544-x.

30. Cock PJA, Antao T, Chang JT, Chapman BA, Cox CJ, Dalke A, et al. Biopython: freely available Python tools for computational molecular biology and bioinformatics. Bioinformatics. 2009 Jun;25(11):1422-3. Available from: `http://dx.doi.org/10.1093/bioinformatics/btp163`. doi:10.1093/bioinformatics/btp163.

31. Bassi S, Gonzalez V. New checksum functions for Biopython. Nature Precedings. 2007 Jun:1-1. Available from: `https://www.nature.com/articles/npre.2007.278.1`. doi:10.1038/npre.2007.278.1.

32. Davis MW, Jorgensen EM. ApE, A Plasmid Editor: A Freely Available DNA Manipulation and Visualization Program. Frontiers in Bioinformatics. 2022;2. Available from: `https://www.frontiersin.org/article/10.3389/fbinf.2022.818619`. doi:10.3389/fbinf.2022.818619.

33. Bouras G, Grigson SR, Papudeshi B, Mallawaarachchi V, Roach MJ. Dnaapler: A tool to reorient circular microbial genomes. Journal of Open Source Software. 2024;9(93):5968. Available from: `https://doi.org/10.21105/joss.05968`. doi:10.21105/joss.05968.

Figure 1

Click here to access/download;Figure;fig01-crc64-collision.pdf

>AAF20475.1 crc64=<u>44CAAD88706CC153</u> lsseguid=X5XEaayob1nZLQc7eVT9qyczarY

QSALTQPASVSGSPGQSITISCTGTSSDVGSYNLVSWYQQHPGKAPKLMIYEGSKRPSGVSNRFSGSKSGNTASLTISGLQAEDEADYYCCSYAGSSTWVFGGGTKLTVL

⇑ ⇑

>AAF20481.1 crc64=<u>44CAAD88706CC153</u> lsseguid=BpBeDdcNUYNsdk46JoJdw7Pd3BI

QSALTQPASVSGSPGQSITISCTGTSSDVGSYNLVSWYQQHPGKAPKLMIYEGSKRPSGVSNRFSGSKSGNTASLTISGLQAEDEADYYCSSYAGSSTLVFGGGTKLTVL

⇑ ⇑

Figure 2

Click here to access/download;Figure;fig02

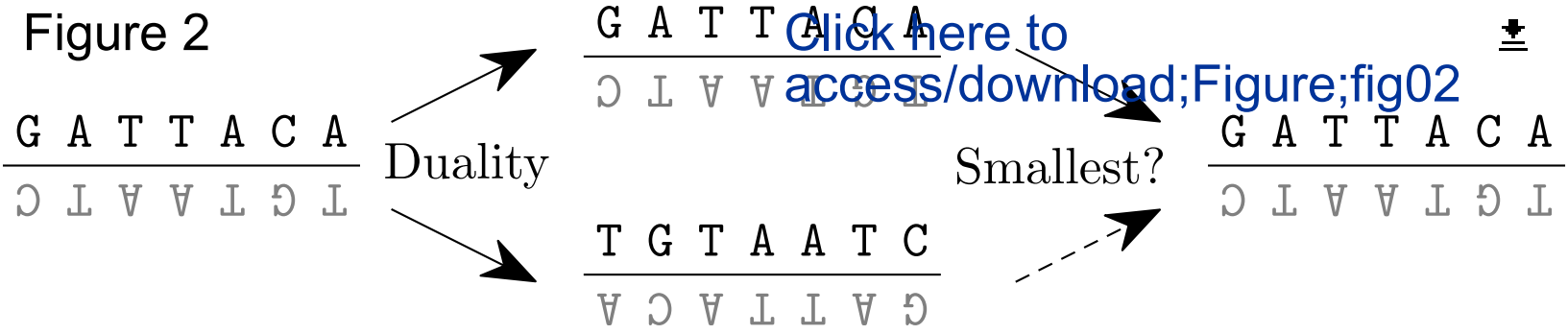

Figure 3

Click here to access/download;Figure;fig03

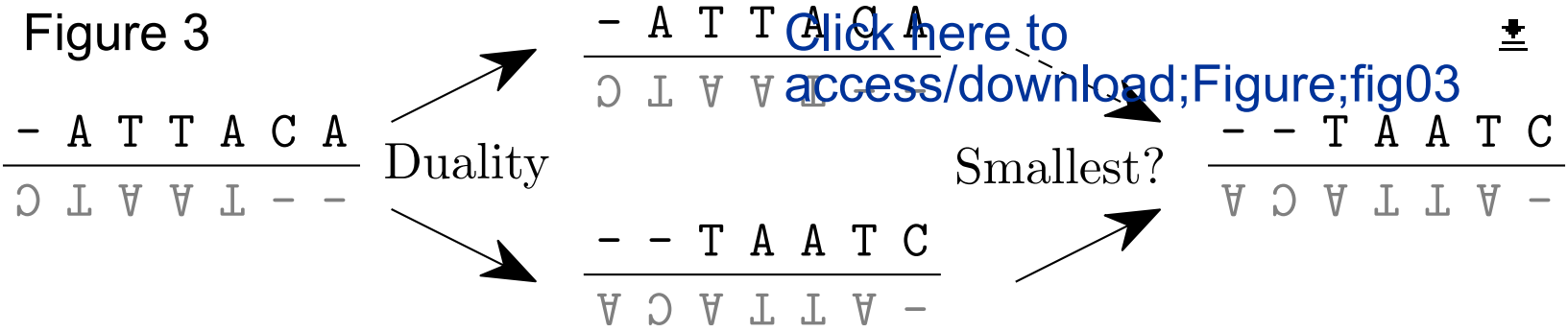

Figure 4

Click here to access/download;Figure;fig04-

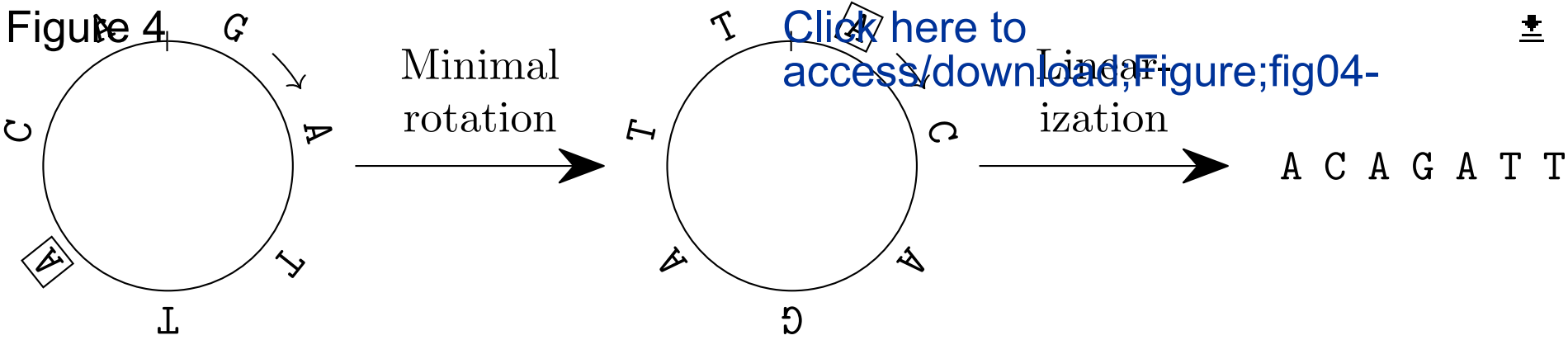

Figure 5

Click here to access/download;Figure;fig05-cdseguid.pdf

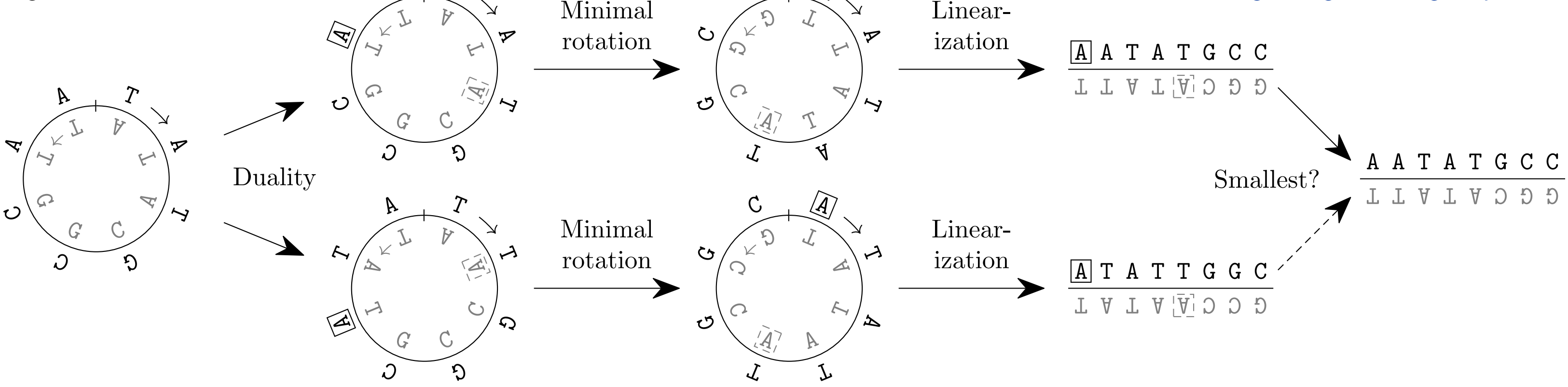

Figure 6

Click here to access/download;Figure;fig06

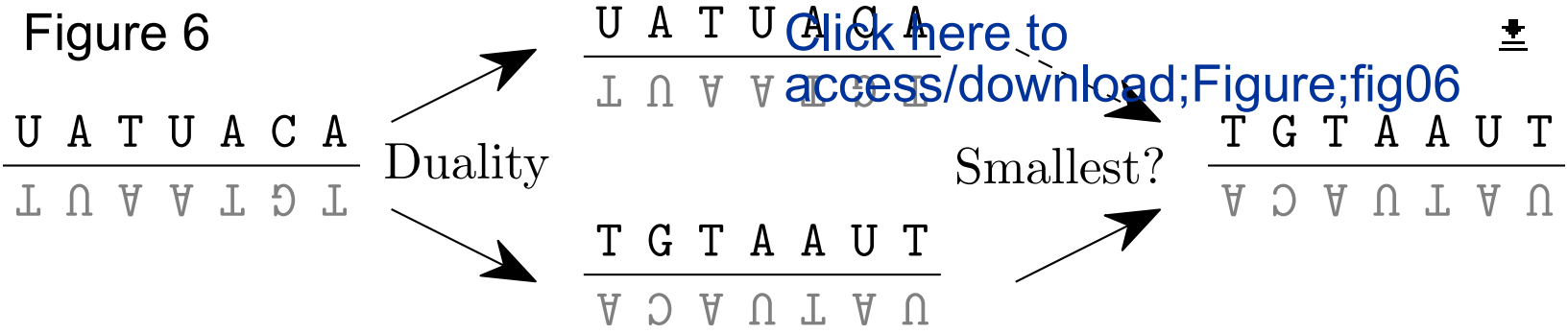

Figure 7

Click here to access/download;Figure;fig07-seguid-calculator_ape.png

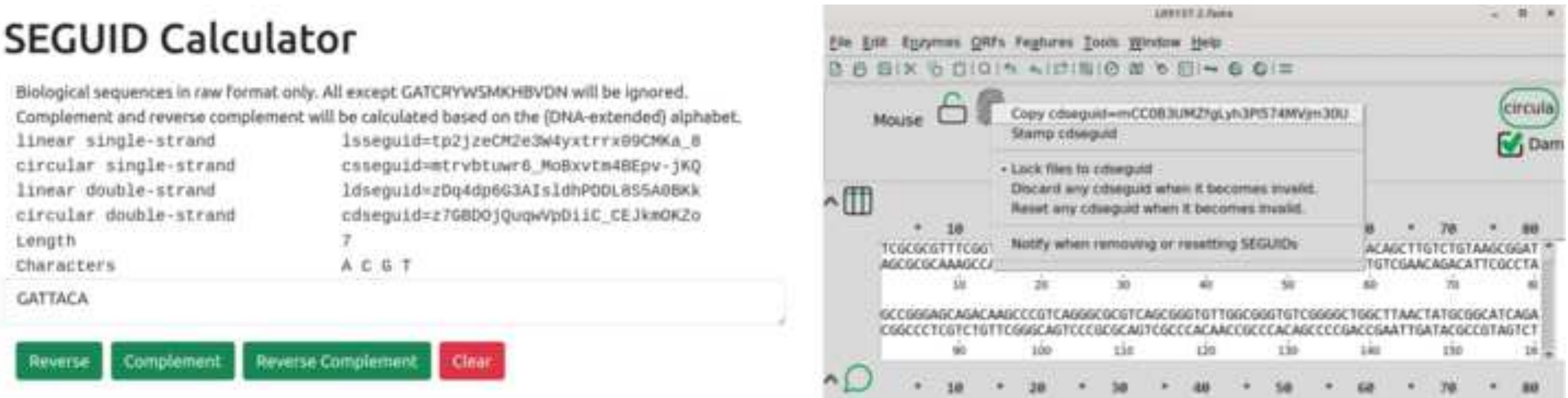

Figure 8

Click here to access/download;Figure;fig08-seguid-ajax-gui.png

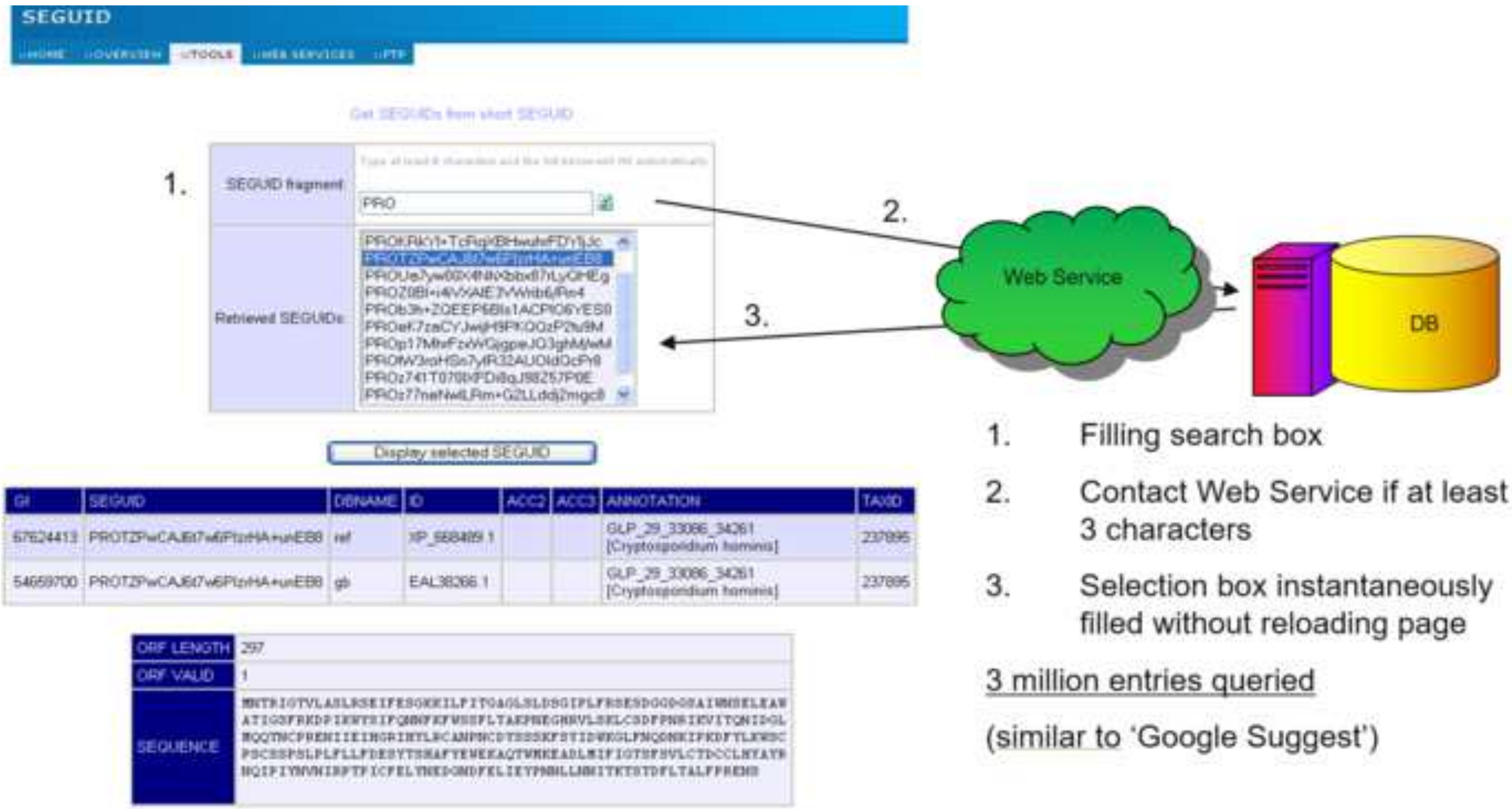

Figure 9

Click here to access/download;Figure;fig09-genbank_file_by_ape_and_pydna.pdf

ApE

```
LOCUS       New_DNA                            7 bp    DNA     circular  10-MAY-2023
DEFINITION  .
ACCESSION
VERSION
SOURCE      .
  ORGANISM  .
COMMENT     ApEinfo:methylated:0
COMMENT     ApEinfo:csseguid=mtrvbtuwr6_MoBxvtm4BEpv-jKQ
ORIGIN
        1 gattaca
//
```

---

pydna

```
LOCUS       name                               7 bp    DNA     circular UNK 01-JAN-1980
DEFINITION  description.
ACCESSION   id
VERSION     id
KEYWORDS    .
SOURCE      .
  ORGANISM  .
COMMENT     pydna csseguid=mtrvbtuwr6_MoBxvtm4BEpv-jKQ 2023-10-13T10:11:54
FEATURES    Location/Qualifiers
ORIGIN
        1 gattaca
//
```

Click here to access/download;Figure;fig10-Cloning_exercise_Instructions.pdf

Cloning exercise

The plasmid vector pUC19 (2,686 bp, `cdseguid=mCC0B3UMZfgLyh3Pl574MVjm30U`) is a widely used cloning vector. The sequence is available from GenBank under the accession number `L09137.2`. Simulate the non-directional cloning of the dsDNA fragment below:

```
gatccATGCAATAAg
    gTACGTTATTcctag
```

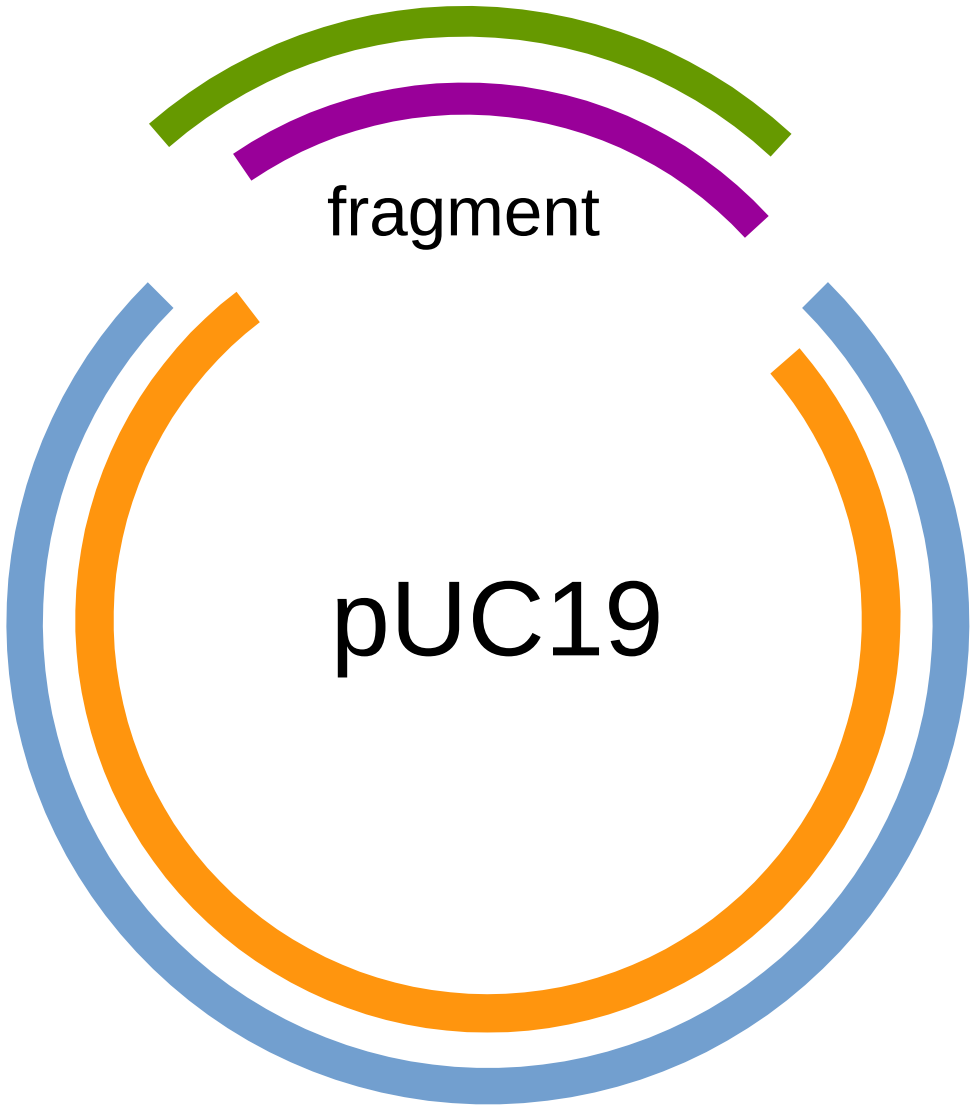

pUC19 was linearized with *Bam*HI. Enter the SEGUID checksums for the two resulting vectors (pUC19_frag_A and pUC19_frag_B) in the **shared spreadsheet** next to your name.

Sequences	Short ID
pUC19_frag_A	**CzM-IG**
pUC19_frag_B	**S4WZki**

Click here to access/download
Supporting Information
Pereira_etal_2024-SEGUIDv2-SupplNotes.pdf

Click here to access/download
Supporting Information - Compressed/ZIP File Archive
Pereira_etal_2024-SEGUIDv2-SupplData.zip

SEGUID v2: Extending SEGUID checksums for circular, linear, single- and double-stranded biological sequences

Humberto Pereira[1], Paulo César Silva[1], M. Wayne Davis[2], Louis Abraham[3], György Babnigg[4], Henrik Bengtsson[5,6◕*], Björn Johansson[1◕]

1 Center of Molecular and Environmental Biology Engineering, University of Minho, Campus de Gualtar, Braga, 4710-057, Portugal
2 Howard Hughes Medical Institute and School of Biological Sciences, University of Utah, Salt Lake City, UT, USA
3 PRISM, Université Paris 1, Panthéon–Sorbonne, Paris, France
4 Biosciences Division, Argonne National Laboratory, Argonne, IL, USA
5 Department of Epidemiology and Biostatistics, University of California, San Francisco, CA, USA
6 Helen Diller Family Comprehensive Cancer Center, University of California, San Francisco, CA, USA

◕These authors contributed equally to this work.
* corresponding author henrik.bengtsson@ucsf.edu

Abstract

Background: Synthetic biology involves combining different DNA fragments, each containing functional biological parts, to address specific problems. Fundamental gene-function research often requires cloning and propagating DNA fragments, such as those from the iGEM Parts Registry or Addgene, typically distributed as circular plasmids. Addgene's repository alone offers around 150,000 plasmids.

To ensure data integrity, cryptographic checksums can be calculated for the sequences. Each sequence has a unique checksum, making checksums useful for validation and quick lookups of associated annotations. For example, the SEGUID checksum, uniquely identifies protein sequences with a 27-character string.

Objectives: The original SEGUID, while effective for protein sequences and single-stranded DNA (ssDNA), is not suitable ~~for circular and double-stranded DNA (dsDNA) due to topological differences~~ DNA since there is no natural starting position nor for double-stranded DNA (dsDNA) since two separate sequences are present. Challenges include how to uniquely represent linear dsDNA, circular ssDNA, and circular dsDNA. To meet these needs, we propose SEGUID v2, which extends the original SEGUID to handle additional types of sequences.

Conclusions: SEGUID v2 produces orientation and rotation invariant checksums for single-stranded, double-stranded, possibly staggered, linear, and circular DNA and RNA sequences. Customizable alphabets allow for other types of sequences. In contrast to the original SEGUID, which uses Base64, SEGUID v2 uses Base64url to encode the SHA-1 hash. This ensures SEGUID v2 checksums can be used as-is in filenames, regardless of platform, and in URLs, with minimal friction.

Availability: SEGUID v2 is readily available for major programming languages, distributed under the MIT license. JavaScript package **seguid** is available on npm, Python package **seguid** on PyPi, R package **seguid** on CRAN, and a Tcl script on GitHub. These tools, along with documentation, examples, and an online **SEGUID Calculator**, can be found at `https://www.seguid.org`.

Introduction

The post-genomic era has provided access to more biological sequence data than ever before. These sequence data provide an important resource for both fundamental research and synthetic biology, which aim to understand and harness biology to solve urgent societal problems, such as global warming or food security. Genetic parts contained on DNA fragments are distributed to and from culture collections and repositories, as well as within the research community to study genes and build new synthetic systems. These parts are almost always distributed as plasmids, where the DNA of interest is inserted into a circular double-stranded (ds) DNA plasmid providing replication and selection in a microorganism optimal for producing high-quality DNA, usually *E. coli*.

Each plasmid should have an associated sequence file that is distributed along with the physical clone. Although repositories such as Addgene [1, 2] and the iGEM (International Genetically Engineered Machine) Parts collection provide standards, the transmission of biological material and associated information is still very much an ad-hoc procedure.

Data integrity is of great importance since it directly affects the reproducibility of science that relied on the original genetic parts. Checksums are widely used for verifying that information is preserved through space and time, and has also been applied to safe-guard biological sequences. A checksum is a short string of characters that is calculated from, and typically stored with, arbitrarily sized data. By transmitting the checksum together with the data, a receiver can verify correctness of the data.

```
>AAF20475.1 crc64=44CAAD88706CC153 lsseguid=X5XEaayob1nZLOc7eVT9qyczarY
QSALTQPASVSGSPGQSITISCTGTSSDVGSYNLVSWYQQHPGKAPKLMIYEGSKRPSGVSNRFSGSKSGNTASLTISGLQAEDEADYYCCSYAGSSTWVFGGGTKLTVL
                                                                                          ⇑       ⇑
>AAF20481.1 crc64=44CAAD88706CC153 lsseguid=BpBeDdcNUYNsdk46JoJdw7Pd3BI
QSALTQPASVSGSPGQSITISCTGTSSDVGSYNLVSWYQQHPGKAPKLMIYEGSKRPSGVSNRFSGSKSGNTASLTISGLQAEDEADYYCSSYAGSSTLVFGGGTKLTVL
                                                                                          ⇑       ⇑
```

Fig 1. Two protein sequences with colliding CRC-64 checksums. The two protein sequences with GenBank accession number *AAF20481.1*$_{\mathrm{GB}}$ and *AAF20475.1*$_{\mathrm{GB}}$ have both 110 amino acids and differ at two positions (91 and 99). Despite this, they share the same UniProt **CRC-64** checksum. The **SHA-1**-based SEGUID v2 checksums differ.

Several existing checksum algorithms are relevant in bioinformatics. One of the first used was the cyclic redundancy check (**CRC**) [3], which is a computationally inexpensive, error-detecting algorithm originally designed for network and storage devices. The UniProt database initially used a 64-bit **CRC** (**CRC-64**) checksum[1]. This is the reason why the UniProt Archive (UniParc) still provides **CRC-64**[2]. A disadvantage of the **CRC-64** is that collisions have been observed for biological data. For example, the two protein sequences with GenBank accession numbers *AAF20475.1*$_{\mathrm{GB}}$[3] and *AAF20481.1*$_{\mathrm{GB}}$[4], have the same **CRC-64** hexadecimal checksum, despite differing in two of the 110 amino acids (Fig 1).

While collisions are unavoidable for all functions producing finite-length checksums, longer checksums are less likely to generate collisions. However, long checksums are less readable and also more expensive to generate, store, and compare. The SEquence Globally Unique IDentifier (SEGUID) checksum [4] was introduced in order to provide

[1]https://www.uniprot.org/help/checksum
[2]https://www.uniprot.org/help/uniparc
[3]UniParc accession number *UPI0000117F35*$_{\mathrm{UP}}$
[4]UniParc accession number *UPI0000117F39*$_{\mathrm{UP}}$

a stable, unifying key for the same sequence in different databases facilitating linking protein sequences across databases. SEGUID is based on the **SHA-1** (Secure Hash Algorithm 1) algorithm [5], which produces a 160-bit hash, and is therefore less sensitive to collisions compared with the 64-bit **CRC-64** hash. With **SHA-1**, sequence collisions are very unlikely. For example, the two proteins with clashing **CRC-64** checksums (Fig 1) have distinct SEGUID checksums.

More importantly, no current checksum algorithm is directly useful for DNA, since the topology of DNA is most often different from that of proteins. Double-stranded DNA (dsDNA) consists of two complementary, anti-parallel strands. Usually, only one is stored for DNA, as the second strand is the complement of the stored sequence. However, both strands are equally valid representations of the DNA molecule, but give different checksum values, except for the special case where the sequence is a palindrome and the strands therefore identical.

DNA may be circular, e.g. circular single-stranded DNA (ssDNA) or circular dsDNA. For these cases, there are multiple representations for the same circular sequence, and there is no well-defined canonical representation for which the checksum should be calculated.

In this article, we propose SEGUID v2, which is an extension to the original SEGUID, that produces checksums for linear, circular, single- and double-stranded sequences of different types, including DNA, RNA, and amino-acid sequences in order to integrate synthetic biology resources and identify redundancies in existing databases.

We believe that a stable checksum that describes any biological sequence, regardless of topology or strandedness, would streamline the exchange of information and biological materials within the synthetic biology community.

Methods

The problem of defining a checksum for sequences of different kinds, comprise three challenges: (i) identify a unique representation among one or more equivalent representations, and convert it to a linear sequence representation, (ii) choose a hash function to distill the unique representation to a large-enough bit sequence, and (iii) compactly encode the bit sequence to a checksum using a text-friendly encoding alphabet.

Below, we explain how we define each of these steps for the proposed SEGUID v2 checksum, and what objectives we target when there are multiple options available.

Unique representation of sequence data

Linear single-stranded sequences

A linear single-stranded sequence is a single, directional sequence with one or more symbols, where directional means it has a unique start and end ("left to right"). The sequence may be amino acids, DNA, RNA, or any sequence of symbols from a well-defined, case-sensitive alphabet. Examples of DNA sequences are `C`, `AT`, and `GATTACA`. Examples of RNA sequences are `C`, `AU`, and `AGA`. Examples of amino-acid sequences ("proteins") are `A`, `QSA`, and `QSALTQPASV`.

A linear single-stranded sequence is always unique, which makes it natural to use the sequence as-is to calculate the checksum. This case was already covered by the original SEGUID algorithm.

Linear double-stranded sequences

A linear double-stranded sequences comprise two sequences that share a region where the two sequences are complementary to each other. For double-stranded DNA, the $5'\rightarrow 3'$ "upper" strand is often referred to as the "Watson" strand and the complementary "lower" as the "Crick" strand. We use the Watson and Crick strands separated by the symbol ↩, where both Watson and Crick are $5'\rightarrow 3'$ sequences, to represent a double-stranded sequence. Using this notation, the double-stranded sequence `GATTACA↩TGTAATC` reflects the graphical representation in Fig 2.

Two types of such sequences must be considered - (i) blunt-ended, and (ii) staggered, linear double-stranded sequences - which are discussed next.

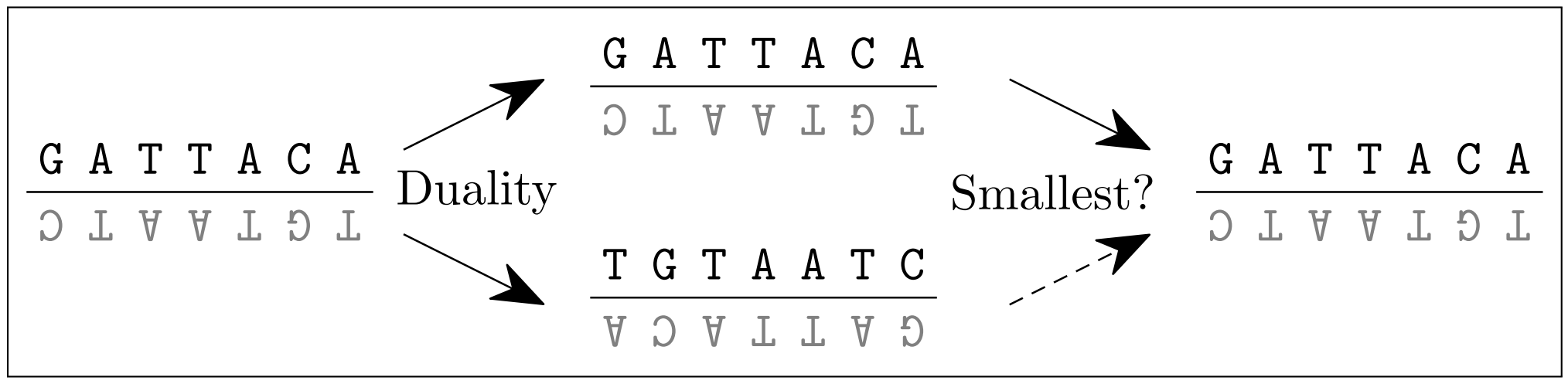

Fig 2. The SEGUID v2 steps for identifying the canonical representation of a blunt-ended linear, double-stranded DNA sequence. The input sequence `GATTACA↩TGTAATC` (left and top middle) has a dual, complementary representation `TGTAATC↩GATTACA` (bottom middle). The lexicographically smallest of these two is `GATTACA↩TGTAATC` (right). It is from this linear representation that the SEGUID v2 checksum is calculated. Legend: Following IUPAC-IUB Commission on Biochemical Nomenclature (1970) [6], we choose to rotate the symbols on the complementary strand in the figure such that the left-to-right, $3'\rightarrow 5'$ representation holds when rotating the figure upside-down.

Blunt-ended, linear double-stranded sequences: In its basic form, the two sequences are of the same length and fully overlapping, i.e. the shared region spans their full length. We refer to this type of sequences as blunt-ended (short blunt), or non-staggered, double-stranded sequences. For example, `GATTACA↩TGTAATC` is a blunt, double-stranded sequence with complementary strands `GATTACA` and `TGTAATC`. Importantly, `GATTACA↩TGTAATC` and `TGTAATC↩GATTACA` are dual representations of the same linear double-stranded DNA sequence (Fig 2). In other words, without further constraints, there is no unique representation of it. To address this uniqueness problem, we define the unique representation of the two possibilities as the one that is lexicographically smaller[5] than the other. This is possible for all well-defined alphabets. For example, consider the DNA alphabet `A`, `C`, `G`, `T`, ordered such that `A` $<$ `C` $<$ `G` $<$ `T`. This allows us to compare any two sequences lexicographically, e.g. `AT` $<$ `TA`. For example, in the above example, we have that `GATTACA↩TGTAATC` $<$ `TGTAATC↩GATTACA`, and any checksums will be calculated based on the first of these two representations (Fig 2).

Staggered, linear double-stranded sequences: Another form of double-stranded sequences is where the two sequences do not fully overlap. We refer to this type of sequences as staggered double-stranded sequences, e.g. `-ATTACA↩TGTAATC`, `--TTACA↩TGTAATC`, and `-ATTACA↩-GTAATC`. Here we use the minus symbol (`-`) to indicate that there is a symbol on the opposite strand but not on the current strand. As for blunt double-stranded sequences, staggered ones have two possible representation for the same sequence, e.g. `-GTAATC↩--TTACA` and `--TTACA↩-GTAATC`

[5] Strictly speaking, "smaller or equal" is sufficient to get a unique representation.

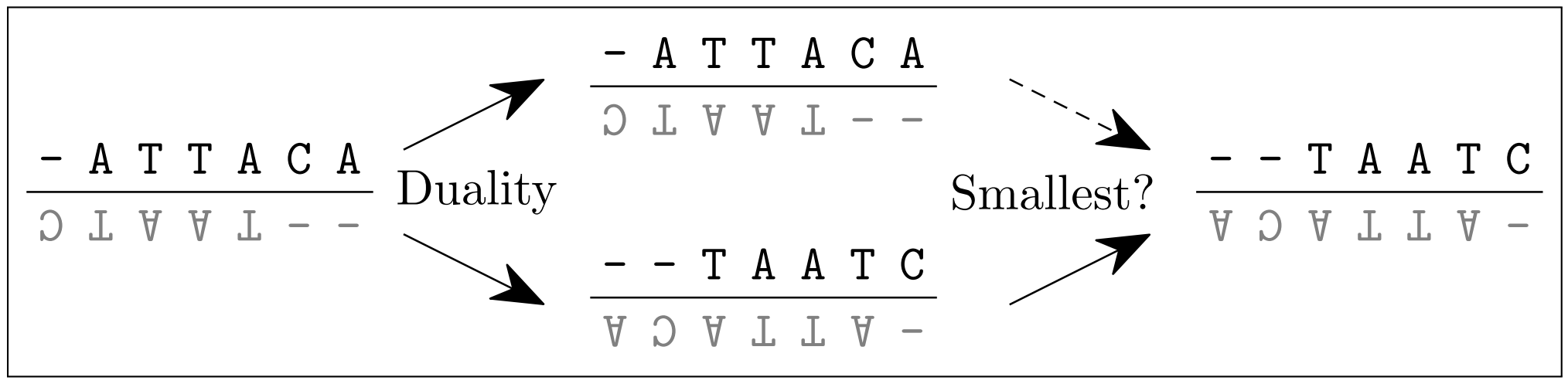

Fig 3. The SEGUID v2 steps for identifying the canonical representation of a staggered, linear, double-stranded DNA sequence. The input sequence `-ATTACA↩--TAATC` (left and top middle) has a dual, complementary representation `--TAATC↩-ATTACA` (bottom middle). Because the minus symbol (`-`) is lexicographically smaller than the other symbols, the smallest of these two linear representations is `--TTACA↩-GTAATC` (right). It is from this linear representation that the SEGUID v2 checksum is calculated.

refer to the same sequence. As before, we define the unique representation to be the lexicographically smallest of the two, where we define the minus symbol (`-`) to be smaller than any other symbol, e.g. with the DNA alphabet we have that `-` < `A` < `C` < `G` < `T`. With this, we can identify `--TTACA↩-GTAATC` as the minimal lexicographic representation, because `--TTACA↩-GTAATC` < `-GTAATC↩--TTACA` (Fig 3). It is from this canonical representation that the hash is calculated.

Circular single-stranded sequences

A circular single-stranded sequence is a single, directional sequence with one or more symbols without a beginning or end. The sequence may comprise of symbols from any well-defined alphabet. Any linear single-stranded sequence can be made a circular single-stranded sequences, by combining the two ends, and vice versa. Examples of circular DNA sequences are `C`↻, `AT`↻, `TA`↻, and `GATTACA`↻. Here we use the symbol ↻ to indicate that the two ends are combined. A naturally occurring circular, single-stranded DNA sequences is the 6,407-nucleotide M13 bacteriophage with sequence `AACGCTACTA...GGATGTT`↻ (*NC_003287.2*$_{\text{GB}}$ and *V00604.2*$_{\text{GB}}$). Circular RNA (circRNA) are naturally occurring circular RNA formed from mRNA through a process called back-splicing [7], where a downstream splice donor site is joined to an upstream splice acceptor site. The *MW729338.1*$_{\text{GB}}$ represents an 840-nucleotide circRNA from Culex pipiens (northern house mosquito). Examples of circular proteins are `A`↻, `QSA`↻, and `QSALTQPASV`↻. A naturally occurring circular single-stranded amino-acid sequence is the 21 amino-acid long E. coli peptide Microcin J25 (*AAD28494.1*$_{\text{GB}}$ at positions 38–58) with sequence `GGAGHVPEYFVGIGTPISFYG`↻. This circular peptide is also referred to as `VGIGTPISFYGGGAGHVPEYF`↻, e.g. Blond et al. (1999) [8] and Trabi and Craik (2002) [9].

Because there is no beginning and end of a circular sequence, there is also no unique representation of a circular sequence without further constraints. both `AT`↻ and `TA`↻ represents the same circular sequence. Similarly, `GATTACA`↻, `ATTACAG`↻, `TTACAGA`↻, `TACAGAT`↻, `ACAGATT`↻, `CAGATTA`↻, and `AGATTAC`↻ are all the same circular sequence. In general, there are n different representations of the same circular single-stranded sequence of length n.

The non-uniqueness of these representations complicates how to store a circular sequence in databases, how to communicate it electronically, and how to present it in text. Similarly, if we naively would calculate the original SEGUID checksum for the above representations, we would get different checksums despite them representing the

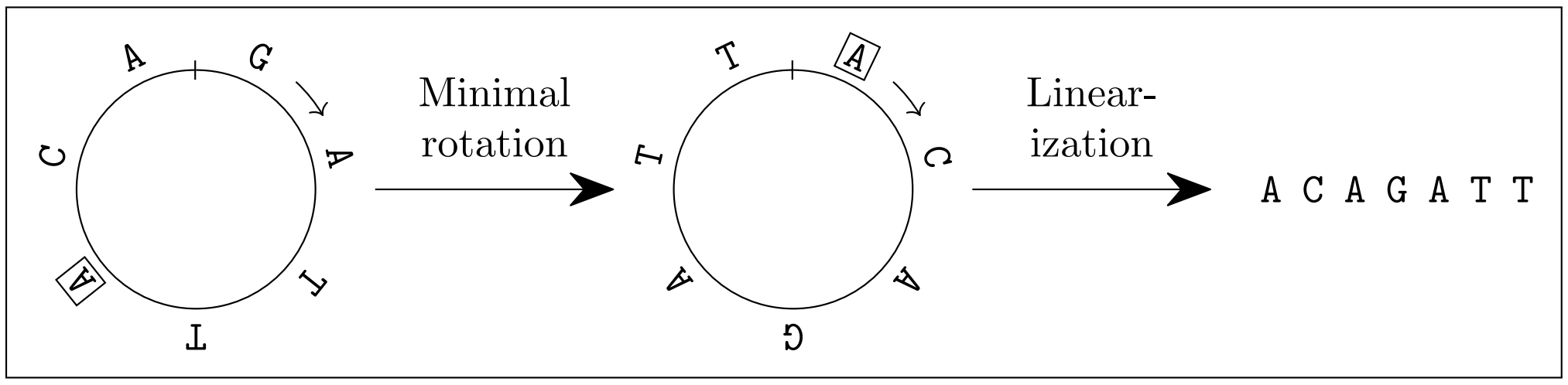

Fig 4. The SEGUID v2 steps for identifying the canonical representation of a circular, single-stranded DNA sequence. The input sequence `GATTACA`↻ (left) is one of several equivalent, non-normalized representations of the same circular sequence. Among all seven possible rotations, there is a minimal rotation, which for this sequence is `ACAGATT`↻ (middle). The linear representation of the minimal rotation is `ACAGATT` (right). It is from this linear representation that the SEGUID v2 checksum is calculated.

same circular sequence.

To address the above uniqueness problem, we need to identify a single unique representation among all possible representations part of the same equivalent class. Analogously to how we addressed the uniqueness problem for double-stranded sequences, a natural choice is to define the unique representation to be the sequence with a rotation that has the smallest lexicographical value. For example, consider the above circular sequence `GATTACA`↻ with seven equivalent representations. We can order the seven possible rotations as `ACAGATT` < `AGATTAC` < `ATTACAG` < `CAGATTA` < `GATTACA` < `TACAGAT` < `TTACAGA`. The minimal lexicographical rotation of `GATTACA`↻ is therefore `ACAGATT` (Fig 4). The minimal rotation can be found in linear time [10].

Circular double-stranded sequences

A circular double-stranded sequences is similar to a circular single-stranded sequences, but with a second complementary strand. Examples of circular double-stranded DNA sequences are `C`↔`G`↻, `AT`↔`AT`↻, and `TATGCCAA`↔`TTGGCATA`↻. A real-world example is the 2,686-basepair circular double-stranded DNA pUC19 cloning vector `TCGC...CGTC`↔`GACG...GCGA`↻ (*M77789.2*$_{\text{GB}}$ and *L09137.2*$_{\text{GB}}$).

Similarly to the single-stranded cases, there are multiple representations for circular double-stranded sequences. For example, the eight rotational variants `TATGCCAA`↔`TTGGCATA`↻, `ATGCCAAT`↔`ATTGGCAT`↻, `TGCCAATA`↔`TATTGGCA`↻, ..., `ATATGCCA`↔`TGGCATAT`↻ represent the same eight-basepair circular dsDNA, but so does the eight complementary counterparts `TTGGCATA`↔`TATGCCAA`↻, `ATTGGCAT`↔`ATGCCAAT`↻, `TATTGGCA`↔`TGCCAATA`↻, ..., `TGGCATAT`↔`ATATGCCA`↻. In general, there are $2 \cdot n$ different representations of the same circular double-stranded sequence of length n. We define the unique representation as the smallest of the minimal lexicographical rotations of the two strands. For instance, `AATATGCC`↔`GGCATATT`↻ is the minimal for one of original representation, and `ATATTGGC`↔`GCCAATAT`↻ for the complementary duality. Between these two, `AATATGCC`↔`GGCATATT`↻ is the lexicographically smallest, because `AATATGCC`↔`GGCATATT`↻ < `ATATTGGC`↔`GCCAATAT`↻. Thus, the canonical representation of the circular double-stranded sequence `TATGCCAA`↔`TTGGCATA`↻ is `AATATGCC`↔`GGCATATT`↻ (Fig 5). Note, it is sufficient to compare only `AATATGCC` < `ATATTGGC` to identify `AATATGCC`↔`GGCATATT`↻ to be the lexicographically minimal representation. This is true also for non-bijective complementary alphabets.

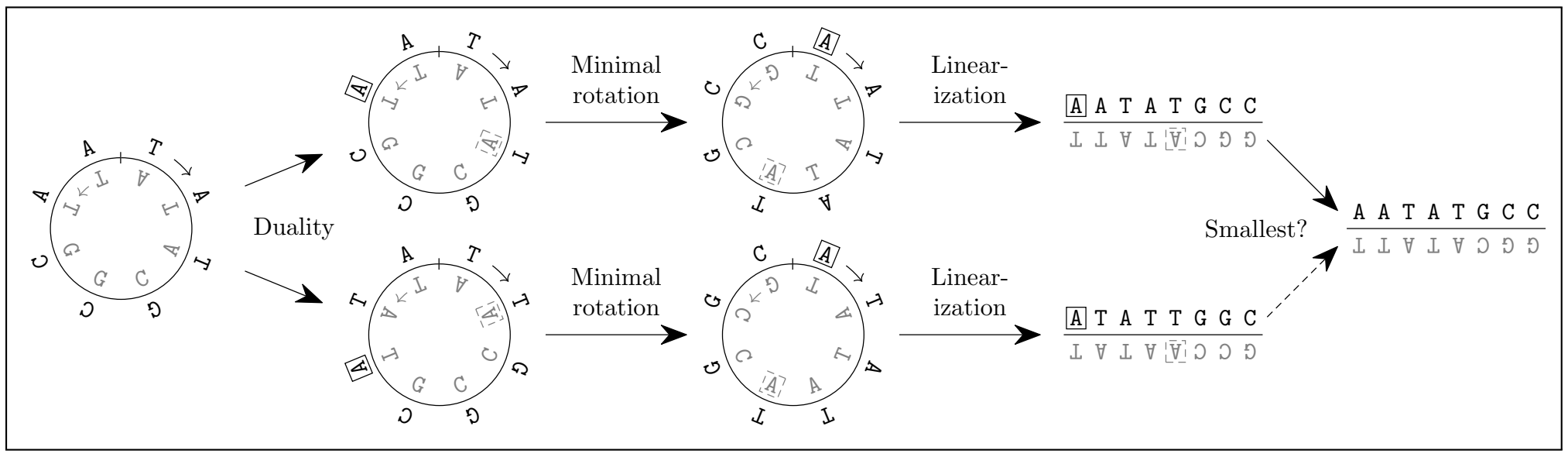

Fig 5. The SEGUID v2 steps for identifying the canonical representation of a circular, double-stranded DNA sequence. The input sequence `TATGCCAA`↩`TTGGCATA`↻ (left, and top left) is one of several equivalent, non-normalized representations of the same circular sequence. This double-stranded sequence has the dual complementary representation `TTGGCATA`↩`TATGCCAA`↻ (bottom left). The minimal rotations for these dual representations are `AATATGCC`↩`GGCATATT`↻ (top middle) and `ATATTGGC`↩`GCCAATAT`↻ (bottom middle). The smallest of the two linear minimal-rotated representations (top right and bottom right), is `AATATGCC`↩`GGCATATT` (right). It is from this linear representation that the SEGUID v2 checksum is calculated.

Hash function (SHA-1)

SEGUID v2 supports linear and circular, single- and double-stranded sequences. As shown above, circular sequences are transformed to unique, linear representations based on minimal rotations and lexicographic ordering. Thus, regardless of input sequence, the hash is always calculated on a linear representation. To apply the hash function to these representations, we need to coerce them to single strings[6]. There are two types of linear representations we have to consider; the single-stranded one and the double-stranded one. A linear single-stranded sequence can be coerced to a string as-is, e.g. the sequence `GATTACA` becomes the string `"GATTACA"`. For a linear double-stranded sequence, we chose to coerce it to a string by concatenating the 5′→3′ Watson sequence and the 5′→3′ Crick sequences with a semicolon (`;`). For example, the blunt, for linear dsDNA sequence `GATTACA`↩`TGTAATC`, the hashed string is `"GATTACA;TGTAATC"`. Similarly, for the staggered, linear dsDNA sequence `--TTACA`↩`-GTAATC` the hashed string is `"--TTACA;-GTAATC"`.

The original SEGUID method uses **SHA-1** ~~(Secure Hash Algorithm 1)~~ to generate a 160-bit hash from the input sequence. As argued in Babnigg and Giometti (2006) [4], **SHA-1** is less sensitive to collisions compared to **CRC-64** and sequence checksum collisions are very unlikely when using **SHA-1**. Another, popular hash function is the Message Digest Algorithm 5 (**MD5**) [11], which produces a 128-bit hash. The UniProt database uses the **MD5** checksum. Although we are not aware of any collisions for biological data, it has been shown [12] that **MD5** collisions can be created artificially. Although we have not yet observed a **SHA-1** collision, one could argue that we should "upgrade" to a **SHA-2** algorithm to further lower the risk for collisions. On the other hand, that comes with a cost of producing longer checksums. For example, the popular and widely available **SHA-256** algorithm produces a 256-bit long hash, which is 60% longer than a **SHA-1** hash, which in turn would lengthen the final checksum the same amount. There are downsides to having long checksums. For instance, they may be too long to be used for metadata headers in some sequence file formats, they risk being line-wrapped in communications such as email threads, and they add to the

[6] Technically, hash functions operates on raw bytes. To calculate a hash for a string, we ensure to use ASCII strings such that each characters uniquely maps to an integer byte.

storage size for very-large lookup tables. Moreover, some file systems have a limit on how long a canonical pathname may be, e.g. on Microsoft Windows the default pathname-length limit is 256 characters. A very long checksums in a directory or file name, in an already deeply nested directory structure, may exceed this limit. An alternative to **SHA-256** is **SHA-224**, which results in "only" a 40% longer checksum and might therefore be a hash-algorithm candidate. Unfortunately, implementations of **SHA-224** are less common across programming languages than **SHA-1**, **SHA-256**, and **SHA-512**. Yet another alternative is **SHA-512/224**, which produces a 224-bit truncated hash based on the **SHA-512** algorithm [5]. The SHA specification support **SHA-512/t** for other values of **t**, e.g. **SHA-512/160** would produce a hash of the same length as **SHA-1**. Readily available implementations of these truncated alternatives are even less common than **SHA-224**. An alternative approach to **SHA-512/t** was proposed by Hart and Prlić (2020) [13], where they discard the last 320 bits of the 512-bit **SHA-512** hash to produce a 192-bit hash. We contemplated this approach, but we decided against it as we do not know if it is statistically sound or valid for our needs.

Lastly, Stevens et al. (2017) [14] and Leurent and Peyrin (2020) [15] demonstrated that it is feasible to artificially construct **SHA-1** clashes on modern computers. Because of this, **SHA-1** is no longer considered safe from a cryptography point of view. For instance, the U.S. National Institute of Standards and Technology (NIST) "*recommends that federal agencies transition away from SHA-1 for all applications as soon as possible. Federal agencies should use SHA-2 or SHA-3 as an alternative to SHA-1*" [16]. The **SHA-2** and **SHA-3** family of hash functions produce longer hashes, making it harder to construct collisions. That said, sequence checksums are used for efficient storage, communication, and lookup of sequences - not to secure them cryptographically. Arguing that the checksum of a full sequence can guarantee that the full sequence has not be compromised is problematic and risky. To achieve that, cryptographical protocols based on, for instance, public-and-private keys should be used, which is a problem beyond sequence checksums. We find no reason to incorporate such features in SEGUID v2, because there already exist well-established, readily available protocols for encrypted communication and storage.

For these reasons, we choose to use **SHA-1** also for SEGUID v2.

Encoding (Base64url)

A hash, by itself, is sufficient to uniquely identify a sequence. However, hashes, such as **SHA-1**, are very large integers, larger than can easily be represented in common programming languages, which makes them complicated to coerce as-is to and from textual representations. Encoding is a technique to turn an internal binary representation to a string, preferably in a compact way. Different encoding algorithms have different "compression" ratios (Table 1).

For instance, the **SHA-1** hash, which is a 160-bit integer, or equivalently a 20-byte integer, could be encoded to a string of length 160 comprising symbols zero (`0`) and one (`1`). Assuming an 8-bit ASCII string, this encoding is 800% larger than the internal 20 bytes. That would be an inefficient way to represent the hash. A more compact, and commonly used encoding is the hexadecimal representation, which comprise an 16-character alphabet (`0-9` and `a-f`). In this representation, a **SHA-1** hash is of length 40 characters; one for every four ($\log_2 16$) bits, or two for each byte. To achieve a more compact checksum, a larger set of symbols is needed. The **Base64** encoding [17] uses a 64-character alphabet (`A-Z`, `a-z`, `0-9`, `+`, and `/`). With this, each character can encode six ($\log_2 64$) bits, i.e. a 160 bits can be represented by $\lceil 160/6 \rceil = 27$ characters.[7]

[7]Technically, **Base64** encoding requires a multiple of four symbols. To meet this requirement, the

Description	Value	Length
**SHA-1** (binary)	$10011010110110\ldots0010100100_2$	160 bits
**SHA-1**+Base16	`9adaef6edbb0afafcca01c6fb66e01129bfe8ca4`	40 symbols
**SHA-1**+Base64	`mtrvbtuwr6/MoBxvtm4BEpv+jKQ=`	28 symbols
**SHA-1**+Base64url	`mtrvbtuwr6_MoBxvtm4BEpv-jKQ=`	28 symbols
SEGUID v1	`mtrvbtuwr6/MoBxvtm4BEpv+jKQ`	27 symbols
SEGUID v2	`mtrvbtuwr6_MoBxvtm4BEpv-jKQ`	27 symbols

Table 1. Examples of different encodings of a SHA-1 hash. The **SHA-1** hash was generated based on the sequence string `ACAGATT`. The **Base64** specification requires that the output comprise a multiple of four symbols, and if not, the output should be padded with equal signs (`=`) at the end. Because of this, and a **Base64** encoding of a **SHA-1** hash will always have a single, non-informative padding symbol at the end. This padding symbol is excluded in the SEGUID checksums.

The original SEGUID method uses **Base64**, modulo the mandatory, non-informative padding symbol, to encode the **SHA-1** hash, resulting in a 27-character checksum. This was chosen to get a compact representation of the hash, while still being text-friendly. One problem with a **Base64**-based checksum is that it cannot be used as part of a filename, because of the forward slash (`/`), which is used to represent directories on most file systems. For similar reason, it cannot be used as-is as part of a Uniform Resource Locator (URL), where symbols `/` and `+` have special meaning. In order to use a SEGUID checksum in a URL, these symbols need to be re-encoded. To address these needs, a filename- and URL-safe variant of **Base64** is the **Base64url** encoding [17]. It substitutes forward slashes (`/`) and plus symbols (`+`) with underscores (`_`) and minus symbols (`-`). The **Base64url** algorithm is linear ($O(n)$) in the number of bits, which is always 160 bits when using **SHA-1**.

In contrast to the original SEGUID, for SEGUID v2, we choose to use the **Base64url** encoding. For example, the linear single-stranded DNA sequence `ACAGATT` yields the SEGUID v1 checksum `mtrvbtuwr6/MoBxvtm4BEpv+jKQ`, while the SEGUID v2 checksum is `mtrvbtuwr6_MoBxvtm4BEpv-jKQ` (Table 1).

SEGUID v2 checksums have a prefix

In order to preserve what type of information is encoded in the checksum, SEGUID v2 checksums are by default prefixed with either `lsseguid`, `csseguid`, `ldseguid`, or `cdseguid`, followed by an equal sign (`=`). For example, `lsseguid=IQiZThf2zKn_I1KtqStlEdsHYDQ` indicates a SEGUID v2 checksum for a linear single-strand sequence.

The prefix helps to distinguish SEGUID v2 checksums from other types of 27-character checksum strings. It also conveys type of sequence the checksum represents. We have found that the latter helps users to detects mistakes sooner in case they selected the incorrect sequence type for SEGUID v2.

Another advantage of the prefix is that it eliminates the risk of getting checksums with all digits, which some software tools might parse as numeric values. For example, Microsoft Excel and LibreOffice Calc will coerce such all-digit strings to numerics, but with loss of precision. If such a numeric value is coerced back to a string, it will comprise of the first 15-16 digits followed by all zeros. We observe the same problem with higher-level programming functions (e.g. in R and Python) that reads data from file risk reading all-digit columns as loss-of-precision numeric values or overflowed integer values.

27-character string representing a **SHA-1** hash should be padded with a special symbol (`=`) at the very end.

SEGUID Short ID

For practical purposes, SEGUID v2 defines a short form of its checksum. Specifically, a SEGUID Short ID comprise the first six (6) characters of the checksum without the prefix. For example, the Short ID of the SEGUID v2 checksum `lsseguid=IQiZThf2zKn_I1KtqStlEdsHYDQ` is `IQiZTh`.

While we designed the SEGUID v2 checksum to be text-friendly, we designed the Short ID to be human-friendly. The SEGUID Short ID provides a compact way to reference a specific sequence. The short length makes it easy to communicate in both written and spoken form, and they allow for quick manual comparisons. It is also feasible to memorize and recognize, especially in projects that have a few sequences that are commonly used.

The risk for collisions among Short ID is still relatively small, especially for any given project working with a limited set of sequences. Theoretically, a six-character Short ID can encode $2^{36} = 1,073,741,824$ different sequences[8].

Supported sequence types and alphabets

Alphabet	#Symbols	Complementary	Definition
DNA	4	Bijective	Nucleotides `A`↔`T`, `C`↔`G`.
Extended DNA	15	Bijective	DNA + `K`↔`M`, `R`↔`Y`, `S`↔`S`, `W`↔`W`, `B`↔`V`, `D`↔`H`, `N`↔`N`.
RNA	4	Bijective	Nucleotides `A`↔`U`, `C`↔`G`.
Extended RNA	15	Bijective	RNA + `K`↔`M`, `R`↔`Y`, `S`↔`S`, `W`↔`W`, `B`↔`V`, `D`↔`H`, `N`↔`N`.
Protein	20	No	Amino acids `A`, `C`, `D`, `E`, `F`, `G`, `H`, `I`, `K`, `L`, `M`, `N`, `P`, `Q`, `R`, `S`, `T`, `V`, `W`, `Y`, plus `U`, `O`.
Extended protein	26	No	Protein + `B`, `J`, `Z`, `X`.
Custom	1 – 60	Optional	Digits (`0-9`), upper-case letters (`A-Z`), lower-case letters (`a-z`).

Table 2. Alphabets supported by SEGUID v2. SEGUID v2 has built-in support for DNA, RNA, and proteins. These alphabets comprise upper-case letters only. IUPAC is a standard to represent DNA, RNA, and amino-acid sequences with an "extended" set of symbols [6, 18]. Custom alphabets, complementary or not, are also supported. The concept of complementarity does not apply to proteins. Proteins are effectively single-stranded sequences, meaning only the single-stranded SEGUID v2 methods apply to them. Amino-acid symbols `J`, `O`, and `U` are not part of the IUPAC standard.

The SEGUID v2 algorithm is designed to support DNA, RNA, and proteins, but also sequences with a custom alphabet (Table 2). Formally, the input sequence must not include symbols outside of the declared case-sensitive alphabet. By requiring case sensitivity, we allow for a richer set of custom alphabets, but we also avoid the risk of introducing non-deterministic behavior from, for instance, automatically coercing lower-case letters to upper-case ones. In addition, we limit alphabets to digits, and upper-, and lower-case letters. This is to assure a well-defined lexicographic ordering of symbols (per the 7-bit ASCII character set). Importantly, there are reserved

[8]The Short ID holds 6 symbols $\cdot \log_2 64$ bits/symbol = 36 bits (22.5%) of the original 160 bits of the **SHA-1** hash.

symbols that must never be part of the alphabet. Specifically, the dash symbol (`-`), the semicolon (`;`), and the newline (`\n`) are reserved symbols.

Results

SEGUID v2 is designed to be robust against mistakes in order to prevent incorrect checksums from propagating in the scientific process. For instance, part of the requirements is that the implementation should validate that correctness of the input sequence, e.g. all symbols are part of the alphabet and double-stranded sequences are correctly complementary. At the same time as being strict, SEGUID v2 is designed to be flexible such that it can support existing and future sequences not already anticipated. For example, in Viner et al. (2024) [19], the authors define an expanded epigenetic DNA alphabet (Table 3) that encodes also known modifications to cytosine (`C`). Using this alphabet, methylation motifs can be represented as, for instance, `TTGmGCAA`, `AT2hAAAT`, and `TGCm1m1`. Their complementary counterparts are `TTGC1CAA`, `ATTT2hAT`, and `m1m1GCA`. They also define an "extended" version of this alphabet, with additional complementary symbol pairs. Both of these alphabets can be used with SEGUID v2 by defining them as custom alphabets.

Alphabet	#Symbols	Complementary	Definition
Expanded epigenetic alphabet	12	Bijective	DNA + `m`↔`1`, `h`↔`2`, `f`↔`3`, `c`↔`4`.
Ambiguous expanded epigenetic alphabet	20	Bijective	Expanded epigenetic alphabet + `w`↔`6`, `x`↔`7`, `y`↔`8`, `z`↔`9`.
DNA with Uracil	5	Non-bijective	`A`↔`T`, `A`↔`U`, `C`↔`G`.

Table 3. Custom alphabets with SEGUID v2. (i) The expanded epigenetic alphabet, defined in Table 1 of Viner et al. (2024) [19], includes known modifications to cytosine (`C`) and symbols for each guanine (`G`) complementary to a modified nucleobase. (ii) The "ambiguous" version of this alphabet, defined in Table 2 of Viner et al. (2024) [19], addresses the case when the knowledge on some of nucleotides is incomplete. (iii) Uracil (`U`) may occur naturally in DNA, which in case adenine (`A`) is complementary to either thymine (`T`) or uracil (`U`). This can be represented by a non-bijective complementary alphabet.

Another example arise from the fact that uracil (`U`) may occur naturally in DNA, e.g. phage DNA from dUTPase, uracil-DNA glycosylase (dut ung) mutant hosts [20], as an apoptosis inducer during Holometabola development [21], and as a mean for somatic hypermutation resulting from U:G mismatch repair during vertebrate antibody development [22]. See Fig 6 for an illustration.

This also occurs in the USER™ Uracil excision cloning protocol [23]. In these cases, adenine (`A`) may bind to either thymine (`T`) or uracil (`U`), which can be described by a non-bijective complementary alphabet (Table 3).

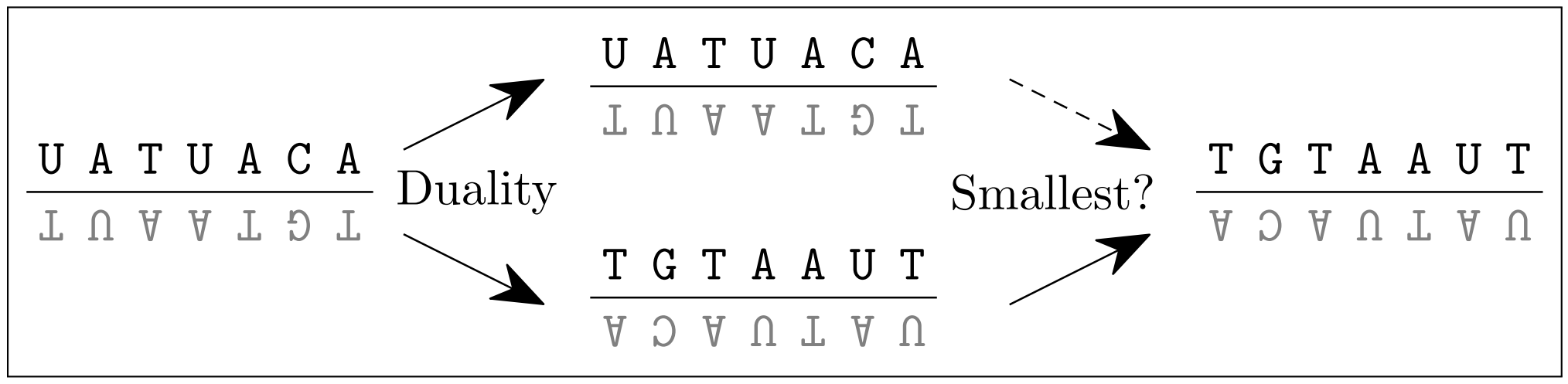

Fig 6. The SEGUID v2 algorithm on double-stranded sequence of DNA with uracil. The SEGUID v2 algorithm supports non-bijective complementary alphabets such as DNA with uracil (`U`). The input sequence `UATUACA`⟵`TGTAAUT` (left and top middle) comprise basepairs `A`↔`T` and `A`↔`U`. It has a dual, complementary representation `TGTAAUT`⟵`UATUACA` (bottom middle), which is also the lexicographically smallest (right).

Implementation and availability

Minimal, core software implementations

The SEGUID v2 algorithm, together with original SEGUID algorithm, is implemented and distributed as libre, open-source software under the MIT license. We provide small, self-contained implementations in JavaScript [24], Python [25], R [26], and Tcl [27] that have a minimal set of dependencies. For JavaScript, Python and R, we provide packages named **seguid**, which are available on npm (node package manager), PyPi (Python Package Index), and CRAN (Comprehensive R Archive Network), respectively. These packages implement a standardized application protocol interface (API), e.g. `csseguid("GATTACA")`. They also provide a command-line interface (CLI), e.g.

```
$ npx seguid --type=lsseguid <<< 'GATTACA'
lsseguid=tp2jzeCM2e3W4yxtrrx09CMKa_8

$ python -m seguid --type=cdseguid <<< 'TATGCCAA;TTGGCATA'
cdseguid=dUxN7YQyVInv3oDcvz8ByupL44A

$ Rscript -e seguid::seguid --type=csseguid <<< 'GATTACA'
csseguid=mtrvbtuwr6_MoBxvtm4BEpv-jKQ

$ tclsh seguid --type=ldseguid <<< 'TATGCCAA;TTGGCATA'
ldseguid=p88RYs41n0NTej4htM1fJAWI1ME
```

Documentation, examples, and online WebAssembly-based [28] demos are available on `https://www.seguid.org`. Support for additional programming languages are planned, specifically C++ and Rust, and will be made available via the SEGUID website.

Validation and testing

It is essential that any implementation of the SEGUID v2 checksum algorithm is correct and comply with the SEGUID v2 specifications as described here. For instance, we do not want to incorrectly produced checksums to be used in production, appear in publications, or entering shared databases. We provide a rich set of unit and redundancy tests (see SEGUID website) to lower the risk for this to ever occur. These

tests assert correctness, and identical behavior and results regardless of which programming language was used for the implementation. All new implementations must adhere to these tests in order to be considered SEGUID v2 compliant. These tests run on all releases and on a nightly basis. This test framework is designed to make it as simple as possible for a third-party contributor to run these tests. We use continuous-integration testing to validate all known implementations.

Higher-level implementations

The main purpose of the above **seguid** packages is to provide a minimal, language-agnostic API of the SEGUID v2 algorithm that can be imported in higher-level implementations. The scope of these packages will be kept at a minimum to avoid feature creep of both of the API and the SEGUID v2 algorithm. This way, the packages also serve as reference implementations and help lower the threshold for implementing the API in other languages.

For these reasons, we consider tasks like coercing a DNA sequence in lower-case letters to upper-case letters, trimming strings, and converting a sequence to its complementary counterpart to be out of scope. Such features have to be implemented by other tools and packages, e.g. Python packages **pydna** [29] and **Biopython** [30, 31]. For example, **pydna** implements the SEGUID v2 methods for the `Dseqrecord` class, which holds double-stranded DNA. It also provides a rich set of utility functions for operating on DNA and RNA sequences. **Biopython** already implements the original SEGUID method and we anticipate it will also provide SEGUID v2 functionalities in a future release.

Graphical user interfaces (GUIs)

Some users are not comfortable with the provided command-line interfaces, or inexperienced with the supported programming languages. In order to make SEGUID v2 accessible to these users it is important the method is also available through a Graphical User Interface (GUI). A readily available GUI will boost the adoption of SEGUID v2 to a broader audience as it also caters to users of varying expertise levels. Below are two GUIs that can be used for calculating SEGUID checksums.

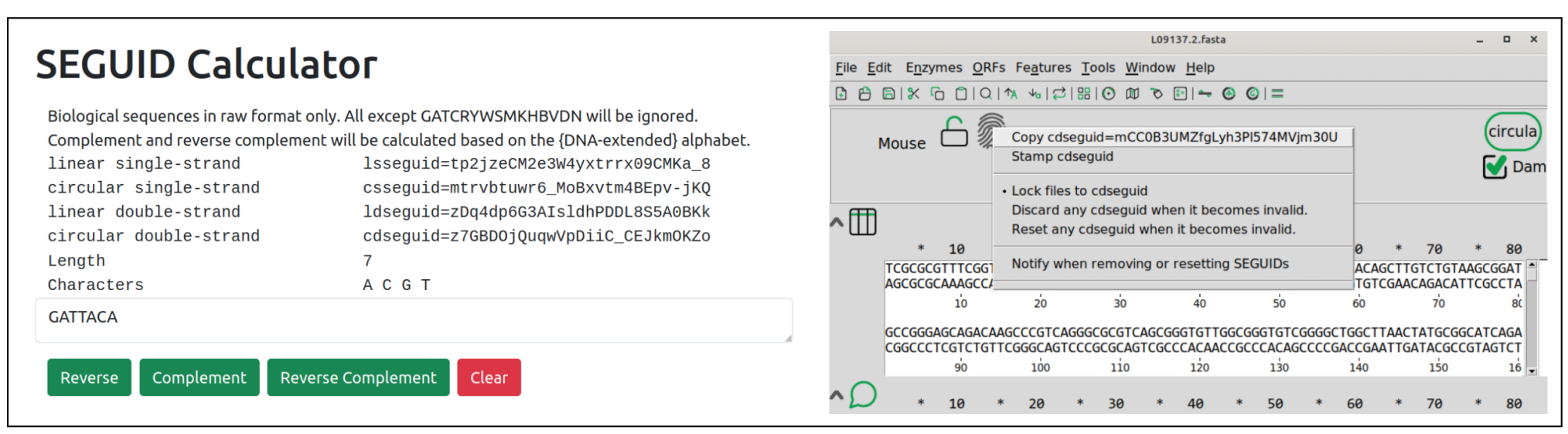

Fig 7. GUI implementations of SEGUID checksums. Two screenshots of GUI tools for calculating SEGUID v2 checksums. The **SEGUID Calculator** tool (left) calculates the different types of SEGUID v2 checksums based on the Watson sequence as it is being entered. This a JavaScript-based implementation that fully runs within the web browser. The A plasmid Editor (**ApE**) tool (right) is specifically designed for double-stranded DNA. Because of this, it is sufficient to enter the Watson sequence, from which the Crick sequence is automatically inferred.

The **SEGUID Calculator** (Fig 7) is a browser-based GUI for the SEGUID v2 algorithm available via the SEGUID website. It is implemented in JavaScript and

allows for computations such that entered data never leaves the web browser or the local computer. This type of instant interactive feedback enhances understanding of how the checksum works.

The popular, freely available **ApE** (A plasmid Editor) [32] allows calculating and stamping dsDNA sequences with SEGUID v2 checksums (Fig 7). **ApE** is built on our Tcl implementation and available from the Jorgensen Lab website[9].

Applications

SEGUID checksums can be used to map sequence metadata both within and across biological databases. For example, by indexing different types of identifiers based on SEGUID checksums, the SEGUID checksum can serve as the common denominator across heterogeneous databases. For example, this technique was used at the Argonne National Laboratory to build a real-time, interactive search engine for protein annotation data collected from multiple resources. For motivations similar to those of the Short ID, the search engine was designed to list matching candidates as soon as the user had typed three letters (Fig 8).

Analogously to how SEGUID checksums can be used for quick lookup of sequence data via database tools, they can be used for navigating and looking up sequence data hosted directly on the file system. For example, sequence-annotation data files of different kinds can be grouped in folders named as the corresponding SEGUID v2 checksums, or the individual files can comprise the checksum.

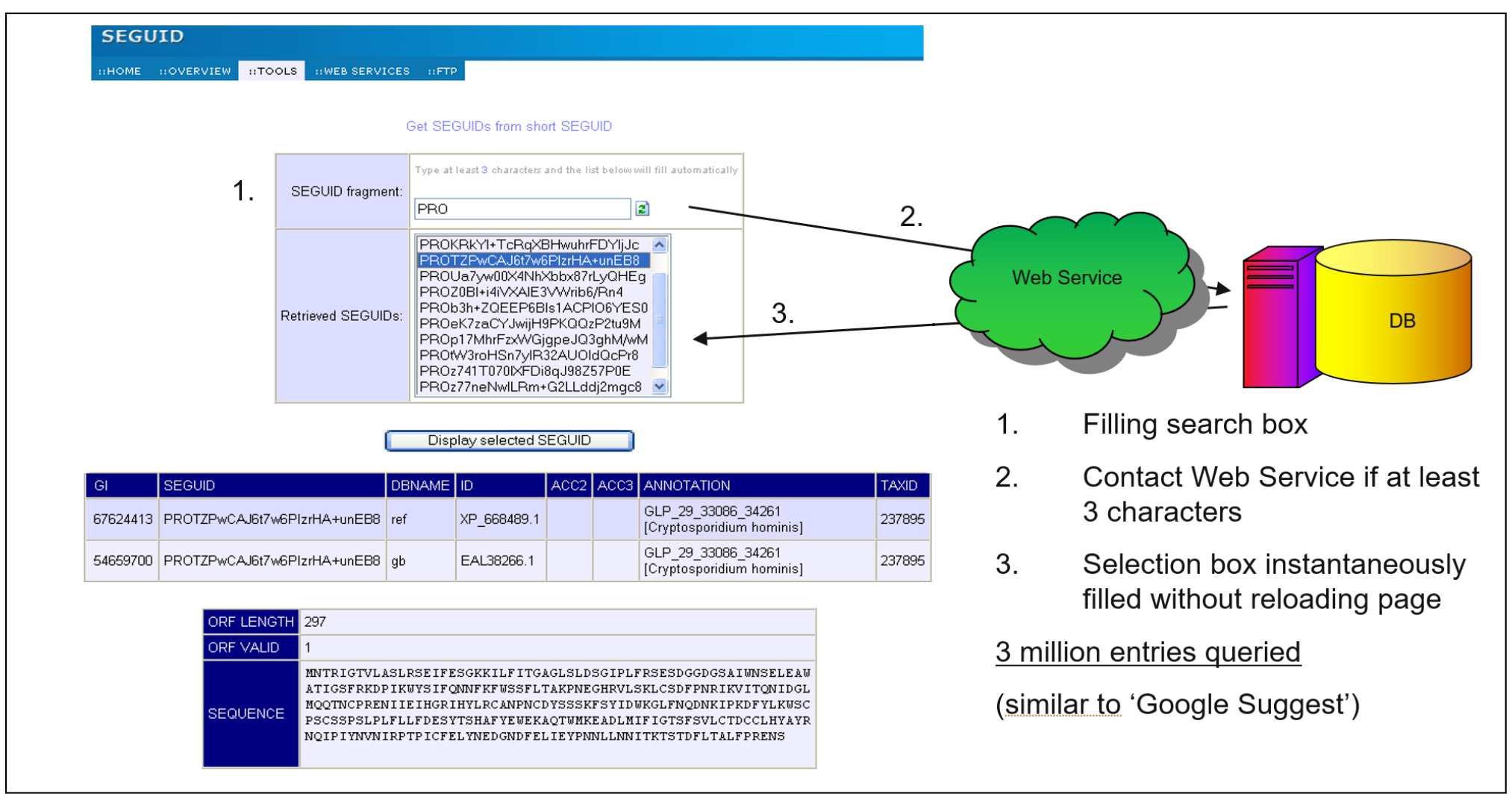

Fig 8. Type-ahead database search based on partial SEGUID checksums. Illustration of a how partial SEGUID checksums can be used to autocomplete searches as you type. In this example, the user enters `PRO` and the Ajax-based web interface instantaneously returns all SEGUID checksums starting with those characters. Sequence data is pulled from multiple databases concurrently for entries matching the selected SEGUID checksum.

The **ApE** tool and the **pydna** package can stamp and save sequence files in GenBank format[10] where the checksum is saved in the `COMMENT` section of the GenBank file (Fig 9). An existing checksum can be verified by each tool.

[9] `https://jorgensen.biology.utah.edu/wayned/ape`
[10] `https://www.ncbi.nlm.nih.gov/genbank/samplerecord/`

ApE

```
LOCUS       New_DNA                    7 bp    DNA     circular     11-MAY-2023
DEFINITION  .
ACCESSION
VERSION
SOURCE      .
  ORGANISM  .
COMMENT     ApEinfo:methylated:0
COMMENT     ApEinfo:csseguid=mtrvbtuwr6_MoBxvtm4BEpv-jKQ
ORIGIN
        1 gattaca
//
```

pydna

```
LOCUS       name                       7 bp    DNA     circular UNK 01-JAN-1980
DEFINITION  description.
ACCESSION   id
VERSION     id
KEYWORDS    .
SOURCE      .
  ORGANISM  .
COMMENT     pydna csseguid=mtrvbtuwr6_MoBxvtm4BEpv-jKQ 2023-10-13T10:11:54
FEATURES    Location/Qualifiers
ORIGIN
        1 gattaca
//
```

Fig 9. SEGUID checksums added to GenBank sequence files. The files were stamped with SEGUID checksum using the **ApE** tool (upper panel) or the **pydna** Python package (lower panel). In both cases, the checksum is added in the `COMMENT` field of the GenBank record. In addition, **pydna** appends an ISO-8601 timestamp after the checksum.

Cloning exercise

The plasmid vector pUC19 (2,686 bp, `cdseguid=mCC0B3UMZfgLyh3Pl574MVjm30U`) is a widely used cloning vector. The sequence is available from GenBank under the accession number `L09137.2`. Simulate the non-directional cloning of the dsDNA fragment below:

```
gatccATGCAATAAg
    gTACGTTATTcctag
```

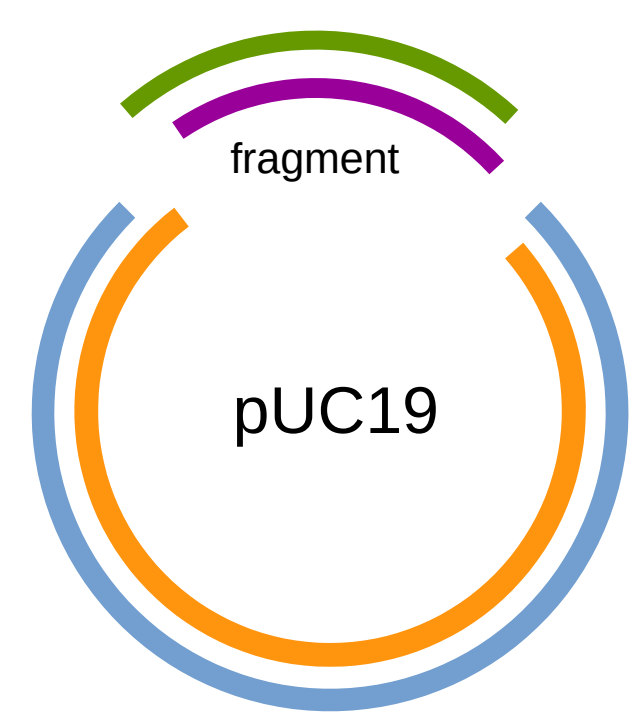

pUC19 was linearized with *Bam*HI. Enter the SEGUID checksums for the two resulting vectors (pUC19_frag_A and pUC19_frag_B) in the **shared spreadsheet** next to your name.

Sequences	Short ID
pUC19_frag_A	**CzM-IG**
pUC19_frag_B	**S4WZki**

Fig 10. Cloning exercise. Students, in undergraduate courses at the Department of Biology of the University of Minho, are given a cloning simulation problem (top) that will result in two vectors. They are given the Short IDs of the correct vectors for them to self-correct before filing the results.

A common use of the SEGUID checksum is in undergraduate teaching of practical

molecular biology in the Department of Biology at the University of Minho. An example problem is depicted in Fig 10. The students are given a task, typically to simulate a sub-cloning problem on their computers. This kind of problem has a unique correct answer, making it possible to calculate checksums for the correct solution beforehand. The problem of assessing the students is simplified by the students access to a local or web-based SEGUID-capable software, e.g. **SEGUID Calculator**. Giving access to a partial checksum for the correct answer can help the students to rapidly determine whether their procedures are incorrect. We have found this approach practical to teach in-silico cloning to over 60 students at a time. Overall, it lowers the number of mistakes filed and the efforts on reviewing homework assignments.

Discussions

The SEGUID v2 method produces checksums for various types of biological sequences, including, but not limited to, DNA, RNA, and proteins. The method is invariant to orientation of double-stranded sequences, and invariant to rotation for circular sequences. In contrast to other methods (e.g. Bouras et al. (2024) [33]), SEGUID v2 is also invariant to genome and does not require the input to comprise known gene sequences.

The SEGUID v2 checksum is capable of providing the scientific community with stable keys for the many thousands of plasmids created in the process of designing new recombinant organisms or synthetic biological constructs. Important plasmid sequences can be stored in a distributed fashion, without the need for expensive centralized infrastructure. The integrity of sequences distributed among members of the scientific community can easily be verified. Using SEGUID v2 makes it trivial to confirm that members of a research groups are all using the same sequence for a particular DNA fragment containing a genetic part. The SEGUID v2 checksum would also enable a global reference service for circular DNA, such as the registry provided by EMBL-EBI at identifiers.org. Since the checksum is already implemented in the popular DNA editor ApE, we believe this will encourage wider adoption by both researchers and web-facing applications.

Acknowledgments

We would like to express our gratitude to the undergraduate students of Applied Biology and Biological Engineering at the University of Minho who have helped us to develop this work over the years.

References

1. Kamens J. The Addgene repository: an international nonprofit plasmid and data resource. Nucleic Acids Research. 2014 11;43(D1):D1152-7. Available from: `https://doi.org/10.1093/nar/gku893`. doi:10.1093/nar/gku893.

2. Kamens J. Addgene: Making Materials Sharing "Science As Usual". PLOS Biology. 2014 11;12(11):1-5. Available from: `https://doi.org/10.1371/journal.pbio.1001991`. doi:10.1371/journal.pbio.1001991.

3. H PW, P FB, A TS, T VW. In: Cyclic redundancy and other checksums. 2nd ed. Cambridge University Press; 1993. p. 896-902.

4. Babnigg G, Giometti CS. A database of unique protein sequence identifiers for proteome studies. Proteomics. 2006 Aug;6(16):4514-22. Available from: `https://onlinelibrary.wiley.com/doi/10.1002/pmic.200600032`. doi:10.1002/pmic.200600032.

5. FIPS PUB 180-4. Secure Hash Standard (SHS). Information Technology Laboratory, National Institute of Standards and Technology, Gaithersburg, MD 20899-8900: National Institute of Standards and Technology (NIST, U.S.); 2015. Federal Information Processing Standards Publication (FIPS PUB 180-4). doi:10.6028/nist.fips.180-4.

6. IUPAC-IUB Commission on Biochemical Nomenclature. Abbreviations and symbols for nucleic acids, polynucleotides, and their constituents. Biochemistry. 1970;9(20):4022-7. Available from: `https://doi.org/10.1021/bi00822a023`. arXiv:https://doi.org/10.1021/bi00822a023. doi:10.1021/bi00822a023.

7. Jeck WR, Sorrentino JA, Wang K, Slevin MK, Burd CE, Liu J, et al. Circular RNAs are abundant, conserved, and associated with ALU repeats. RNA. 2013 Feb;19(2):141-57.

8. Blond A, Péduzzi J, Goulard C, Chiuchiolo MJ, Barthélémy M, Prigent Y, et al. The cyclic structure of microcin J25, a 21-residue peptide antibiotic from Escherichia coli. European Journal of Biochemistry. 1999;259(3):747-56. Available from: `https://febs.onlinelibrary.wiley.com/doi/abs/10.1046/j.1432-1327.1999.00085.x`. arXiv:https://febs.onlinelibrary.wiley.com/doi/pdf/10.1046/j.1432-1327.1999.00085.x. doi:https://doi.org/10.1046/j.1432-1327.1999.00085.x.

9. Trabi M, Craik DJ. Circular proteins–no end in sight. Trends Biochem Sci. 2002 Mar;27(3):132-8.

10. Pierre Duval J. Factorizing words over an ordered alphabet. Journal of algorithms & computational technology. 1983 Dec;4(4):363-81. Available from: `https://www.sciencedirect.com/science/article/pii/0196677483900172`. doi:10.1016/0196-6774(83)90017-2.

11. Rivest RL. The MD5 Message-Digest Algorithm. RFC Editor; 1992. RFC 1321. Available from: `https://www.rfc-editor.org/info/rfc1321`. doi:10.17487/RFC1321.

12. Wang X, Yu H. How to Break MD5 and Other Hash Functions. In: Cramer R, editor. Advances in Cryptology – EUROCRYPT 2005. Berlin, Heidelberg: Springer Berlin Heidelberg; 2005. p. 19-35.

13. Hart RK, Prlić A. SeqRepo: A system for managing local collections of biological sequences. PLOS ONE. 2020;15(12):e0239883. doi:10.1371/journal.pone.0239883.

14. Stevens M, Bursztein E, Karpman P, Albertini A, Markov Y. The First Collision for Full SHA-1. In: Katz J, Shacham H, editors. Advances in Cryptology – CRYPTO 2017. Cham: Springer International Publishing; 2017. p. 570-96.

15. Leurent G, Peyrin T. SHA-1 is a Shambles: First Chosen-Prefix Collision on SHA-1 and Application to the PGP Web of Trust. In: 29th USENIX Security Symposium (USENIX Security 20). USENIX Association; 2020. p. 1839-56. Available from: `https://www.usenix.org/conference/usenixsecurity20/presentation/leurent`.

16. Computer Security Resource Center (CSRC). NIST Policy on Hash Functions; 2022. The U.S. National Institute of Standards and Technology. Available from: https://csrc.nist.gov/projects/hash-functions/nist-policy-on-hash-functions.

17. Josefsson S. The Base16, Base32, and Base64 Data Encodings; 2006. RFC 4648. Available from: https://www.rfc-editor.org/rfc/pdfrfc/rfc4648.txt.pdf. doi:10.17487/RFC4648.

18. IUPAC-IUB Commission on Biochemical Nomenclature. Nomenclature and Symbolism for Amino Acids and Peptides. European Journal of Biochemistry. 1984;138(1):9-37. Available from: https://febs.onlinelibrary.wiley.com/doi/abs/10.1111/j.1432-1033.1984.tb07877.x. arXiv:https://febs.onlinelibrary.wiley.com/doi/pdf/10.1111/j.1432-1033.1984.tb07877.x. doi:https://doi.org/10.1111/j.1432-1033.1984.tb07877.x.

19. Viner C, Ishak CA, Johnson J, Walker NJ, Shi H, Sjöberg-Herrera MK, et al. Modeling methyl-sensitive transcription factor motifs with an expanded epigenetic alphabet. Genome Biology. 2024 jan;25(1):11. Available from: https://doi.org/10.1186/s13059-023-03070-0. doi:10.1186/s13059-023-03070-0.

20. Wang Z, Mosbaugh DW. Uracil-DNA glycosylase inhibitor of bacteriophage PBS2: cloning and effects of expression of the inhibitor gene in Escherichia coli. Journal of Bacteriology. 1988;170(3):1082-91. Available from: https://journals.asm.org/doi/abs/10.1128/jb.170.3.1082-1091.1988. arXiv:https://journals.asm.org/doi/pdf/10.1128/jb.170.3.1082-1091.1988. doi:10.1128/jb.170.3.1082-1091.1988.

21. Muha V, Horváth A, Békési A, Pukáncsik M, Hodoscsek B, Merényi G, et al. Uracil-containing DNA in Drosophila: stability, stage-specific accumulation, and developmental involvement. PLoS genetics. 2012 Jun;8(6):e1002738. Available from: http://dx.doi.org/10.1371/journal.pgen.1002738. doi:10.1371/journal.pgen.1002738.

22. Muramatsu M, Kinoshita K, Fagarasan S, Yamada S, Shinkai Y, Honjo T. Class switch recombination and hypermutation require activation-induced cytidine deaminase (AID), a potential RNA editing enzyme. Cell. 2000 Sep;102(5):553-63. Available from: http://dx.doi.org/10.1016/s0092-8674(00)00078-7. doi:10.1016/s0092-8674(00)00078-7.

23. Geu-Flores F, Nour-Eldin HH, Nielsen MT, Halkier BA. USER fusion: a rapid and efficient method for simultaneous fusion and cloning of multiple PCR products. Nucleic acids research. 2007 Mar;35(7):e55. Available from: http://dx.doi.org/10.1093/nar/gkm106. doi:10.1093/nar/gkm106.

24. ECMA International. ECMAScript 2024 Language Specification; 2024. https://www.ecma-international.org/publications-and-standards/standards/ecma-262/.

25. van Rossum G, de Boer J. Interactively testing remote servers using the Python programming language. CWI Quarterly. 1991 Dec;4(4):283-304.

26. R Core Team. R: A Language and Environment for Statistical Computing. Vienna, Austria; 2024. Available from: https://www.R-project.org/.

27. Ousterhout JK, Jones K. Tcl and the Tk Toolkit. Addison-Wesley Professional Computing Series. Pearson Education; 2009.

28. Haas A, Rossberg A, Schuff DL, Titzer BL, Holman M, Gohman D, et al. Bringing the web up to speed with WebAssembly. ACM SIGPLAN Notices. 2017;52(6):185-200. doi:10.1145/3140587.3062363.

29. Pereira F, Azevedo F, Carvalho Â, Ribeiro GF, Budde MW, Johansson B. Pydna: a simulation and documentation tool for DNA assembly strategies using python. BMC bioinformatics. 2015 May;16(142):142. Available from: `http://dx.doi.org/10.1186/s12859-015-0544-x`. doi:10.1186/s12859-015-0544-x.

30. Cock PJA, Antao T, Chang JT, Chapman BA, Cox CJ, Dalke A, et al. Biopython: freely available Python tools for computational molecular biology and bioinformatics. Bioinformatics. 2009 Jun;25(11):1422-3. Available from: `http://dx.doi.org/10.1093/bioinformatics/btp163`. doi:10.1093/bioinformatics/btp163.

31. Bassi S, Gonzalez V. New checksum functions for Biopython. Nature Precedings. 2007 Jun:1-1. Available from: `https://www.nature.com/articles/npre.2007.278.1`. doi:10.1038/npre.2007.278.1.

32. Davis MW, Jorgensen EM. ApE, A Plasmid Editor: A Freely Available DNA Manipulation and Visualization Program. Frontiers in Bioinformatics. 2022;2. Available from: `https://www.frontiersin.org/article/10.3389/fbinf.2022.818619`. doi:10.3389/fbinf.2022.818619.

33. Bouras G, Grigson SR, Papudeshi B, Mallawaarachchi V, Roach MJ. Dnaapler: A tool to reorient circular microbial genomes. Journal of Open Source Software. 2024;9(93):5968. Available from: `https://doi.org/10.21105/joss.05968`. doi:10.21105/joss.05968.

PONE-D-24-51050

SEGUID v2: Extending SEGUID checksums for circular, linear, single- and double-stranded biological sequences

Journal Requirements

When submitting your revision, we need you to address these additional requirements.

1. Please ensure that your manuscript meets PLOS ONE's style requirements, including those for file naming.

The PLOS ONE style templates can be found at https://journals.plos.org/plosone/s/file?id=wjVg/PLOSOne_formatting_sample_main_body.pdf and https://journals.plos.org/plosone/s/file?id=ba62/PLOSOne_formatting_sample_title_authors_affiliations.pdf

Authors' response: We have converted our LaTeX document to use the PLOS ONE LaTeX template provided at https://journals.plos.org/plosone/s/latex. The style and layout of our PDF matches that of the example PDF provided by PLOS ONE.

Please note, our original PLOS ONE submission was based on our submission to PLOS Computational Biology, which then was transferred to PLOS ONE. Given that we now have migrated the manuscript to the PLOS ONE LaTeX template, it is not feasible for us to upload track changes. We're sorry about that, but we have clarified below what our changes are.

2. Please note that PLOS ONE has specific guidelines on code sharing for submissions in which author-generated code underpins the findings in the manuscript. In these cases, all author-generated code must be made available without restrictions upon publication of the work.

Please review our guidelines at https://journals.plos.org/plosone/s/materials-and-software-sharing#loc-sharing-code and ensure that your code is shared in a way that follows best practice and facilitates reproducibility and reuse.

Authors' response: Our source code is available under the GitHub organization https://github.com/seguid. We have made official releases of the relevant SEGUID implementations and obtained corresponding DOI:s from Zenodo:

- seguid-calculator, https://doi.org/10.5281/zenodo.17010386
- seguid-javascript, https://doi.org/10.5281/zenodo.17010380
- seguid-python, https://doi.org/10.5281/zenodo.17010394
- seguid-r, https://doi.org/10.5281/zenodo.17010375
- seguid-tcl, https://doi.org/10.5281/zenodo.17010377

All relevant data (sequence data examples) are within the manuscript and its Supporting Information files (ZIP file).

3. We note that the grant information you provided in the 'Funding Information' and 'Financial Disclosure' sections do not match.

Authors' response: We do not understand what you are referring to here, but if you mean that the 'Funding Information' entered in the PLOS ONE submission form did not match the information we previously had in the 'Acknowledgement' section, then the latter has been removed per your request (see below). If there is any other place were we should enter or specify a 'Financial Disclosure', please let us know - we do not see such a field in the PLOS ONE online submission.

4. Please note that funding information should not appear in the Acknowledgments section or other areas of your manuscript.

We will only publish funding information present in the Funding Statement section of the online submission form. Please remove any funding-related text from the manuscript.

Authors' response: We have removed the funding information from the Acknowledgments section in the manuscript. The first version stemmed from our original submission to PLOS Computational Biology, which then transferred the manuscript to PLOS ONE.

5. Thank you for uploading your study's underlying data set. Unfortunately, the repository you have noted in your Data Availability statement does not qualify as an acceptable data repository according to PLOS's standards.

At this time, please upload the minimal data set necessary to replicate your study's findings to a stable, public repository (such as figshare or Dryad) and provide us with the relevant URLs, DOIs, or accession numbers that may be used to access these data. For a list of recommended repositories and additional information on PLOS standards for data deposition, please see https://journals.plos.org/plosone/s/recommended-repositories.

Authors' response: All data used in the manuscript is available in the Supplementary Data ZIP file uploaded together with the manuscript. In the revision, we now explicitly specify "All relevant data are within the manuscript and its Supporting Information files." We have removed the reference to https://www.seguid.org originally used as a repository.

6. We note that Figures 8 and 9 in your submission contain copyrighted images.

All PLOS content is published under the Creative Commons Attribution License (CC BY 4.0), which means that the manuscript, images, and Supporting Information files will be freely available online, and any third party is permitted to access, download, copy, distribute, and use these materials in any way, even commercially, with proper attribution. For more information, see our copyright guidelines: http://journals.plos.org/plosone/s/licenses-and-copyright.

Authors' response: We understood this as there was a presence of commercial logotypes in these two figures, which has now been removed.

The old version of Figure 8, a screenshot of a presentation by co-author György Babnigg, contained the Argonne National Laboratory logotype, the Midwest Center for Structural Genomics (MCSG) logotype and the Protein Structure Initiative (PSI) logotype. These have been removed in the new Figure 8.

Figure 9 contain the "A plasmid Editor" (ApE) icon and the pydna icon. The latter was made up from parts for which we did not have ownership. The copyright holder for the new pydna icon is Björn Johansson, co-author to this manuscript. We have uploaded the requested forms.

Please upload the completed Content Permission Form or other proof of granted permissions as an ""Other"" file with your submission.

Authors' response: The remaining artifacts are copyrighted to co-authors of this manuscript. Given the above adjustments, we deemed this suggested statement unnecessary. If we need to sign Content Permission Forms for co-authors own work, please let us know.

8. We are unable to open your Supporting Information file [Pereira_etal_2024-SEGUIDv2-SupplData.zip]. Please kindly revise as necessary and re-upload.

Authors' response: We are not sure what is going on, but we suspect it is something on your end. We have verified that the ZIP file is indeed valid and complete. We did this by downloading the file from the PLOS portal via 'Author login' -> 'Submissions Needing Revision' -> 'Action Links' -> 'File Inventory' -> 'Download'. The downloaded file 'Pereira_etal_2024-SEGUIDv2-SupplData.zip' (Oct 30 2024 6:34PM) is 9,638 bytes large (md5sum ec75f42f41d3cede43cf471f210af8f4). Unzipping the file using `unzip Pereira_etal_2024-SEGUIDv2-SupplData.zip` extracts a directory `data/` with the expected nice sequence files.

Through this exercise, we noticed that one of the files uses a UniProt identifier whereas the others uses GenBank identifiers. For consistency, we renamed this file. Because of this, we have uploaded a slightly modified ZIP file, which we have also verified can be unzipped. The new file (9,658 bytes; md5sum afb9eca6f40ef7c2c8a6073e48d52835) contains:

```
$ unzip -v Pereira_etal_2024-SEGUIDv2-SupplData.zip
Archive:  Pereira_etal_2024-SEGUIDv2-SupplData.zip
 Length   Method    Size  Cmpr    Date    Time   CRC-32   Name
--------  ------  ------- ---- ---------- ----- --------  ----
       0  Stored        0   0% 2025-08-30 13:25 00000000  data/
       0  Stored        0   0% 2025-08-30 13:25 00000000  data/protein/
     199  Defl:N      173  13% 2025-08-30 13:25 3debe0ce  data/protein/AAF20475.1.fasta
     199  Defl:N      173  13% 2025-08-30 13:25 86373871  data/protein/AAF20481.1.fasta
       0  Stored        0   0% 2025-08-30 14:04 00000000  data/manual/
      22  Stored       22   0% 2025-08-30 13:25 512fa260  data/manual/AAD28494.1_p38-58-a
      22  Stored       22   0% 2025-08-30 13:25 fb35103b  data/manual/AAD28494.1_p38-58.a
       0  Stored        0   0% 2025-08-30 13:25 00000000  data/nucleotide/
    2776  Defl:N     1020  63% 2025-08-30 13:25 66cb8af7  data/nucleotide/M77789.2.fasta
    2777  Defl:N     1015  63% 2025-08-30 13:25 b476ded0  data/nucleotide/L09137.2.fasta
       0  Stored        0   0% 2025-08-30 14:05 00000000  data/nuccore/
    6527  Defl:N     2157  67% 2025-08-30 13:25 f647a886  data/nuccore/V00604.2.fasta
     914  Defl:N      397  57% 2025-08-30 13:25 5b81b0e9  data/nuccore/MW729338.1.fasta
    6555  Defl:N     2195  67% 2025-08-30 13:25 56d247cb  data/nuccore/NC_003287.2.fasta
--------          -------  ---                            -------
   19991             7174  64%                            14 files
```

Response to Reviewers

Reviewer 1

1. Clarify the Abstract: The abstract could be more structured. Consider clearly summarizing the background, objectives, methods, results, and conclusions to make it more reader-friendly.

Authors' response: The abstract is currently divided into Background, Objectives, Conclusions and Availability. As each part is rather short, we feel that adding more information here might muddle the abstract rather than clarify.

2. Enhance the Introduction: Add more context to the introduction by explaining the importance of SEGUID v2 in synthetic biology and how it addresses current challenges.

Authors' response: We added the following to the end of the introduction to address this point: "We believe that a stable checksum that describes any biological sequence, regardless of topology or strandedness, would streamline the exchange of information and biological materials within the synthetic biology community."

3. Define Technical Terms: Simplify or define technical terms like "checksums" and "topological differences" for readers who may not have a technical background.

Authors' response: We have changed the text in the Objectives section of the abstract:

"The original SEGUID, while effective for protein sequences and single-stranded DNA (ssDNA), is not suitable for circular and double-stranded DNA (dsDNA) due to topological differences."

to

"The original SEGUID, while effective for protein sequences and single-stranded DNA (ssDNA), is not suitable for circular DNA since there is no natural starting position nor for double-stranded DNA (dsDNA) since two separate sequences are present."

This change replaces the somewhat opaque phrase "topological differences". As for the term "checksum", we think that this is the simplest term available and it a well established term. We feel that synonyms like "hash function" would be harder to understand for readers from non-technical background.

4. Expand the Methodology: Provide more detail in the methodology section, especially about the algorithms used and how they ensure unique representations.

Authors' response: The new SEGUID checksums provide unique representation for all biological sequences described through two conventions:

1. The use of smallest string rotation as a starting point.
2. Using a semicolon to separate the 5′ -> 3′ Watson sequence and the 5′ -> 3′ Crick sequences prior to calculating checksums for double stranded sequences.

We feel that these points come across in the current manuscript.

5. Include Visual Aids: Diagrams or flowcharts illustrating the SEGUID v2 process would make the paper easier to understand and more engaging.

Authors' response: The manuscript currently contains ten figures serving this purpose. We do not believe adding more would enhance the reader's understanding. A re-review of the figures, did not reveal any superfluous figures that would improve the manuscript, if left out.

6. Discuss Limitations: Include a section that openly discusses SEGUID v2's limitations and suggests directions for future research.

Authors' response: We discuss advantages and drawbacks of the SHA-1 (Secure Hash Algorithm 1) algorithm and how this remains the best choice balancing length, resistance to collisions and availability across different computer systems. The 160-bit output provides a compact yet reliable identifier for data identification, and its widespread implementation has ensured compatibility across diverse systems. One notable application of SHA-1 is in the Git version control system, where it is used to uniquely identify file content.

7. Strengthen the Results Section: Add more quantitative data to back up the claims about the algorithm's effectiveness.

Authors' response: The first part of the Results section focuses heavily on the correctness of the algorithm and not so much on the effectiveness (speed, computational efficiency). We extensively discuss edge cases that may be of less importance currently, but this might change in the future. We believe that, at this point, correctness and completeness are more important, since changing the algorithm later could be costly. We think this is the correct priority as computationally efficient algorithms may be devised later as the need arises.

8. Add Comparative Analysis: Compare SEGUID v2 with other checksum algorithms to highlight its unique advantages.

Authors' response: As noted in Response 6, we believe this point is addressed. The novel nature of the checksum makes comparisons, other than the ones we made motivating the choice of the SHA-1 algorithm, of limited value.

9. Clarify Data Availability: Be specific about the types of data available and how readers can access them.

Authors' response: We direct the reader to the GitHub repository and the website associated with this project. We maintain the easy to remember seguid.org domain where we offer implementations for six popular programming languages. We also offer a very simple web interface as well as a SEGUID functionality in popular ApE DNA editor (Figure 7).

10. Improve Language Consistency: Ensure terms and terminology are used consistently, especially when discussing different types of sequences.

Authors' response: A careful reading of the manuscript did not reveal to us cases of inconsistent terminology.

11. Refine the Conclusion: Summarize the key findings and their implications for the field in a concise and impactful way.

Authors' response: Under section 6 Discussions, we conclude the main advantages compared to other methods. The details of these conclusions are listed in section 5 Applications.

12. Add References: Ensure all statements and claims are backed up with proper references, especially in the introduction and discussion sections.

Authors' response: We have 33 references that we feel adequately support the claims, using the oldest available references where several relevant ones were available.

13. Improve Formatting: Review the document to ensure consistent formatting across headings, subheadings, and reference styles.

Authors' response: The document is produced using LaTeX which ensures consistent formatting.

14. Clarify Acronyms: Define all acronyms when they first appear to make the content accessible to all readers.

Authors' response: The lists below show the acronyms used in the manuscript. The ones marked as revised below were changed in the revised manuscript to include a definition when first used:

- **SEGUID** - Defined on first use (no change)
- **SHA-1** - Defined as "Secure Hash Algorithm 1"; moved to first mention. (revised)
- **CRC** / **CRC-64** - Defined on first use (no change)
- **ssDNA** - Defined on first use (no change)
- **dsDNA** - Defined on first use (no change)
- **circRNA** - Defined on first use (no change)
- **GUI** - Definition moved to first mention (revised)
- **iGEM** - Defined on first use (revised)
- **CLI** - Defined on first use (no change)
- **API** - Defined on first use (no change)
- **URL** - Defined on first use (no change)
- **npm** - Defined on first use (revised)
- **PyPi** - Defined on first use (revised)
- **CRAN** - Defined on first use (revised)

15. Strengthen the Discussion: Connect the findings to existing literature in the discussion section to better contextualize the results.

Authors' response: The discussion is short on purpose. We feel that an overly long discussion section risks reiterating points already covered in the results section. As already stated, the SEGUID v2 does not really have competing solutions as no other prior checksum algorithm was able to handle a circular topology or distinguish between double and single stranded sequences in a non-ambiguous way.

16. Provide Practical Applications: Highlight how SEGUID v2 can be applied in real-world scenarios to make its significance clearer.

Authors' response: We use SEGUID as a tool in several ways. Many of the same sequence files are often shared among members in one of the research groups involved in this work and thus exist as copies on several computers. The checksums provide security against accidental errors and also an obvious way to communicate sequence updates. Here, the integration of the checksum in the DNA editor software helps. The second application is in the kind of teaching setting described in Figure 10 where correct results are calculated for correct answers to student exercise problems. The publication of this manuscript will hopefully help to widen the adoption of the checksum to other areas.

Reviewer 2

The authors are advised to carefully revise and resubmit the manuscript accordingly. The authors also also advised to provide the proper details of SHA-1 used in the paper. All the figures must be redraw again with a good quality.

Authors' response: We provide a reference for the widely used SHA-1: FIPS PUB 180-4 (2015). Secure hash standard (SHS). Federal Information Processing Standards Publication (FIPS PUB 180-4). This document describes the SHA-1 function in detail.

Figures 1-6, 9-10 are all *vector* graphics and are compiled into the PDF as such. In other words, they are provided in the highest resolution possible; it is possible to zoom in on the figures to any depth. If the critique is about accessibility such as low contrasts, please let us know, and we will revisit these figures.

Figures 7-8 are *bitmap* graphics. Figure 7a was a PNG of resolution 1245x609 pixels and Figure 7b a PNG of resolution 512x240 pixels (no change). We have updated Figure 7b to reflect the latest version of the ApE interface and such that it has resolution 902x479 pixels. The journal guidelines specifies that figures with multiple panels be meged into one figure. The Figures 7a and b were merged into a PNG of resolution 2400x600 pixels.

Figure 8 is a PNG of resolution 1949x997 pixels (no change).

If this is insufficient, please clarify which figures are not of acceptable quality and why.

Reviewer 3

"For example, the two proteins with clashing CRC-64 checksums (Figure 1) have distinct SEGUID checksums." I do not find this statement surprising or a good example of why SEGUID is superior.

Authors' response: We chose the CRC-64 checksum to illustrate the risk of checksum collisions. Since UniProt used this originally(*), and UniParc provides such checksums, CRC-64 serves as a real-world example of this problem. We also use CRC-64 as a segue for introducing the stronger SHA algorithms. We try to communicate that, although all checksum algorithms are subject to clashes, which we stated in our manuscript, the SHA-1 algorithm produces checksums for which the risk of a clash occurring unprovoked is so infinitesimally small as to be of no practical consequence. This is obviously not the case for the CRC-64 checksum.

(*) While revisiting this passage, we realized that our claim about UniProt was misleading. Although UniProt and UniParc still provides CRC-64 checksums, they are officially using MD5 checksums.

The authors have done a great job at providing multiple implementations in different programming languages, but I suspect a method like this will not be widely adopted by the community until it is incorporated into large databases such as UniProt. The authors should perhaps include this in the discussion.

Authors' response: We added "Since the checksum is already implemented in the popular DNA editor ApE, we believe this will encourage wider adoption by both researchers and web-facing applications." at the end to the end of the discussion to address this point.
