# Supplementary material for: SEGUID v2: Extending SEGUID checksums for circular, linear, single- and double-stranded biological sequences
Source: PLoS One. 2026 Aug 7;21(8):e0346164. doi: 10.1371/journal.pone.0346164 (PMC13450835; doi:10.1371/journal.pone.0346164)
Supplement: Supplementary file 5 [file pone.0346164.s005.pdf]

PONE-D-24-51050R1

SEGUID v2: Extending SEGUID checksums for circular, linear, single- and double-stranded biological sequences

Dear Dr. Bengtsson,

Thank you for submitting your manuscript to PLOS ONE. After careful consideration, we feel that it has merit but does not fully meet PLOS ONE's publication criteria as it currently stands. Therefore, we invite you to submit a revised version of the manuscript that addresses the points raised during the review process.

Please submit your revised manuscript by Dec 26 2025 11:59PM. If you will need more time than this to complete your revisions, please reply to this message or contact the journal office at plosone@plos.org. When you're ready to submit your revision, log on to https://www.editorialmanager.com/pone/ and select the 'Submissions Needing Revision' folder to locate your manuscript file.

Please include the following items when submitting your revised manuscript:

- A rebuttal letter that responds to each point raised by the academic editor and reviewer(s). You should upload this letter as a separate file labeled 'Response to Reviewers'.
- A marked-up copy of your manuscript that highlights changes made to the original version. You should upload this as a separate file labeled 'Revised Manuscript with Track Changes'.
- An unmarked version of your revised paper without tracked changes. You should upload this as a separate file labeled 'Manuscript'.

If you would like to make changes to your financial disclosure, please include your updated statement in your cover letter. Guidelines for resubmitting your figure files are available below the reviewer comments at the end of this letter.

If applicable, we recommend that you deposit your laboratory protocols in protocols.io to enhance the reproducibility of your results. Protocols.io assigns your protocol its own identifier (DOI) so that it can be cited independently in the future. For instructions see: https://journals.plos.org/plosone/s/submission-guidelines#loc-laboratory-protocols. Additionally, PLOS ONE offers an option for publishing peer-reviewed Lab Protocol articles, which describe protocols hosted on protocols.io. Read more information on sharing protocols at https://plos.org/protocols?utm_medium=editorial-email&utm_source=authorletters&utm_campaign=protocols.

We look forward to receiving your revised manuscript.

Kind regards,

Daniel Ioan Hunyadi, Ph.D. Academic Editor PLOS ONE

Journal Requirements

If the reviewer comments include a recommendation to cite specific previously published works, please review and evaluate these publications to determine whether they are relevant and should be cited. There is no requirement to cite these works unless the editor has indicated otherwise.

Please review your reference list to ensure that it is complete and correct. If you have cited papers that have been retracted, please include the rationale for doing so in the manuscript text, or remove these references and replace them with relevant current references. Any changes to the reference list should be mentioned in the rebuttal letter that accompanies your revised manuscript. If you need to cite a retracted article, indicate the article's retracted status in the References list and also include a citation and full reference for the retraction notice.

Reviewers' comments

Reviewer's Responses to Questions

Comments to the Author

1. If the authors have adequately addressed your comments raised in a previous round of review and you feel that this manuscript is now acceptable for publication, you may indicate that here to bypass the "Comments to the Author" section, enter your conflict of interest statement in the "Confidential to Editor" section, and submit your "Accept" recommendation.

Reviewer #4: *(No Response)*

Reviewer #5: *All comments have been addressed*

---

2. Is the manuscript technically sound, and do the data support the conclusions?

The manuscript must describe a technically sound piece of scientific research with data that supports the conclusions. Experiments must have been conducted rigorously, with appropriate controls, replication, and sample sizes. The conclusions must be drawn appropriately based on the data presented.

Reviewer #4: *Yes*

Reviewer #5: *Yes*

---

3. Has the statistical analysis been performed appropriately and rigorously?

Reviewer #4: *N/A*

Reviewer #5: *Yes*

4. Have the authors made all data underlying the findings in their manuscript fully available?

The PLOS Data policy requires authors to make all data underlying the findings described in their manuscript fully available without restriction, with rare exception (please refer to the Data Availability Statement in the manuscript PDF file). The data should be provided as part of the manuscript or its supporting information, or deposited to a public repository. For example, in addition to summary statistics, the data points behind means, medians and variance measures should be available. If there are restrictions on publicly sharing data—e.g. participant privacy or use of data from a third party—those must be specified.

Reviewer #4: *Yes*

Reviewer #5: *Yes*

---

5. Is the manuscript presented in an intelligible fashion and written in standard English?

PLOS ONE does not copyedit accepted manuscripts, so the language in submitted articles must be clear, correct, and unambiguous. Any typographical or grammatical errors should be corrected at revision, so please note any specific errors here.

Reviewer #4: *Yes*

Reviewer #5: *Yes*

Review Comments to the Author

Please use the space provided to explain your answers to the questions above. You may also include additional comments for the author, including concerns about dual publication, research ethics, or publication ethics. (Please upload your review as an attachment if it exceeds 20,000 characters)

Reviewer #4

Strengths of the manuscript are:

1. *Innovative and practical extension of the SEGUID algorithm to handle circular and double-stranded sequences.*

2. *Well-written and logically organized with clear methodological explanations.*

3. *Open-source, multi-language implementation enhances reproducibility and accessibility.*

   AUTHORS RESPONSE We thank Reviewer #4 for the positive assessment.

Limitations:

1. *Lacks quantitative benchmarking (e.g., runtime or collision testing) to support performance claims.*

   AUTHORS RESPONSE *The checksum algorithm has two parts, the assembly of a string and the calculation of the final hash using the established SHA-1 algorithm. The most computationally intensive parts would be finding the smallest rotation for circular sequences and the calculation of the SHA-1 checksum. We think raw performance in the calculation is of secondary importance to correctness and transparency at this stage. Future work could produce higher performance code, should the need arise. Regarding collisions, we think that we can rely on the wealth of data underpinning the references we make in the manuscript produced with the SHA-1 algorithm in use for over twenty years.*

2. *Mostly descriptive—no large-scale empirical validation on real genomic datasets.*

   AUTHORS RESPONSE We hope that well-maintained, correct and transparent code and descriptions of the algorithms will drive the adoption of SEGUID in more areas of biology and genomics. The large user base of the ApE plasmid editor already has access to the checksums in a user-friendly GUI.

3. *Discussion could better emphasize limitations and future directions.*

   AUTHORS RESPONSE We have added the following phrase to address potential limitations (line 458, new manuscript).

   "SEGUID offers a stable, unique checksum for any DNA molecule, regardless of strandedness (single or double). The stability relies on the correctness of the underlying sequence. This means that sequence updates have to be accommodated in the long-term management of prior checksum versions for a sequence."

4. *Some figures (8 & 9) are difficult to interpret due to small text and limited labeling.*

   AUTHORS RESPONSE Figure 8 was replaced with an image of finer resolution, making the text easier to read. Figure 9 is a piece of dynamic text, so visibility will be under the direct control of the typesetter who can choose the appropriate font for the journal.

Reviewer #5

Dear Authors,

You are doing excellent work, and this manuscript reflects significant effort and thoughtful research. The study is promising and makes a meaningful contribution to the field. However, a few essential points require further clarification and revision to enhance the overall quality and impact of the manuscript.

AUTHORS RESPONSE We thank Reviewer 5 for the positive appraisal.

The introduction clearly situates the work in the context of post-genomic data and synthetic biology. However, the motivation for creating SEGUID v2 could be emphasized earlier. Currently, the discussion of

plasmids, repositories, and checksums (lines 5–23) spans several paragraphs before clearly stating the specific gap SEGUID v2 fills.

Introduce the challenge of checksum limitations for DNA sequences and circular topologies (lines 43–53) earlier to frame the problem more sharply.

AUTHORS RESPONSE We have added the following phrase (line 23 in new manuscript)

“Checksums are widely used, but not directly applicable due to DNA topology. SEGUID v2, which is a topology-aware extension to SEGUID v1, addresses this limitation.”

Lines 24–42 cover CRC-64 and SEGUID (SHA-1-based) well, but there could be more explicit discussion of limitations in other bioinformatics checksum approaches or DNA-specific tools. For example, are there alternative hash-based methods used for DNA sequences in repositories or alignment tools? This would contextualize SEGUID v2 more rigorously.

AUTHORS RESPONSE To the best of our understanding, we have provided a thorough analysis of previous checksums used in bioinformatics methods. We think that sequence alignment and checksums solve different problems and prefer not to add material we deem less relevant or risk losing the pace of the text.

The manuscript should explicitly state what makes SEGUID v2 novel compared to SEGUID v1 and other existing sequence checksums. While it is implied that SEGUID v2 handles circular DNA and strandedness (lines 50–55), this is not highlighted as a unique contribution until lines 54–57. A clear sentence like “Unlike existing algorithms, SEGUID v2 can generate identical checksums for sequences regardless of strandedness or circularity” would strengthen the argument.

AUTHORS RESPONSE We have merged this suggestion with our previous sentence (line 56-59 in new manuscript) which now reads:

“Unlike existing algorithms, SEGUID v2 produces checksums for linear, circular, single- and double-stranded sequences of different types, including DNA, RNA, and amino-acid sequences in order to integrate synthetic biology resources and identify redundancies in existing databases.” We agree that this explicit statement makes the text clearer.

Terms like “topology” (line 44) and “strandedness” (line 60) may not be immediately apparent to all readers. A brief parenthetical definition could help: e.g., “topology (linear vs circular DNA)” or “strandedness (single vs double strand)” Grammar/Style:

AUTHORS RESPONSE We have added this suggestion in the new manuscript line 45 “(linear vs circular)” and line 61 “(single vs double strand)”.

Line 41: “With SHA-1, sequence collisions are improbable” → missing space after comma.

Consider more consistent use of plural/singular: e.g., “sequence collisions are improbable” (line 41) vs. “collision is unavoidable” (lines 33–34).

AUTHORS RESPONSE The sentence on line 35 in new manuscript (no change) already reads “While

collisions are unavoidable…" and the sentence on line 42 already reads "With SHA-1, sequence collisions are very unlikely," which use parallel plural.

The three challenges are clearly listed (lines 63–67). However, the sentence in line 62–63 ("The problem of defining a checksum for sequences of different kinds comprises three challenges") has a grammatical error: "comprises" → "comprises."

Rephrase to emphasize the problem statement more concisely, e.g., "Defining a checksum for diverse sequence types involves three main challenges." The explanation of linear and circular, single- and double-stranded sequences is comprehensive, but very dense. For example, the discussion of blunt-ended vs staggered double-stranded sequences (lines 92–119) may overwhelm readers.

AUTHORS RESPONSE We agree that this rephrasing is better. Line 69 (new manuscript) now reads "Defining a checksum for diverse sequence types involves three main challenges," which also corrects the ("comprise" vs "comprises") grammar mistake mentioned above.

We choose to keep the "comprehensive, but very dense" explanation as-is; we don't want to make the text even longer, yet we want to make sure it is complete, as this article will serve as the main go-to reference for the SEGUID v2 algorithm. The examples are strictly not needed, but are meant to remove any ambiguities that we might have overseen and to make the text mass less "dense".

Figures 2–5 (lines 88, 106, 137, 163) are referenced appropriately, but consider combining text and figure explanations to reduce repetition. The use of the lexicographical ordering explanation (lines 100–105, 115–117, 153–156, 171–179) is critical but could be simplified using a concise table or pseudocode.

AUTHORS RESPONSE A part of our intended readership consists of biologists, not familiar with pseudocode, so for this reason we prefer the text as-is. We think that the current text is also understandable for computational biologists or bioinformaticians.

Excellent explanation of SHA-1 choice, including trade-offs with SHA-2, SHA-224, SHA-512/t, and MD5 (lines 195–240). One primary concern: The section could be shortened without losing technical accuracy. Some sentences, especially regarding cryptographic considerations (lines 225–238), are more philosophical than method-focused and could be moved to a discussion section.

AUTHORS RESPONSE We believe that the discussion on clashes and security, although philosophical, has real and direct impact on the method. This discussion is of fundamental importance for anyone adopting SEGUID v2, e.g. we do not want to risk some user believing these checksums are cryptographically secure by themselves. For this reason we prefer the text as-is.

Overall Assessment:

Strengths:

Extremely thorough and technically precise. The methods cover all cases (linear/circular, single/double-stranded) and justify the choice of SHA-1 and Base64url. Figures are referenced effectively.

AUTHORS RESPONSE We thank Reviewer 5 for the positive assessment.

Weaknesses:

The section is dense, with long sentences and repeated explanations of lexicographic ordering. Some cryptographic discussion (lines 225–238) may be better suited to discussion than to methods. The section clearly highlights the robustness of SEGUID v2, including validation of input sequences (lines 320–321) and flexibility for non-standard alphabets (lines 323–329). Consider summarizing Table 2 (lines 319–320) and Table 3 (lines 338) in the main text more concisely. Currently, the detailed enumeration of symbols interrupts narrative flow—a brief mention with "see Table 2/3 for details" would improve readability. Strong coverage of minimal software implementations, API, and CLI examples (lines 340–360).

AUTHORS RESPONSE The current reference to Table 2 is (line 313, 314 new manuscript) "The SEGUID v2 algorithm is designed to support DNA, RNA, and proteins, but also sequences with a custom alphabet (Table 2)." We think that the content of Table 2 is in fact intuitive for someone with biological training and adding more details to either the relevant text or Table 2 would make both harder to read and interpret.

Suggest clarifying whether the command-line examples require input in a specific format (e.g., semicolon-separated Watson/Crick strands, lines 352–354).

AUTHORS RESPONSE We consider the CLI examples self-explanatory (lines 356–366, new manuscript), and adding information to them would risk making them less clear. The second and last example address the question directly, showing the two complementary strands separated by a semicolon. The full specification is in the code base of the different implementations and the test suites available via the SEGUID website.

Minor formatting:

remove trailing line breaks in command examples to enhance readability.

AUTHORS RESPONSE We do not understand this suggestion.

Clear separation between minimal API packages and higher-level implementations (lines 377–392).

The GUI section (lines 393–405) is well explained. Suggest including URLs for GUI tools (if publicly available) for reproducibility. Some repetition of the GUI purpose occurs in lines 393–405; it could be condensed. The use of SEGUID for teaching (lines 430–435) is compelling and well documented.

AUTHORS RESPONSE The URLs for the tools are included; the ApE URL is given as a footnote on page 14, and the seguid-calculator is available on the "SEGUID website" on line 408. We have clarified what the "SEGUID website" is and what the URL is in the sentence following the command-line examples. We avoid replicated use of explicit URLs in the text, which is why we write "SEGUID website" instead of "https://www.seguid.org" (which we define once and only once in the main text).

Minor issue:

line 430 starts mid-sentence; ensure proper context for clarity. Suggest adding a clear concluding sentence summarizing the utility of SEGUID in database management and education (lines 410–435). The authors clearly state the types of sequences supported (DNA, RNA, and proteins) and the invariance

properties (double-stranded orientation and circular rotation).

AUTHORS RESPONSE We changed the sentence on line 430 from “This kind of problem has a unique correct answer, making it possible to calculate checksums for the correct solution beforehand.” to “Since this kind of problem has a unique correct answer, checksums for the correct solution can be calculated beforehand” now on line 437 in new manuscript. We judge this line to be correct and complete.

We switched the previous sentence (Line 436) “Overall, it lowers the number of mistakes filed and the efforts on reviewing homework assignments.” to “Overall, precalculated SEGUID codes lower the number of mistakes filed and the efforts spent on reviewing homework assignments.” We believe this is clearer and more explicit.

Line 441: “also invariant to genome” is unclear — do you mean “genome orientation” or “sequence context within a genome”? Consider rephrasing for clarity.

AUTHORS RESPONSE We changed this phrase (line 443):

“SEGUID v2 is also invariant to genome and does not require the input to comprise known gene sequences.”

to

“SEGUID v2 is also independent of conventions or presence of specific sequences and does not require the input to comprise known gene sequences.”

This is to highlight that the presence of specific, known genes is not needed as for some other previously described tools.

Line 443: To enhance the discussion section, add the following reference: Hekmat A. Owaid, Mushtak T.S. Al-Ouqaili, M characterizationerization and genome sequencing of selected highly resistant clinical isolates of Pseudomonas aeruginosa and its association with the clustered regularly interspaced palindromic repeat/Cas system, Heliyon, Volume 11, Issue 1, 2025, e41670, ISSN 2405-8440, https://doi.org/10.1016/j.heliyon.2025.e41670.

AUTHORS RESPONSE We studied the given reference and it appears to present genome sequences for selected strains of Pseudomonas aeruginosa. These genomes are circular since the organism is prokaryotic and `cdseguid` could be used as a key for a database as well as versioning for these sequences. Apart from the potential application of SEGUID, we do not deem the addition of this reference will add value to the present manuscript.

References: Line 440 references Bouras et al. (2024) [33]; ensure this is added to the bibliography in proper format.

AUTHORS RESPONSE We have confirmed it is correct.

Line 452 mentions identifiers.org — consider adding a citation or URL for clarity.

AUTHORS RESPONSE The “identifiers.org” is the necessary and sufficient part of a URL; we added the URL protocol for completeness on line 467 in new manuscript.

Line 455: Conclusion should be objective, with further perspective, or should add at least a few sentences about future study/future perspective of it

AUTHORS RESPONSE The manuscript has no conclusion section, instead we sum up the work in the ‘Discussions’ section. We have added a statement “Future plans include continued maintenance and activities to drive wider use of the checksums.” on line 469 and 470 in new manuscript.

---

6. PLOS authors have the option to publish the peer review history of their article (what does this mean?). If published, this will include your full peer review and any attached files.

If you choose “no”, your identity will remain anonymous but your review may still be made public.

Do you want your identity to be public for this peer review? For information about this choice, including consent withdrawal, please see our Privacy Policy.

Reviewer #4: *Yes: Dr Iman Tajer Abdullah*

Reviewer #5: *Yes: Mushtak T.S.Al-Ouqaili*
