# Supplementary material for: SEGUID v2: Extending SEGUID checksums for circular, linear, single- and double-stranded biological sequences
Source: PLoS One. 2026 Aug 7;21(8):e0346164. doi: 10.1371/journal.pone.0346164 (PMC13450835; doi:10.1371/journal.pone.0346164)
Supplement: Peer review history

# Author comment: Author response to Decision Letter 0

**DOI:** 10.1371/journal.pone.0346164.r001

**Transfer Alert**

This paper was transferred from another journal. As a result, its full editorial history (including decision letters, peer reviews and author responses) may not be present.

7 Nov 2024

# Peer review: Decision Letter 0

**DOI:** 10.1371/journal.pone.0346164.r002

**Authors:** Daniel Ioan Hunyadi

23 Apr 2025

PONE-D-24-51050

SEGUID v2: Extending SEGUID checksums for circular, linear, single- and double-stranded biological sequences

PLOS ONE

Dear Dr. Bengtsson,

Thank you for submitting your manuscript to PLOS ONE. After careful consideration, we feel that it has merit but does not fully meet PLOS ONE’s publication criteria as it currently stands. Therefore, we invite you to submit a revised version of the manuscript that addresses the points raised during the review process.

Please submit your revised manuscript by Jun 07 2025 11:59PM. If you will need more time than this to complete your revisions, please reply to this message or contact the journal office at plosone@plos.org. When you're ready to submit your revision, log on to https://www.editorialmanager.com/pone/ and select the 'Submissions Needing Revision' folder to locate your manuscript file.. When you're ready to submit your revision, log on to https://www.editorialmanager.com/pone/ and select the 'Submissions Needing Revision' folder to locate your manuscript file.

Please include the following items when submitting your revised manuscript:

- A rebuttal letter that responds to each point raised by the academic editor and reviewer(s). You should upload this letter as a separate file labeled 'Response to Reviewers'.
- A marked-up copy of your manuscript that highlights changes made to the original version. You should upload this as a separate file labeled 'Revised Manuscript with Track Changes'.
- An unmarked version of your revised paper without tracked changes. You should upload this as a separate file labeled 'Manuscript'.

If you would like to make changes to your financial disclosure, please include your updated statement in your cover letter. Guidelines for resubmitting your figure files are available below the reviewer comments at the end of this letter.

If applicable, we recommend that you deposit your laboratory protocols in protocols.io to enhance the reproducibility of your results. Protocols.io assigns your protocol its own identifier (DOI) so that it can be cited independently in the future. For instructions see: https://journals.plos.org/plosone/s/submission-guidelines#loc-laboratory-protocols. Additionally, PLOS ONE offers an option for publishing peer-reviewed Lab Protocol articles, which describe protocols hosted on protocols.io. Read more information on sharing protocols at . Additionally, PLOS ONE offers an option for publishing peer-reviewed Lab Protocol articles, which describe protocols hosted on protocols.io. Read more information on sharing protocols at https://plos.org/protocols?utm_medium=editorial-email&utm_source=authorletters&utm_campaign=protocols..

We look forward to receiving your revised manuscript.

Kind regards,

Daniel Ioan Hunyadi, Ph.D.

Academic Editor

PLOS ONE

Journal Requirements:

When submitting your revision, we need you to address these additional requirements.

1. Please ensure that your manuscript meets PLOS ONE's style requirements, including those for file naming. The PLOS ONE style templates can be found at

https://journals.plos.org/plosone/s/file?id=wjVg/PLOSOne_formatting_sample_main_body.pdf and

https://journals.plos.org/plosone/s/file?id=ba62/PLOSOne_formatting_sample_title_authors_affiliations.pdf

2. Please note that PLOS ONE has specific guidelines on code sharing for submissions in which author-generated code underpins the findings in the manuscript. In these cases, all author-generated code must be made available without restrictions upon publication of the work.

Please review our guidelines at https://journals.plos.org/plosone/s/materials-and-software-sharing#loc-sharing-code and ensure that your code is shared in a way that follows best practice and facilitates reproducibility and reuse.

3. We note that the grant information you provided in the ‘Funding Information’ and ‘Financial Disclosure’ sections do not match.

When you resubmit, please ensure that you provide the correct grant numbers for the awards you received for your study in the ‘Funding Information’ section.

4. Please note that funding information should not appear in the Acknowledgments section or other areas of your manuscript. We will only publish funding information present in the Funding Statement section of the online submission form. Please remove any funding-related text from the manuscript.

5. Thank you for uploading your study's underlying data set. Unfortunately, the repository you have noted in your Data Availability statement does not qualify as an acceptable data repository according to PLOS's standards.

At this time, please upload the minimal data set necessary to replicate your study's findings to a stable, public repository (such as figshare or Dryad) and provide us with the relevant URLs, DOIs, or accession numbers that may be used to access these data. For a list of recommended repositories and additional information on PLOS standards for data deposition, please see https://journals.plos.org/plosone/s/recommended-repositories.

6. We note that Figures 8 and 9 in your submission contain copyrighted images. All PLOS content is published under the Creative Commons Attribution License (CC BY 4.0), which means that the manuscript, images, and Supporting Information files will be freely available online, and any third party is permitted to access, download, copy, distribute, and use these materials in any way, even commercially, with proper attribution. For more information, see our copyright guidelines: http://journals.plos.org/plosone/s/licenses-and-copyright.

We require you to either (1) present written permission from the copyright holder to publish these figures specifically under the CC BY 4.0 license, or (2) remove the figures from your submission:

1) You may seek permission from the original copyright holder of Figures 8 and 9 to publish the content specifically under the CC BY 4.0 license.

We recommend that you contact the original copyright holder with the Content Permission Form (http://journals.plos.org/plosone/s/file?id=7c09/content-permission-form.pdf) and the following text:

“I request permission for the open-access journal PLOS ONE to publish XXX under the Creative Commons Attribution License (CCAL) CC BY 4.0 (http://creativecommons.org/licenses/by/4.0/). Please be aware that this license allows unrestricted use and distribution, even commercially, by third parties. Please reply and provide explicit written permission to publish XXX under a CC BY license and complete the attached form.”

Please upload the completed Content Permission Form or other proof of granted permissions as an ""Other"" file with your submission.

In the figure caption of the copyrighted figure, please include the following text: “Reprinted from [ref] under a CC BY license, with permission from [name of publisher], original copyright [original copyright year].”

2) If you are unable to obtain permission from the original copyright holder to publish these figures under the CC BY 4.0 license or if the copyright holder’s requirements are incompatible with the CC BY 4.0 license, please either i) remove the figure or ii) supply a replacement figure that complies with the CC BY 4.0 license. Please check copyright information on all replacement figures and update the figure caption with source information.

If applicable, please specify in the figure caption text when a figure is similar but not identical to the original image and is therefore for illustrative purposes only.

7. Please include captions for your Supporting Information files at the end of your manuscript, and update any in-text citations to match accordingly. Please see our Supporting Information guidelines for more information: http://journals.plos.org/plosone/s/supporting-information.

8. We are unable to open your Supporting Information file [Pereira_etal_2024-SEGUIDv2-SupplData.zip]. Please kindly revise as necessary and re-upload.

[Note: HTML markup is below. Please do not edit.]

Reviewers' comments:

Reviewer's Responses to Questions

**Comments to the Author**

1. Is the manuscript technically sound, and do the data support the conclusions?

The manuscript must describe a technically sound piece of scientific research with data that supports the conclusions. Experiments must have been conducted rigorously, with appropriate controls, replication, and sample sizes. The conclusions must be drawn appropriately based on the data presented.

Reviewer #1: Yes

Reviewer #2: Partly

Reviewer #3: Yes

**********

2. Has the statistical analysis been performed appropriately and rigorously?

Reviewer #1: Yes

Reviewer #2: Yes

Reviewer #3: Yes

**********

3. Have the authors made all data underlying the findings in their manuscript fully available?

The PLOS Data policy requires authors to make all data underlying the findings described in their manuscript fully available without restriction, with rare exception (please refer to the Data Availability Statement in the manuscript PDF file). The data should be provided as part of the manuscript or its supporting information, or deposited to a public repository. For example, in addition to summary statistics, the data points behind means, medians and variance measures should be available. If there are restrictions on publicly sharing data—e.g. participant privacy or use of data from a third party—those must be specified.requires authors to make all data underlying the findings described in their manuscript fully available without restriction, with rare exception (please refer to the Data Availability Statement in the manuscript PDF file). The data should be provided as part of the manuscript or its supporting information, or deposited to a public repository. For example, in addition to summary statistics, the data points behind means, medians and variance measures should be available. If there are restrictions on publicly sharing data—e.g. participant privacy or use of data from a third party—those must be specified.

Reviewer #1: Yes

Reviewer #2: Yes

Reviewer #3: Yes

**********

4. Is the manuscript presented in an intelligible fashion and written in standard English?

PLOS ONE does not copyedit accepted manuscripts, so the language in submitted articles must be clear, correct, and unambiguous. Any typographical or grammatical errors should be corrected at revision, so please note any specific errors here.

Reviewer #1: Yes

Reviewer #2: Yes

Reviewer #3: Yes

**********

5. Review Comments to the Author

Please use the space provided to explain your answers to the questions above. You may also include additional comments for the author, including concerns about dual publication, research ethics, or publication ethics. (Please upload your review as an attachment if it exceeds 20,000 characters)

Reviewer #1: The research article introduces SEGUID v2, an enhanced version of the original SEGUID checksum algorithm, tailored to work with various biological sequences, including circular and double-stranded DNA (dsDNA). It underscores the critical role of checksums in synthetic biology, particularly in creating unique identifiers for DNA fragments to ensure data integrity. SEGUID v2 generates orientation- and rotation-invariant checksums, employing Base64url encoding to ensure compatibility with filenames and URLs. The algorithm effectively handles both linear and circular sequences, maintaining consistent checksums regardless of representation. Implemented in multiple programming languages and released under the MIT license, SEGUID v2 is easily accessible to researchers. The study highlights its potential to improve reproducibility and data management in biological research.

The work was very much interested to read. However, am suggesting following points to improve the manuscript.

1. Clarify the Abstract: The abstract could be more structured. Consider clearly summarizing the background, objectives, methods, results, and conclusions to make it more reader-friendly.

2. Enhance the Introduction: Add more context to the introduction by explaining the importance of SEGUID v2 in synthetic biology and how it addresses current challenges.

3. Define Technical Terms: Simplify or define technical terms like "checksums" and "topological differences" for readers who may not have a technical background.

4. Expand the Methodology: Provide more detail in the methodology section, especially about the algorithms used and how they ensure unique representations.

5. Include Visual Aids: Diagrams or flowcharts illustrating the SEGUID v2 process would make the paper easier to understand and more engaging.

6. Discuss Limitations: Include a section that openly discusses SEGUID v2's limitations and suggests directions for future research.

7. Strengthen the Results Section: Add more quantitative data to back up the claims about the algorithm's effectiveness.

8. Add Comparative Analysis: Compare SEGUID v2 with other checksum algorithms to highlight its unique advantages.

9. Clarify Data Availability: Be specific about the types of data available and how readers can access them.

10. Improve Language Consistency: Ensure terms and terminology are used consistently, especially when discussing different types of sequences.

11. Refine the Conclusion: Summarize the key findings and their implications for the field in a concise and impactful way.

12. Add References: Ensure all statements and claims are backed up with proper references, especially in the introduction and discussion sections.

13. Improve Formatting: Review the document to ensure consistent formatting across headings, subheadings, and reference styles.

14. Clarify Acronyms: Define all acronyms when they first appear to make the content accessible to all readers.

15. Strengthen the Discussion: Connect the findings to existing literature in the discussion section to better contextualize the results.

16. Provide Practical Applications: Highlight how SEGUID v2 can be applied in real-world scenarios to make its significance clearer.

Reviewer #2: The authors are advised to carefully revise and resubmit the manuscript accordingly. The authors also also advised to provide the proper details of SHA-1 used in the paper. All the figures must be redraw again with a good quality.

Reviewer #3: The manuscript by Pereira et al. introduces SEGUID v2, an extension of the original SEGUID (Sequence Globally Unique Identifier) checksum algorithm. The primary goal is to generate stable, unique, and universally applicable identifiers for a wider variety of biological sequences, addressing limitations of the original SEGUID and other checksum methods when dealing with the topological complexities of DNA and RNA. It achieves this by generating all possible representations of the same sequence and selecting the lexicographically smallest as the sequence to encode. The presented work provides a simple, smart, and computationally efficient solution to the problem of using checksums for non-linear biological sequences. The paper is well written and provides clear figures that aid in the understanding of the algorithm.

Comments:

- “For example, the two proteins with clashing CRC-64 checksums (Figure 1) have distinct SEGUID checksums.” I do not find this statement surprising or a good example of why SEGUID is superior.

- The authors have done a great job at providing multiple implementations in different programming languages, but I suspect a method like this will not be widely adopted by the community until it is incorporated into large databases such as UniProt. The authors should perhaps include this in the discussion.

**********

6. PLOS authors have the option to publish the peer review history of their article (what does this mean?). If published, this will include your full peer review and any attached files.). If published, this will include your full peer review and any attached files.

If you choose “no”, your identity will remain anonymous but your review may still be made public.

**Do you want your identity to be public for this peer review?**  For information about this choice, including consent withdrawal, please see our For information about this choice, including consent withdrawal, please see our Privacy Policy..

Reviewer #1: **Yes:**  RAJESH ARAJESH A

Reviewer #2: No

Reviewer #3: No

**********

[NOTE: If reviewer comments were submitted as an attachment file, they will be attached to this email and accessible via the submission site. Please log into your account, locate the manuscript record, and check for the action link "View Attachments". If this link does not appear, there are no attachment files.]

While revising your submission, please upload your figure files to the Preflight Analysis and Conversion Engine (PACE) digital diagnostic tool, https://pacev2.apexcovantage.com/. PACE helps ensure that figures meet PLOS requirements. To use PACE, you must first register as a user. Registration is free. Then, login and navigate to the UPLOAD tab, where you will find detailed instructions on how to use the tool. If you encounter any issues or have any questions when using PACE, please email PLOS at . PACE helps ensure that figures meet PLOS requirements. To use PACE, you must first register as a user. Registration is free. Then, login and navigate to the UPLOAD tab, where you will find detailed instructions on how to use the tool. If you encounter any issues or have any questions when using PACE, please email PLOS at figures@plos.org. Please note that Supporting Information files do not need this step.. Please note that Supporting Information files do not need this step.

# Author comment: Author response to Decision Letter 1

**DOI:** 10.1371/journal.pone.0346164.r003

23 Sep 2025

Please see 'Respond_to_Reviewers.pdf`. Thank you.

Attachment Submitted filename: Response_to_Reviewers.pdf

# Peer review: Decision Letter 1

**DOI:** 10.1371/journal.pone.0346164.r004

**Authors:** Daniel Ioan Hunyadi

11 Nov 2025

PONE-D-24-51050R1

SEGUID v2: Extending SEGUID checksums for circular, linear, single- and double-stranded biological sequences

PLOS ONE

Dear Dr. Bengtsson,

Thank you for submitting your manuscript to PLOS ONE. After careful consideration, we feel that it has merit but does not fully meet PLOS ONE’s publication criteria as it currently stands. Therefore, we invite you to submit a revised version of the manuscript that addresses the points raised during the review process.

Please submit your revised manuscript by Dec 26 2025 11:59PM. If you will need more time than this to complete your revisions, please reply to this message or contact the journal office at plosone@plos.org. When you're ready to submit your revision, log on to https://www.editorialmanager.com/pone/ and select the 'Submissions Needing Revision' folder to locate your manuscript file.. When you're ready to submit your revision, log on to https://www.editorialmanager.com/pone/ and select the 'Submissions Needing Revision' folder to locate your manuscript file.

Please include the following items when submitting your revised manuscript:

- A rebuttal letter that responds to each point raised by the academic editor and reviewer(s). You should upload this letter as a separate file labeled 'Response to Reviewers'.
- A marked-up copy of your manuscript that highlights changes made to the original version. You should upload this as a separate file labeled 'Revised Manuscript with Track Changes'.
- An unmarked version of your revised paper without tracked changes. You should upload this as a separate file labeled 'Manuscript'.

-->If you would like to make changes to your financial disclosure, please include your updated statement in your cover letter. Guidelines for resubmitting your figure files are available below the reviewer comments at the end of this letter.

If applicable, we recommend that you deposit your laboratory protocols in protocols.io to enhance the reproducibility of your results. Protocols.io assigns your protocol its own identifier (DOI) so that it can be cited independently in the future. For instructions see: https://journals.plos.org/plosone/s/submission-guidelines#loc-laboratory-protocols. Additionally, PLOS ONE offers an option for publishing peer-reviewed Lab Protocol articles, which describe protocols hosted on protocols.io. Read more information on sharing protocols at . Additionally, PLOS ONE offers an option for publishing peer-reviewed Lab Protocol articles, which describe protocols hosted on protocols.io. Read more information on sharing protocols at https://plos.org/protocols?utm_medium=editorial-email&utm_source=authorletters&utm_campaign=protocols..

We look forward to receiving your revised manuscript.

Kind regards,

Daniel Ioan Hunyadi, Ph.D.

Academic Editor

PLOS ONE

Journal Requirements:

If the reviewer comments include a recommendation to cite specific previously published works, please review and evaluate these publications to determine whether they are relevant and should be cited. There is no requirement to cite these works unless the editor has indicated otherwise.

Please review your reference list to ensure that it is complete and correct. If you have cited papers that have been retracted, please include the rationale for doing so in the manuscript text, or remove these references and replace them with relevant current references. Any changes to the reference list should be mentioned in the rebuttal letter that accompanies your revised manuscript. If you need to cite a retracted article, indicate the article’s retracted status in the References list and also include a citation and full reference for the retraction notice.

[Note: HTML markup is below. Please do not edit.]

Reviewers' comments:

Reviewer's Responses to Questions

**Comments to the Author**

1. If the authors have adequately addressed your comments raised in a previous round of review and you feel that this manuscript is now acceptable for publication, you may indicate that here to bypass the “Comments to the Author” section, enter your conflict of interest statement in the “Confidential to Editor” section, and submit your "Accept" recommendation.

Reviewer #4: (No Response)

Reviewer #5: All comments have been addressed

**********

2. Is the manuscript technically sound, and do the data support the conclusions?

The manuscript must describe a technically sound piece of scientific research with data that supports the conclusions. Experiments must have been conducted rigorously, with appropriate controls, replication, and sample sizes. The conclusions must be drawn appropriately based on the data presented.

Reviewer #4: Yes

Reviewer #5: Yes

**********

3. Has the statistical analysis been performed appropriately and rigorously?

Reviewer #4: N/A

Reviewer #5: Yes

**********

4. Have the authors made all data underlying the findings in their manuscript fully available?

The PLOS Data policy requires authors to make all data underlying the findings described in their manuscript fully available without restriction, with rare exception (please refer to the Data Availability Statement in the manuscript PDF file). The data should be provided as part of the manuscript or its supporting information, or deposited to a public repository. For example, in addition to summary statistics, the data points behind means, medians and variance measures should be available. If there are restrictions on publicly sharing data—e.g. participant privacy or use of data from a third party—those must be specified.requires authors to make all data underlying the findings described in their manuscript fully available without restriction, with rare exception (please refer to the Data Availability Statement in the manuscript PDF file). The data should be provided as part of the manuscript or its supporting information, or deposited to a public repository. For example, in addition to summary statistics, the data points behind means, medians and variance measures should be available. If there are restrictions on publicly sharing data—e.g. participant privacy or use of data from a third party—those must be specified.

Reviewer #4: Yes

Reviewer #5: Yes

**********

5. Is the manuscript presented in an intelligible fashion and written in standard English?

PLOS ONE does not copyedit accepted manuscripts, so the language in submitted articles must be clear, correct, and unambiguous. Any typographical or grammatical errors should be corrected at revision, so please note any specific errors here.

Reviewer #4: Yes

Reviewer #5: Yes

**********

6. Review Comments to the Author

Please use the space provided to explain your answers to the questions above. You may also include additional comments for the author, including concerns about dual publication, research ethics, or publication ethics. (Please upload your review as an attachment if it exceeds 20,000 characters)

Reviewer #4: Strengths of the manuscript are:

1. Innovative and practical extension of the SEGUID algorithm to handle circular and double-stranded sequences.

2. Well-written and logically organized with clear methodological explanations.

3. Open-source, multi-language implementation enhances reproducibility and accessibility.

Limitations:

1. Lacks quantitative benchmarking (e.g., runtime or collision testing) to support performance claims.

2. Mostly descriptive—no large-scale empirical validation on real genomic datasets.

3. Discussion could better emphasize limitations and future directions.

4. Some figures (8 &9) are difficult to interpret due to small text and limited labelling.

Reviewer #5: Dear Authors,

You are doing excellent work, and this manuscript reflects significant effort and thoughtful research. The study is promising and makes a meaningful contribution to the field. However, a few essential points require further clarification and revision to enhance the overall quality and impact of the manuscript.

The introduction clearly situates the work in the context of post-genomic data and synthetic biology. However, the motivation for creating SEGUID v2 could be emphasized earlier. Currently, the discussion of plasmids, repositories, and checksums (lines 5–23) spans several paragraphs before clearly stating the specific gap SEGUID v2 fills.

Introduce the challenge of checksum limitations for DNA sequences and circular topologies (lines 43–53) earlier to frame the problem more sharply.

Lines 24–42 cover CRC-64 and SEGUID (SHA-1-based) well, but there could be more explicit discussion of limitations in other bioinformatics checksum approaches or DNA-specific tools. For example, are there alternative hash-based methods used for DNA sequences in repositories or alignment tools? This would contextualize SEGUID v2 more rigorously.

The manuscript should explicitly state what makes SEGUID v2 novel compared to SEGUID v1 and other existing sequence checksums. While it is implied that SEGUID v2 handles circular DNA and strandedness (lines 50–55), this is not highlighted as a unique contribution until lines 54–57. A clear sentence like “Unlike existing algorithms, SEGUID v2 can generate identical checksums for sequences regardless of strandedness or circularity” would strengthen the argument.

Terms like “topology” (line 44) and “strandedness” (line 60) may not be immediately apparent to all readers. A brief parenthetical definition could help: e.g., “topology (linear vs circular DNA)” or “strandedness (single vs double strand)”

Grammar/Style:

Line 41: “With SHA-1, sequence collisions are improbable” → missing space after comma.

Consider more consistent use of plural/singular: e.g., “sequence collisions are improbable” (line 41) vs. “collision is unavoidable” (lines 33–34).

The three challenges are clearly listed (lines 63–67). However, the sentence in line 62–63 (“The problem of defining a checksum for sequences of different kinds comprises three challenges”) has a grammatical error: “comprises” → “comprises.”

Rephrase to emphasize the problem statement more concisely, e.g., “Defining a checksum for diverse sequence types involves three main challenges.”

The explanation of linear and circular, single- and double-stranded sequences is comprehensive, but very dense. For example, the discussion of blunt-ended vs staggered double-stranded sequences (lines 92–119) may overwhelm readers.

Figures 2–5 (lines 88, 106, 137, 163) are referenced appropriately, but consider combining text and figure explanations to reduce repetition.

The use of the lexicographical ordering explanation (lines 100–105, 115–117, 153–156, 171–179) is critical but could be simplified using a concise table or pseudocode.

Excellent explanation of SHA-1 choice, including trade-offs with SHA-2, SHA-224, SHA-512/t, and MD5 (lines 195–240).

One primary concern: The section could be shortened without losing technical accuracy. Some sentences, especially regarding cryptographic considerations (lines 225–238), are more philosophical than method-focused and could be moved to a discussion section.

Overall Assessment:

Strengths: Extremely thorough and technically precise. The methods cover all cases (linear/circular, single/double-stranded) and justify the choice of SHA-1 and Base64url. Figures are referenced effectively.

Weaknesses: The section is dense, with long sentences and repeated explanations of lexicographic ordering. Some cryptographic discussion (lines 225–238) may be better suited to discussion than to methods.

The section clearly highlights the robustness of SEGUID v2, including validation of input sequences (lines 320–321) and flexibility for non-standard alphabets (lines 323–329).

Consider summarizing Table 2 (lines 319–320) and Table 3 (lines 338) in the main text more concisely. Currently, the detailed enumeration of symbols interrupts narrative flow—a brief mention with “see Table 2/3 for details” would improve readability.

Strong coverage of minimal software implementations, API, and CLI examples (lines 340–360).

Suggest clarifying whether the command-line examples require input in a specific format (e.g., semicolon-separated Watson/Crick strands, lines 352–354).

Minor formatting: remove trailing line breaks in command examples to enhance readability.

Clear separation between minimal API packages and higher-level implementations (lines 377–392).

The GUI section (lines 393–405) is well explained. Suggest including URLs for GUI tools (if publicly available) for reproducibility.

Some repetition of the GUI purpose occurs in lines 393–405; it could be condensed.

The use of SEGUID for teaching (lines 430–435) is compelling and well documented.

Minor issue: line 430 starts mid-sentence; ensure proper context for clarity.

Suggest adding a clear concluding sentence summarizing the utility of SEGUID in database management and education (lines 410–435).

The authors clearly state the types of sequences supported (DNA, RNA, and proteins) and the invariance properties (double-stranded orientation and circular rotation).

Line 441: “also invariant to genome” is unclear — do you mean “genome orientation” or “sequence context within a genome”? Consider rephrasing for clarity.

Line 443: To enhance the discussion section, add the following reference: Hekmat A. Owaid, Mushtak T.S. Al-Ouqaili, Mcharacterizationerization and genome sequencing of selected highly resistant clinical isolates of Pseudomonas aeruginosa and its association with the clustered regularly interspaced palindromic repeat/Cas system, Heliyon, Volume 11, Issue 1, 2025, e41670, ISSN 2405-8440, https://doi.org/10.1016/j.heliyon.2025.e41670.

References:

Line 440 references Bouras et al. (2024) [33]; ensure this is added to the bibliography in proper format.

Line 452 mentions identifiers.org — consider adding a citation or URL for clarity.

Line 455: Conclusion should be objective, with further perspective, or should add at least a few sentences about future study/future perspective of it

**********

7. PLOS authors have the option to publish the peer review history of their article (what does this mean?). If published, this will include your full peer review and any attached files.). If published, this will include your full peer review and any attached files.

If you choose “no”, your identity will remain anonymous but your review may still be made public.

**Do you want your identity to be public for this peer review?**  For information about this choice, including consent withdrawal, please see our For information about this choice, including consent withdrawal, please see our Privacy Policy..

Reviewer #4: **Yes:**  Dr Iman Tajer AbdullahDr Iman Tajer Abdullah

Reviewer #5: **Yes:**  Mushtak T.S.Al-OuqailiMushtak T.S.Al-Ouqaili

**********

[NOTE: If reviewer comments were submitted as an attachment file, they will be attached to this email and accessible via the submission site. Please log into your account, locate the manuscript record, and check for the action link "View Attachments". If this link does not appear, there are no attachment files.]

To ensure your figures meet our technical requirements, please review our figure guidelines: https://journals.plos.org/plosone/s/figures

You may also use PLOS’s free figure tool, NAAS, to help you prepare publication quality figures: https://journals.plos.org/plosone/s/figures#loc-tools-for-figure-preparation.

NAAS will assess whether your figures meet our technical requirements by comparing each figure against our figure specifications.

Attachment Submitted filename: Reviewer comments- PONE-D-24-51050_R1.pdf

# Author comment: Author response to Decision Letter 2

**DOI:** 10.1371/journal.pone.0346164.r005

2 Mar 2026

Please see 'Response_to_Reviews_2.pdf'

Attachment Submitted filename: Response_to_Reviewers_2.pdf

# Editor report: Decision Letter 2

**DOI:** 10.1371/journal.pone.0346164.r006

**Authors:** Daniel Ioan Hunyadi

16 Mar 2026

SEGUID v2: Extending SEGUID checksums for circular, linear, single- and double-stranded biological sequences

PONE-D-24-51050R2

Dear Dr. Bengtsson,

We’re pleased to inform you that your manuscript has been judged scientifically suitable for publication and will be formally accepted for publication once it meets all outstanding technical requirements.

Within one week, you’ll receive an e-mail detailing the required amendments. When these have been addressed, you’ll receive a formal acceptance letter and your manuscript will be scheduled for publication.

An invoice will be generated when your article is formally accepted. Please note, if your institution has a publishing partnership with PLOS and your article meets the relevant criteria, all or part of your publication costs will be covered. Please make sure your user information is up-to-date by logging into Editorial Manager at Editorial Manager® and clicking the ‘Update My Information' link at the top of the page. For questions related to billing, please contact  and clicking the ‘Update My Information' link at the top of the page. For questions related to billing, please contact billing support..

If your institution or institutions have a press office, please notify them about your upcoming paper to help maximize its impact. If they’ll be preparing press materials, please inform our press team as soon as possible -- no later than 48 hours after receiving the formal acceptance. Your manuscript will remain under strict press embargo until 2 pm Eastern Time on the date of publication. For more information, please contact onepress@plos.org.

Kind regards,

Daniel Ioan Hunyadi, Ph.D.

Academic Editor

PLOS One

Additional Editor Comments (optional):

Reviewers' comments:

# Editor report: Acceptance letter

**DOI:** 10.1371/journal.pone.0346164.r007

**Authors:** Daniel Ioan Hunyadi

PONE-D-24-51050R2

PLOS One

Dear Dr. Bengtsson,

I'm pleased to inform you that your manuscript has been deemed suitable for publication in PLOS One. Congratulations! Your manuscript is now being handed over to our production team.

At this stage, our production department will prepare your paper for publication. This includes ensuring the following:

* All references, tables, and figures are properly cited

* All relevant supporting information is included in the manuscript submission,

* There are no issues that prevent the paper from being properly typeset

You will receive further instructions from the production team, including instructions on how to review your proof when it is ready. Please keep in mind that we are working through a large volume of accepted articles, so please give us a few days to review your paper and let you know the next and final steps.

Lastly, if your institution or institutions have a press office, please let them know about your upcoming paper now to help maximize its impact. If they'll be preparing press materials, please inform our press team within the next 48 hours. Your manuscript will remain under strict press embargo until 2 pm Eastern Time on the date of publication. For more information, please contact onepress@plos.org.

You will receive an invoice from PLOS for your publication fee after your manuscript has reached the completed accept phase. If you receive an email requesting payment before acceptance or for any other service, this may be a phishing scheme. Learn how to identify phishing emails and protect your accounts at https://explore.plos.org/phishing.

If we can help with anything else, please email us at customercare@plos.org.

Thank you for submitting your work to PLOS ONE and supporting open access.

Kind regards,

PLOS ONE Editorial Office Staff

on behalf of

Dr. Daniel Ioan Hunyadi

Academic Editor

PLOS One
